# α2-3 Sialic acid binding and uptake by human monocyte-derived dendritic cells alters metabolism and cytokine release and initiates tolerizing T cell programming

**DOI:** 10.1093/immadv/ltab012

**Published:** 2021-06-09

**Authors:** Joyce Lübbers, Rui-Jún Eveline Li, Friederike S Gorki, Sven C M Bruijns, Ashley Gallagher, Hakan Kalay, Martino Ambrosini, Douwe Molenaar, Jan Van den Bossche, Sandra J van Vliet, Yvette van Kooyk

**Affiliations:** 1 Amsterdam UMC, Vrije Universiteit Amsterdam, Department of Molecular Cell Biology and Immunology, Cancer Center Amsterdam, Amsterdam Infection and Immunity Institute, Amsterdam, The Netherlands; 2 Institute of Innate Immunity, University Hospital Bonn, University of Bonn, Bonn, Germany; 3 Systems Bioinformatics, Vrije University Amsterdam, Amsterdam, The Netherlands

**Keywords:** dendritic cells, tolerance, glycolysis, regulatory T cells, Sialic acids and Siglecs

## Abstract

Dendritic cells (DCs) are key in the initiation of the adaptive T cell responses to tailor adequate immunity that corresponds to the type of pathogen encountered. Oppositely, DCs control the resolution phase of inflammation and are able to induce tolerance after receiving anti-inflammatory cytokines or upon encounter of self-associated molecular patterns, such as α2-3 linked sialic acid (α2-3sia).

Objective: We here investigated whether α2-3sia, that bind immune inhibitory Siglec receptors, would alter signaling and reprogramming of LPS-stimulated human monocyte-derived DCs (moDCs).

Methods and Results: Transcriptomic analysis of moDCs stimulated with α2-3sia-conjugated dendrimers revealed differentially expressed genes related to metabolic pathways, cytokines, and T cell differentiation. An increase in genes involved in ATPase regulator activity, oxidoreductase activity, and glycogen metabolic processes was detected. Metabolic extracellular flux analysis confirmed a more energetic moDC phenotype upon α2-3sia binding as evidenced by an increase in both glycolysis and mitochondrial oxidative phosphorylation. T_H_1 differentiation promoting genes *IFNL* and *IL27*, were significantly downregulated in the presence of α2-3sia. Functional assays confirmed that α2-3sia binding to moDCs induced phosphorylation of Siglec-9, reduced production of inflammatory cytokines IL-12 and IL-6, and increased IL-10. Surprisingly, α2-3sia-differentiated moDCs promoted FoxP3^+^CD25^+/-^CD127^-^ regulatory T cell differentiation and decreased FoxP3^-^CD25^-^CD127^-^ effector T cell proliferation.

Conclusions: In conclusion, we demonstrate that α2-3sia binding to moDCs, phosphorylates Siglec-9, alters metabolic pathways, cytokine signaling, and T cell differentiation processes in moDCs and promotes regulatory T cells. The sialic acid-Siglec axis on DCs is therefore, a novel target to induce tolerance and to explore for immunotherapeutic interventions aimed to restore inflammatory processes.

## Graphical Abstract

**Graphical Abstract:**
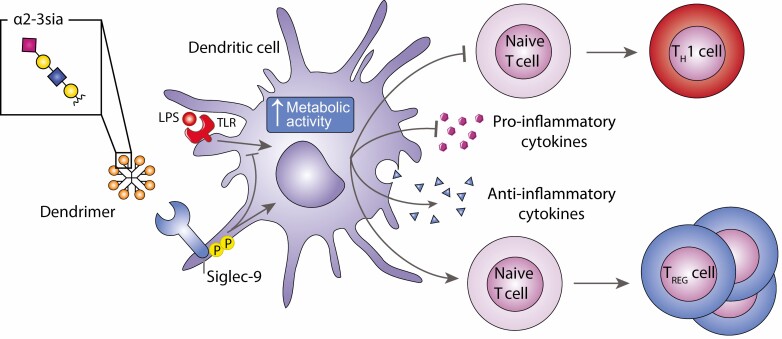

## Introduction

Dendritic cells (DCs) are antigen-presenting cells that sample their environment for pathogen entry using pattern recognition receptors (PRRs), such as Toll-like receptors (TLRS) and C-type lectin receptors [1]. Both receptors decode pathogen characteristics, such as pathogen-associated molecular patterns and pathogenic glycans, leading to pathogen uptake by the DCs and the induction of a tailored T cell response. Furthermore, DCs control the resolution phase of inflammation and are equipped to induce tolerance to self-antigens through initiation of regulatory T cell proliferation or the induction of anergy or deletion of antigen-specific effector T cells [2]. These tolerogenic DCs are therefore considered a promising therapeutic target for treatment of autoimmune diseases, like rheumatoid arthritis and allergies [3]. Compounds, such as dexamethasone, interleukin (IL)-10 or vitamin D3, have been shown to interfere with the *in-vitro* induction of T cell activation and T cell skewing by tolerogenic DCs [4, 5]. However, it remains questionable whether self-associated molecular patterns (SAMPs) presented on tissue antigens can induce tolerogenic differentiation processes in DCs to induce immune tolerance.

Sialic acids are considered as SAMPs and serve as ligands for Sialic acid binding immunoglobulin type lectins (Siglecs) [6]. Sialic acids are negatively charged monosaccharides that decorate terminal positions of glycoproteins on cell surfaces, and can take on multiple α-linkages that are formed between the sialic acid and underlying carbohydrate residue (commonly the C-3, -6, or -8 position). Members of the Siglec family, including Siglec-2, -3, -7, and -9, have been associated with inhibition of immune cell activation upon binding their ligands, sialic acids. Through immunoreceptor tyrosine-based inhibitory motifs (ITIMs) in the intracellular domain of the receptor, these Siglecs are able to recruit SHP phosphatases, resulting in de-phosphorylation and overall downregulation of activating signaling cascades [7–9]. Human moDCs are known to express the inhibitory Siglec receptors Siglec-7, Siglec-9, and Siglec-10 [10], and have low expression of Siglec-1 (CD169), which can be upregulated by interferon (IFN) alpha. Recently, we demonstrated that modification of antigens with a Siglec-E ligand (α2-3sia) induced tolerogenic DCs in mice [11]. Targeting an antigen to mouse Siglec-E, the homologue of human Siglec-7 and Siglec-9, enabled murine DCs to induce antigen-specific regulatory T cells, which inhibited CD8^+^ effector T cells, both *ex-vivo* and *in-vivo* [11,12].

Sialic acids are commonly found determinants on cells or proteins (IgG) and sialic acid overexpression or hypersialylation is often exploited by pathogens or cancer cells to evade the immune response. Overexpression of sialic acids is observed in multiple tumor types, including head and neck, oral, pancreas, and breast cancer [13–16]. Analysis of immune networks revealed that hypersialylation of melanoma cells in mouse tumor models increased the presence of regulatory T cells and reduced effector T cells, mirrored by an increase in tumor growth [12, 17]. Moreover, we recently showed that increased a2,3 sialylation in pancreas cancer lead to Siglec 9 mediated modulation of myeloid cells [16]). Pathogens, such as *Campylobacter jejuni and Group B Streptococcus* carry high levels of sialic acids on the envelope glycoprotein or bacterial capsule, respectively [18, 19]. Most pathogens are unable to synthesize *de novo* sialic acids, and are therefore at the disposal of host sialic acids as a source, or to apply sialic acid mimicry [20–22]. Nonetheless, pathogens can harbor various glycans that may simultaneously trigger other PRRs, hereby affecting DC receptor-mediated signaling responses.

Recent research highlights that immune cell activation is associated with metabolic changes that regulate immune cell phenotype and function [23]. Resting human moDCs have an active oxidative phosphorylation (OXPHOS), driven by the tricarboxylic acid (TCA) cycle for ATP generation. Intracellular glycogen supports the basal glycolytic demand for mitochondrial respiration. Upon activation with a toll like receptor ligand, the moDCs switch to glycolysis and increase glucose consumption [24]. In contrast, Vitamin D3-stimulated tolerogenic DCs have an increased OXPHOS and glycolysis, as indicated by enhanced oxygen consumption rate (OCR) and extracellular acidification rate (ECAR) [25].

In this study, we investigate the sialic acid-Siglec axis, as immunotherapeutic potential to alter human moDCs toward a tolerogenic differentiation program. We demonstrate that α2-3sia and LPS stimulated moDCs alter their genes involved in metabolic processes, cytokine production and reprogram DCs to enhance Treg:T_H_1 ratio balance. These data not only highlight the importance of glycan sensing by DCs to control both inflammation and the resolution phase, but also emphasize their potential to exploit as immunotherapeutic target for tolerance induction.

## Material and methods

### Isolation and culture of primary human monocyte-derived DCs

Ficoll gradient centrifugation and CD14-positive MACS bead (Miltenyi Biotec, CA) isolated monocytes from healthy donor buffy coats (Sanquin, reference: S03.0023-XT) were cultured for five days at 37°C, 5% CO_2_ in RPMI 1640 supplemented with 10% FCS (fetal calf serum), 100 U/ml penicillin, 100 U/ml streptomycin, 2 mM glutamine, 500 U/ml interleukin (IL)-4 and 800 U/ml granulocyte-macrophage colony stimulating factor to generate monocyte-derived dendritic cells (moDCs), as previously described [19].

### α2-3 sialic acid dendrimer synthesis

The glycodendrimers were synthesized by conjugation of a generation 2.0 PAMAM dendrimer with cystamine core (Sigma-Aldrich) via reductive amination of the free amino moieties. Approximately 20 equivalents per dendrimer of 3′-Sialyl-N-acetyllactosamine (for the transcriptomic analysis only), LS-Tetrasaccharide d (LSTd, in all other experiments; both from Elicityl) or *D*-(+)-Galactose (negative control, Sigma-Aldrich) were dissolved in Dimethylsulphoxide and acetic acid (8:2) to generate the α2-3sia and control dendrimer, respectively. Per dendrimer 160 equivalents of the 2-Methylpyridine borane complex (Sigma-Aldrich) were added in a total volume of 200 μl and incubated for 2 hours at 65°C with frequent vortexing. Disposable PD10 desalting columns (GE Healthcare) in 50 mM Ammonium Formate pH 4.4 (NH_4_HCO_3_) were used to purify the dendrimers. Subsequent lyophilization cycles were used to retrieve the glycodendrimers, followed by validation using LC-MS.

### α2-3 sialic acid-conjugate validation

The α2-3 sialic acid and control dendrimers were validated for α2-3 sialic acid presence with a binding ELISA to *Maackia Amurensis* Lectin I (MAL-I) (Vector Laboratories, Peterborough, UK). NUNC maxisorb plates (RosKilde) were coated overnight at 4°C with 5 µM of the products. The wells were subsequently blocked for 2 hours at room temperature with carbo-free blocking buffer (Vector, SP5040). Incubation with the biotinylated MAL-I and peroxidase-labeled streptavidin (Sigma-Aldrich) allowed spectrophotometric quantification of the binding with 3,3′,5,5′-tetramethylbenzidine (Sigma-Aldrich) at 450 nm on the iMark^TM^ Microplate Absorbance Reader (Bio-RAD).

### MoDC stimulation and RNA isolation for RNA sequencing

Day 4 moDCs were seeded (1 ×·10^5^ cells/well) in a sterile 96-well NUNC plate in RPMI-1640, supplemented with 10% FCS, L-Glutamine (2 mM) and penicillin/streptomycin (100 U/ml). MoDCs were stimulated with 1 µM α2-3 sialic acid or control dendrimer for 5 hours at 37°C, 5% CO_2_. LPS (Lipopolysaccharide from *E. coli* 0111:B4, Sigma-Aldrich) was added at 10 ng/ml where indicated. The total RNA was extracted from the cells using Trizol, according to the manufacturers’ protocol. The quantity and purity were tested using the Nanodrop spectrophotometer (Nanodrop Technologies, Wilmington, USA). Two micrograms of RNA was used for mRNA library preparation with the Illumina^®^ TruSeq^®^ Stranded mRNA sample preparation kit, according to the manufacturers’ protocol, and cDNA/library quality was tested using a 2100 Bioanalyzer (7500 DNA chip, Agilent). RNA sequencing was performed with a single read type of 50 bps on the Illumina HiSeq2500 System (Tumor Genome Analysis Core, Cancer Center Amsterdam, Amsterdam UMC – location VUmc) using standard Illumina protocols.

### Data alignment and differentially expressed genes analysis

Sickle was used to quality trim the RNA sequencing reads before quality checking with FASTQC [26, 27]. Reads were aligned to the Ensemble human genome GRCh38.p10 (release 90) using Spliced Transcripts Alignment to a Reference (STAR 2.5.4a) and subsequent Sequence Alignment Map (SAM) files were created [28, 29]. FeatureCounts (R package Subread 1.26.1, R 3.4.0) was used to quantify aligned reads, excluding multimapping and multi-overlap reads [30, 31]. Library size adjustment and trimmed mean of M-values normalization and subsequent data analysis was performed with R package edgeR (version 3.18.1) [32, 33]. Visualization of sample distribution between α2-3 sialic acid and control dendrimers with or without LPS using Multidimensional scaling (MDS) plots revealed a high degree of donor variation, as well as clustering on LPS. Therefore, a generalized linear model (GLM) was created to identify the significant differentially expressed genes (DEGs) (Likelihood ratio test with Benjamini-Hochberg correction, false discovery rate [FDR] < 0.05). The GLM factored in: donor, LPS, treatment and interaction effects, selected with a forward selection method that gave the lowest dispersion for the model.

### Gene Ontology term enrichment and pathway analysis

Significant DEGs between α2-3 sialic acid and control dendrimer-treated moDCs in combination with LPS were used for Gene Ontology (GO) term enrichment analysis via Cytoscape v3.7.1 in combination with the ClueGO plugin (v2.5.4) [34, 35]. Significantly enriched GO terms (Benjamini-Hochberg corrected FDR) were visualized and described.

### Cytokine secretion by moDCs

Day 5 moDCs (5·10^5^ cells) were stimulated for 24 hours with the α2-3sia or control dendrimers in presence of 10 ng/ml LPS. IL-6, IL-10, IL-12p70, (eBioscience), IL-12p40 (Biosource), IL-23 (Invitrogen), IL-27 (R&D systems), and TNF-α (Invitrogen) secretion was measured in the supernatant using a standard ELISA according to the manufacturer’s protocol, and measured by spectrophotometric analysis at 450 nm on the iMark^TM^ Microplate Absorbance Reader (Bio-RAD).

### Siglec-9 phosphorylation array and ELISA

Day 5 moDCs (1·10^5^ cells/well) were plated in a 96-well plate in FSC-free starvation RPMI-1640 medium and incubated for 4 hours at 4°C and subsequently stimulated for approximately 15 minutes with 10 μM α2-3sia or control dendrimer at 37°C. Cells were lysed with lysis buffer 17 (R&D systems) supplemented with EDTA free cOmplete (Merck, 11875800001) and Orthovanadate (New England Biolabs, P0758S). Protein determination was performed with the micro BCA protein assay kit (Thermo scientific) according to the manufacturer’s instructions. The Human phospho-immunoreceptor array (R&D systems) was performed with 50 μg of lysate according to the manufacturer’s protocol. For the Siglec-9 specific phosphorylation ELISA, NUNC maxisorb plates (RosKilde) were coated overnight at room temperature with 10 μg/ml anti-human Siglec-9 antibody (R&D systems, MAB-1139). Plates were blocked with 0.1% BSA (Merck) in PBS (Braun) for 1 hour at 37°C and subsequently incubated overnight at 4°C with 50 μg of protein lysates in PBS with 1 mM Orthovanadate. After washing with 0.05% Tween in PBS and 0.5 mM Orthovanadate, samples were incubated with anti-pTyr-HRP (R&D systems) for 1 hour at room temperature. Binding was visualized using 3,3′,5,5′-tetramethylbenzidine (TMB) substrate (Sigma-Aldrich) followed by measurement at 450 nm on the iMark^TM^ Microplate Absorbance Reader (Bio-RAD).

### Siglec-Fc binding ELISA

NUNC maxisorb plates (RosKilde) were coated overnight at room temperature with 1 µg/ml α2-3sia or control dendrimers. Plates were blocked with carbo-free blocking buffer (Vector, SP5040) diluted 1:10 with HBSS (Invitrogen) for 1 hour at room temperature and subsequently incubated with 5 μg/ml Siglec-Fc chimeras in combination with goat anti-human or goat anti-mouse Fc-PO antibody (Jackson, 109-036-098 and 115-036-071). Binding was visualized using 3,3′,5,5′-tetramethylbenzidine (TMB) substrate (Sigma-Aldrich) followed by measurement at 450 nm. The following Siglec-Fcs were used: Siglec-1-mFc (R&D systems, 5610-SL), Siglec-3-hFc (R&D systems, 1137-SL), Siglec-7-hFc (R&D systems, 1138-SL), Siglec-9-hFc (R&D systems, 1139-SL), and Siglec-10-hFc (R&D systems, 2130-SL).

### Metabolic extracellular flux assay

Day 5 moDCs (2·× 10^5^ cells/well) were stimulated in a 96-well round bottom plate for 24 hours with the α2-3sia or control dendrimers in presence of 10 ng/ml LPS in RPMI medium supplemented with 10% FCS, 100 U/ml pencillin and streptomycin, and 2 mM glutamine. Cells were harvested and reseeded at 8·× 10^4^ cells/well (five to six replicates) in a Seahorse XF96 Cell culture microplate (Agilent Technologies, 101085-004) that was coated with poly-D-Lysine (Sigma). After 3 hours incubation at 37°C, 5% CO_2_, the medium was replaced with a Seahorse XF base medium (Agilent Technologies, 103335-100) with 2 mM L-glutamine (Sigma-Aldrich, G8540), followed by a short spin at 500 rpm and maximum 1 hour incubation at 37°C in a non-CO_2_ incubator. ECAR and OCR were measured on the Seahorse XF96 Flux Analyzer (Agilent Technologies) in response to the following injections (final concentrations): 10 mM Glucose (G7021), 1.5 μM Oligomycin (O4876), combination of 2 μM Trifluoromethoxy carbonylcyanide phenylhydrazone (FCCP, C2920) and 1 mM Pyruvate (Lonza), and a combination of 0.5 μM Rotenone (R8875) and 0.5 μM Antimycin A (A8674, all from Sigma-Aldrich).

Data were normalized using the protein content of each well, analyzed with the micro-BCA protein assay kit (Thermo scientific) according to the manufacturer’s instructions. Changes in OCR upon the injections were used to calculate basal respiration, maximum respiration, spare respiratory capacity and ATP-production coupled respiration, utilizing the Seahorse Analytics Web-Application [36]. Changes in ECAR were used to calculate basal glycolysis and glycolytic capacity.

### T cell differentiation and regulatory T cell assays

Day 5 moDCs (2 ×·10^5^) were seeded in sterile 48-well plate and incubated at 37°C, 5% CO_2_ for 2 days with 10 µM α2-3sia dendrimers, control dendrimers or 1000 U/ml IFNγ (Becton Dickinson) (positive control for T_H_1 differentiation), all in the presence of 10 ng/ml LPS. The moDCs were harvested, washed (to remove residual α2-3sia dendrimers) and reseeded in a sterile 96-well flat bottom plate at a concentration of 1 ×·10^4^ cells/well. Naive CD4^+^ T cells were isolated from a healthy donor buffy coat (Sanquin Bloodsupply) by MACS isolation with the naive CD4^+^ T cell isolation kit II (MACS Miltenyi Biotec). The allogenic naive CD4^+^ T cells were added to the pre-incubated moDCs in a 1:10 ratio and incubated for 13 days at 37°C, 5% CO_2_. Recombinant IL-2 (200 IU/ml, Proleukin) was added every 2 days. Restimulation of T cells was performed with Phorbol 12-myristate 13-acetate (PMA; 30 μg/ml)/ionomycin (1 μg/ml; Sigma-Aldrich) in the presence of Brefeldin A (5 μg/ml; Sigma-Aldrich) for 5 hours at 37°C, 5% CO_2_. T helper 1 cell differentiation was evaluated by intracellular cytokine staining using anti-IFNγ (clone 25723.11, FITC-labeled, BD Bioscience) and measured by flow cytometry (LSRFortessa X-20, BD Bioscience). For the cytokine secretion assay, the T cells were restimulated with anti-CD3/anti-CD28 beads (Thermo Fisher), whereafter supernatant was collected. IL-4 (Bioscource), IL-10 (eBioscience), IL-13 (Invitrogen), TGFβ (Invitrogen), TNFα (Invitrogen), and IFNγ (eBioscience) were measured by cytokine ELISA according to the manufacturers’ protocol.

To assess regulatory T cell induction, naive CD4^+^ T cells were CSFE (carboxyfluorescein diacetate succinimidyl ester, 0.5 µM; Biolegend) labeled according to the manufacturers’ protocol. CSFE-labeled naive CD4^+^ T cells were subsequently added to the α2-3sia pre-incubated and washed moDCs in a 1:10 ratio for 5 days at 37°C, 5% CO_2_. Supernatant was harvested for cytokine determination by ELISA and T cells were investigated using flow cytometric analysis. Samples were stained with CD4-PE (clone RPA-T4, BD Bioscience), CD25-APC (clone MEM-181, Immunotools), CD127-PE-Cy7 (clone A019D5, Biolegend), FOXP3-V450 (intracellular, clone 259D/C7, BD Bioscience) and fixable viability dye efluor 780 (Invitrogen) and subsequently measured with flow cytometry (LSRFortessa X-20, BD Bioscience) and analyzed with FlowJo (v10).

### Statistics

All data were tested for normality with the Shapiro-Wilk normality test. In case of normal distributed data, groups were compared with a one-way analysis of variance followed by a Dunnett’s multiple comparison test. Groups with non-normal distributed data were compared with a Kruskal Wallis test followed by a Sidak’s posthoc. The Wilcoxon signed rank test was used to compare means with the reference value. The Students *t*-test or Mann–Whitney *U*-test was used to compare independent samples. All tests were performed using Graphpad PRISM version 7. *P*-values ≤ 0.05 were considered significant.

## Results

### α2-3 sialic acid dendrimer binds Siglecs and induce Siglec-9 phosphorylation in moDCs

**Figure 1.**
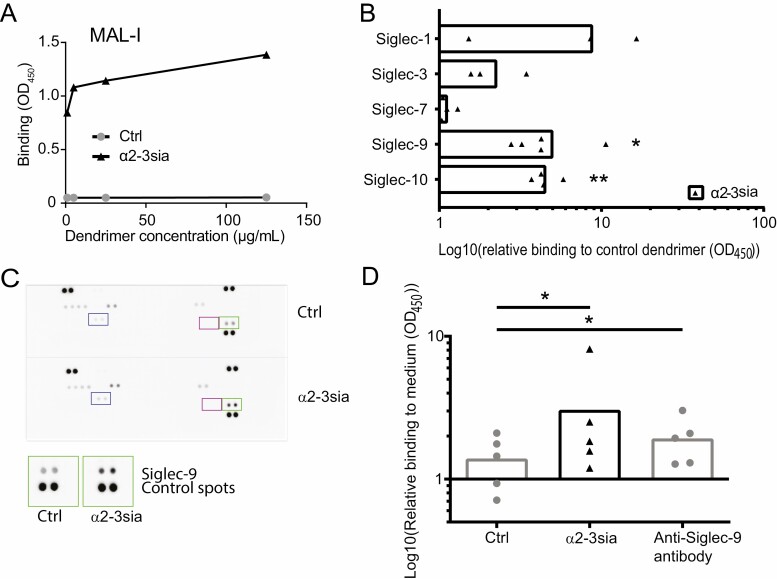
α2-3 sialic acid dendrimers interact with Siglecs and induce Siglec-9 phosphorylation in moDCs (A) Concentration-dependent binding of α2-3sia dendrimers to *Maackia Amurensis* Lectin-1 (MAL-1) was detected in an ELISA assay, while the control dendrimer did not bind. One representative experiment out of three repeats is shown. (B) α2-3 sialic acid dendrimers interact with Siglec-1-Fc and ITIM-expressing Siglec-3-Fc, Siglec-9-Fc (significant), and Siglec-10-Fc (significant), whereas no binding to Siglec-7-Fc was detected with ELISA. All data are shown relative to the open-galactose control dendrimer. One sample *t*-test to compare median to ‘1’, was used to test for significant increase of the binding, *n* = 3–5. (C) Tyrosine phosphorylation array revealed tyrosine phosphorylation specific for Siglec-9 (green squares) in moDCs after α2-3sia dendrimer stimulation (*t* = 15 minutes) compared with control dendrimer. Phosphorylation of Siglec-3 (Purple squares) or Siglec-10 (Blue squares) was not altered in moDCs stimulated with α2-3sia dendrimers compared to control dendrimers. D) Siglec-9-specific phosphorylation in moDCs is significantly increased after 10–15 minutes of α2-3sia dendrimer binding compared with control dendrimer, and similar or higher than the anti-Siglec-9 antibody that serves as a positive control. Difference between the groups was tested with a Friedman’s test followed by a Dunn’s multiple comparison test. * = *P* ≤ 0.05, ** = *P* < 0.01.

To investigate the immunomodulatory effect of sialic acid–Siglec interactions in moDCs, we set out to generate a multivalent α2-3 sialic acid (α2-3sia) dendrimer. α2-3 linked sialic acid was conjugated to a dendrimeric core (G2) through reductive amination for multivalent ligand presentation [37]. The tri-saccharide 3′-sialyl-N-acetyllactosamine was used for coupling, creating α2-3 sia-galactose, as the Siglec binding structure. A galactose monosaccharide was conjugated in a similar fashion, and served as a C_6_H_12_O_5_ (open galactose)-dendrimer control. Plant lectin binding assays were performed using α2-3sia specific *Maackia Amurensis* Lectin I (MAL-I) to validate the α2-3sia presence on the dendrimeric core. Increased binding of MAL-I was measured with increased concentrations of the α2-3sia dendrimer (Fig. 1A). To determine the potential of α2-3sia to bind Siglecs in an ELISA binding assay, chimeric constructs were used, consisting of the extracellular domain of a Siglec fused to the Fc portion of a human IgG (Fig. 1B). We focused on Siglec-1,-3, -7, -9, and -10, as these sialic acid binding receptors are expressed by moDCs [10]. Binding of α2-3sia dendrimers was observed to Siglec-1 and to three ITIM containing Siglecs, where a low, but not significant binding was observed to Siglec-3 and a significant binding to both Siglec -9 and Siglec-10. Siglec-7-Fc was unable to recognize α2-3sia dendrimers. To determine whether the α2-3sia dendrimer induces phosphorylation of ITIM-bearing Siglecs, a human tyrosine phosphorylation immunoreceptor array was performed. This array indicates a clear phosphorylation of Siglec-9 in moDCs upon binding of α2-3sia dendrimer compared with the control dendrimer (Fig. 1C, green box). No phosphorylation of the Siglec-3 and Siglec-10 was detected in moDCs treated with α2-3sia dendrimer compared with the control dendrimer (Fig. 1C, Blue and purple box, respectively). To validate the phosphorylation of Siglec-9, we determined the Siglec-9-specific phosphorylation in moDCs upon binding α2-3sia dendrimers, control dendrimers or polyclonal Siglec-9 antibody with a phosphorylation ELISA. Significantly increased phosphorylation of Siglec-9 on moDCs was observed after engagement of α2-3sia dendrimers compared to control dendrimers, which was equal or higher than the positive control, anti-Siglec-9 antibody known to induce crosslinking and triggering of Siglec-9 receptor (Fig. 1D).

### α2-3 sialic acid dendrimers affect expression of genes related to metabolism and T cell differentiation in LPS-stimulated moDCs

**Figure 2.**
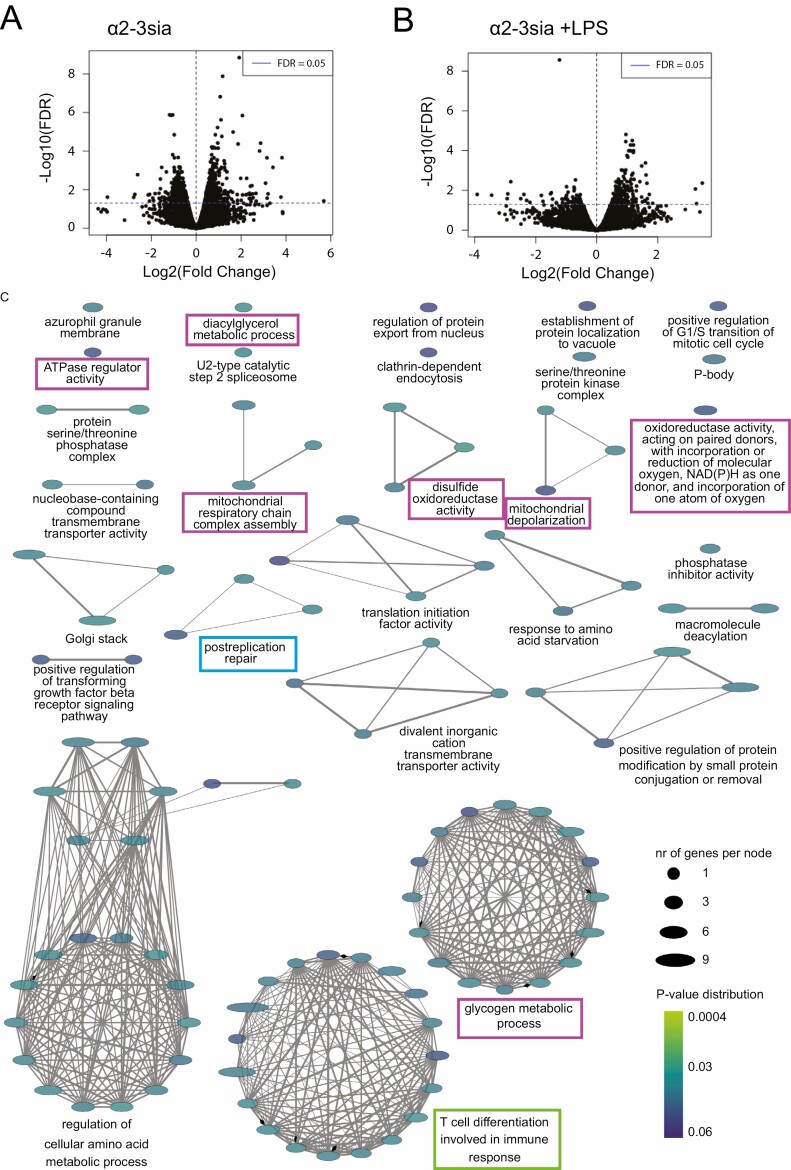
Significantly differentially expressed genes (DEGs) for α2-3sia-treated moDCs. The DEGs for α2-3sia-stimulated moDCs compared to control without (A) and with (B) simultaneous LPS stimulation *t* = 5 hours. The dotted horizontal blue line represents a cut-off for the significant genes (FDR ≤ 0.05). In absence of LPS, the α2-3sia dendrimer stimulated moDCs revealed 1210 significant DEGs compared to control dendrimer stimulated moDCs. In the presence of LPS the α2-3sia stimulated moDCs revealed 309 significant DEGs compared with control dendrimer. (C) The significant (FDR ≤ 0.05) enriched GO terms of α2-3sia and LPS-stimulated moDCs compared to control were visualized in multiple networks. One hundred and seven significant GO terms (represented by each node) were grouped into 29 GO groups (represented by each network). Width of the eclipse describes the number of genes per GO term. The color of the eclipse represents the multiple comparison corrected (Benjamini-Hoghberg correction) p value of the GO term. The blue square represents the most significant GO group ‘postreplication repair’. The purple squares represent GO groups involved in metabolism, and the green square depicts the GO groups ‘T cell differentiation involved in immune response’.

To analyze the early signaling and differentiation events in human moDCs upon α2-3sia binding and their capacity to alter inflammatory LPS-induced signaling, we performed transcriptomic RNA sequencing analysis on moDCs stimulated for 5 hours with α2-3sia or the control dendrimers in the presence or absence of LPS. The analysis of DEGs (FDR ≤ 0.05) revealed 1210 significant DEGs in the α2-3sia compared to the control dendrimer in absence of LPS (Fig. 2A). Because of the low numbers of normalized counts and low gene expression levels, we focused to analyze the effect of α2-3sia dendrimers on LPS-stimulated moDCs. Gene expression in the α2-3sia dendrimer and LPS-stimulated moDCs was more abundant and this resulted in 309 significant DEGs compared with moDCs stimulated with the control dendrimer and LPS (Fig. 2B, Supplementary Table 1). Gene ontology (GO) term enrichment analysis revealed 113 significant GO terms grouped into 28 GO groups, represented by the multiple networks and single nodes (Fig. 2C). The most significant functional GO group was ‘postreplication repair’ (*P* = 0.014, Fig. 2C, blue square, Supplementary Table 2, GO group 19), to which various genes within the ubiquitination pathway were annotated. Multiple GO groups like ‘Glycogen metabolic process’, ‘ATPase regulator activity’, ‘Mitochondrial respiratory chain complex assembly’ and ‘oxidoreductase activity’, suggests changes in cell metabolism (Fig. 2C purple squares, Supplementary Table 2, GO groups 1; 3; 10; 16; 17;18; 26). Notable is the ‘T cell differentiation involved in immune response’ GO group (Fig. 2C green square, Supplementary Table 2, GO group 27), which is one of the biological pathways in the DC maturation process that is affected [38–40].

**Figure 3.**
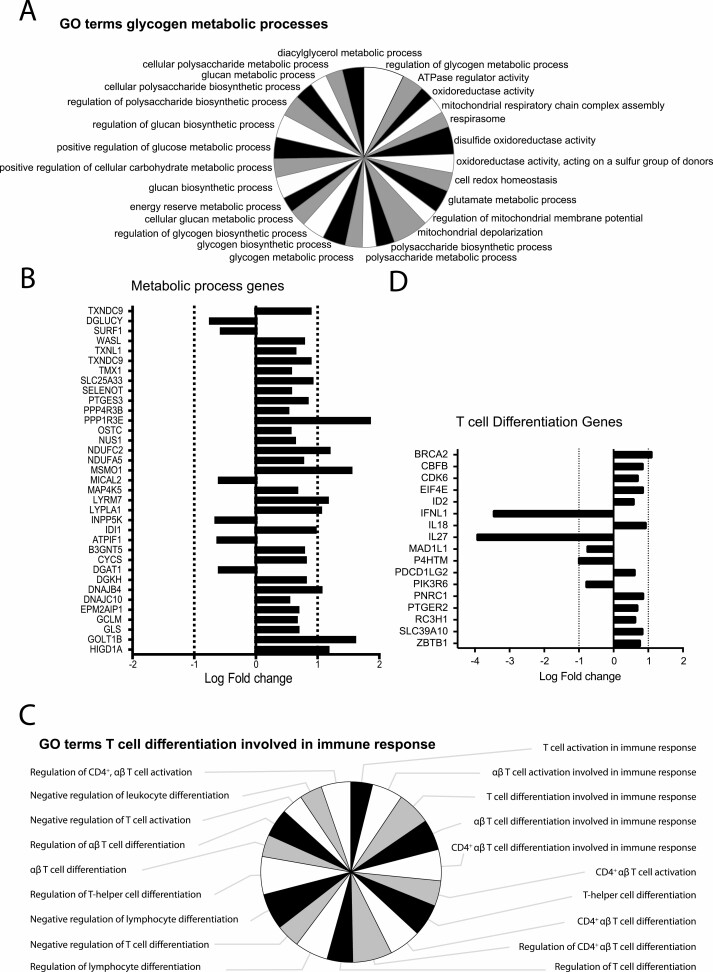
Gene Ontology (GO) term specification for glycogen metabolic processes and T cell differentiation clusters for moDCs responding to α2-3sia and LPS. (A) GO terms represented by GO group ‘Glycogen metabolic processes’, represented as %Genes/Term. (B) The significant DEGs (FDR ≤ 0.05) specific for the GO term ‘Glycogen metabolic processes’ are represented as Log(Fold Change). (C) GO terms represented by GO group ‘T cell differentiation involved in immune response’, represented as %Genes/Term. (D) The significant DEGs (FDR ≤ 0.05) specific for the GO term ‘T cell differentiation involved in immune response’ are represented as Log(Fold Change). The genes *IFNL1* and *IL27* are highly downregulated.

The GO groups about metabolism consist of GO terms on metabolic activity like glycogen metabolic processes, ATPase regulator activity, oxidoreductase activity, regulation of glucose metabolic process, and cellular glucan metabolic process (Fig. 3A, Supplementary Table 2, GO groups 1; 3; 10; 16; 17; 18; 26). Most of the significant DEGs involved in these GO terms were increased as depicted in Fig. 3B. For examples, the *NDUFA5*, *NDUFC2*, and *CYCS* are involved in the TCA cycle, with an increase in the respiratory electron transport system. A change in glutamate metabolism was marked by an increased glutaminase (*GLS*) gene, which catalyzes the glutamine to glutamate reaction. Golgi transport 1B (*GOLT1B*) and protein phosphatase 1 regulatory subunit 3E (*PPP1R3E*) genes were both highly upregulated within these GO groups. *GOLT1B* regulates fatty acid synthesis and *PPP1R3E* is involved in glycogen synthesis [41, 42]. Together this hint toward altered metabolism in moDCs that were stimulated with α2-3sia dendrimers plus LPS.

The ‘T cell differentiation involved in immune response’ GO group included GO terms on leukocyte and lymphocyte differentiation and activation, particularly involving CD4^+^ T cells (Fig. 3C, Supplementary Table 2). Interferon lambda 1 (*IFNL1*) and Interleukin27 (*IL27*) genes, both associated with an anti-viral cytokine response, were highly downregulated within this GO group (Fig. 3D). Both Interferon lambda 1 and IL-27 are known to promote the induction of T helper (T_H_)1 cells during naive T cell differentiation by either stimulating interferon response genes or inflammatory cytokines, like IL-6 [43, 44]. These two altered DEGs therefore imply a role for α2-3sia dendrimers in diverting T_H_1 differentiation.

### α2-3sia dendrimers induce glycolysis in LPS-treated moDCs

**Figure 4.**
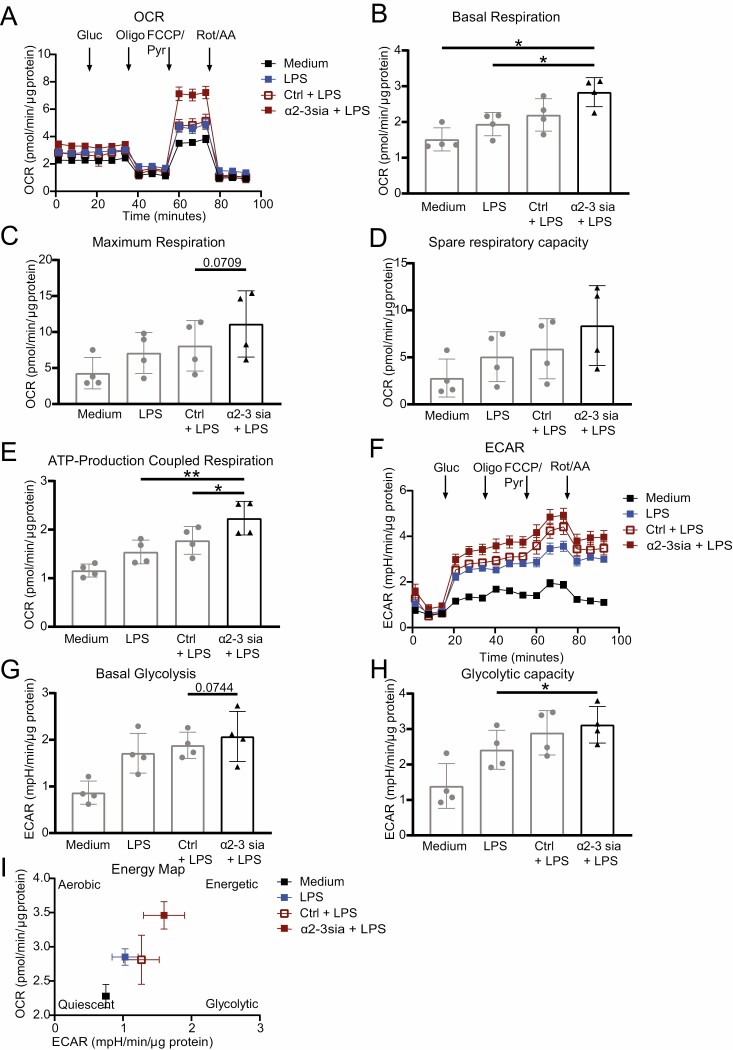
Metabolic extracellular flux analysis reveals an increased oxygen consumption rate (OCR) and basal extracellular acidification rate (ECAR) in α2-3sia dendrimer and LPS stimulated moDCs. (A) OCR of one representative of four independent donors is depicted (*n* = 5–6 replicates ±SEM, normalized based on protein content). From the OCR of all four donors the basal respiration (B), the maximum respiration (C), spare respiratory capacity (D), and ATP-production-coupled respiration (E) were calculated and depicted as mean ± SD. (F) ECAR of one representative of four independent donors is depicted (*n* = 5–6 replicates ±SEM, normalized based on protein content). From the ECAR of all four donors the basal glycolysis (G) and glycolytic capacity (H) were calculated and shown as mean ±SD. (I) Energy map using ECAR and OCR reveals a more energetic moDC stimulated with α2-3sia dendrimers and LPS compared with LPS only or LPS and control dendrimer stimulated moDCs. Difference between the groups was tested with a paired one-way ANOVA followed by a Holm Sidak multiple comparison test. * = *P* ≤ 0.05, ** = *P* < 0.01.

To further assess the effect of α2-3sia dendrimers on cellular metabolism in LPS-treated moDCs, we performed metabolic extracellular flux (Seahorse XF) analysis. Overnight stimulation of the moDCs with α2-3sia dendrimers + LPS resulted in an enhanced OCR compared with the LPS only or the control dendrimer + LPS stimulated moDCs (Fig. 4A). Notably, a significant increase in basal respiration and ATP-production-coupled respiration was observed for the α2-3sia dendrimers+LPS, while only a slight non-significant increase in maximum respiration and spare respiratory capacity was observed for the α2-3sia dendrimers+LPS stimulated moDCs compared to the LPS only and/or the control dendrimer+LPS stimulated moDCs (Fig. 4B–E). Interestingly, the ECAR was also increased in moDCs stimulated with α2-3sia dendrimers + LPS (Fig. 4F), as evidenced by both an increased basal glycolysis and glycolytic capacity (Fig. 4G and H). Mapping the OCR and ECAR in a Seahorse XF Cell energy phenotype profile revealed that moDCs stimulated with α2-3sia dendrimers + LPS have a more energetic phenotype in comparison to the other moDCs (Fig. 4I). Overall, these findings demonstrate that targeting Siglec-9 on moDCs with an α2-3sia dendrimer increases the energy metabolism of the moDCs.

### α2-3sia dendrimers suppress pro- and induce anti-inflammatory cytokines in LPS-treated moDCs

**Figure 5.**
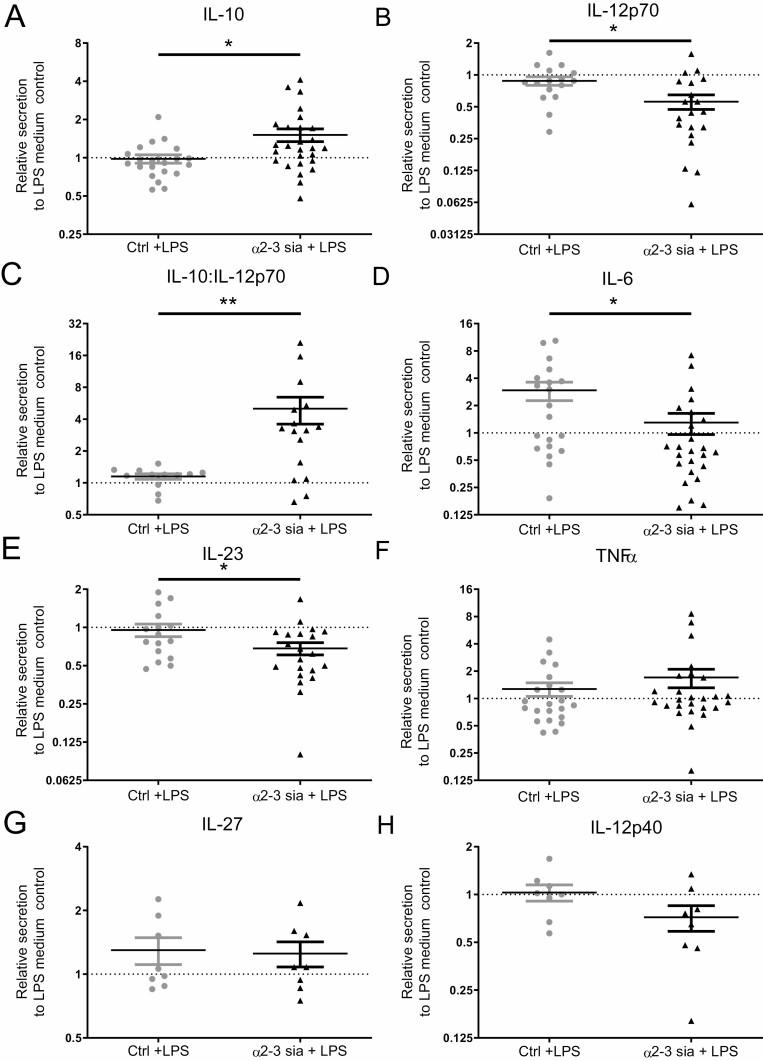
α2-3 sialic acid dendrimers induce an anti-inflammatory DC cytokine secretion profile. (A) Monocyte-derived DCs stimulated overnight with α2-3sia dendrimers showed a significant increase in IL-10 (B), a significant decrease in IL-12p70 and (C) a significant increase in IL-10:IL-12p70 ratio. (D/E) IL-6 and IL-23 secretion by moDCs with α2-3sia dendrimers was significant decreased. (F/G) TNFα and IL-27 were not significantly affected by α2-3sia stimulation. (H) IL-12p40 showed a trend toward decreased secretion by moDCs stimulated with α2-3sia dendrimers. Data is presented relative to the LPS-stimulated medium control, n ≥ 8, ±SD. Range IL-6 1511-12600 pg/ml; IL-10 200-7609 pg/ml; IL-12p40 60-2164 ng/ml; IL-12p70 200-42622pg/ml; IL-23 28-2857 pg/ml; IL-27 690-6002 pg/ml; TNFα 455-33,009 pg/ml. Mann–Whitney *U* test (non-parametric) was used to compare α2-3sia dendrimers with control dendrimers, * = *P* ≤ 0.05.

Next to the affected metabolic pathways, the identified DEGs also pointed toward an alteration in T cell differentiation. Therefore, we investigated whether α2-3sia dendrimers affected the secretion of inflammatory and anti-inflammatory cytokines by moDCs. We quantified the cytokines IL-6, IL-10, IL-12p40, IL-12p70, IL-23, IL-27, and TNF-α in the supernatant of moDCs stimulated overnight with the α2-3sia dendrimers in presence of LPS. All cytokines are depicted in Fig. 5 relative to the LPS only moDC control. moDCs stimulated with α2-3sia dendrimers in the presence of LPS led to a significant increase in IL-10 secretion and a significant decrease in IL-12p70, the active form of IL-12, compared to the control dendrimer (Fig. 5A and B). IL-12 is capable of imposing a negative feedback loop on IL-10 transcription, and IL-10 is likewise able to suppress IL-12 transcription [45]. Therefore, the IL-10:IL-12p70 ratio was plotted, which showed a significant increase after moDC stimulation with α2-3sia dendrimers (Fig. 5C). Furthermore, a significant decrease in IL-6 and IL-23 secretion upon moDC stimulation with α2-3sia dendrimers was detected (Fig. 5D and E). No changes in TNFα and IL-27 secretion were detected (Fig. 5F and G), while a slight trend was visible for decreased IL-12p40 secretion by α2-3sia dendrimer-stimulated moDCs (Fig. 5H). The changes in cytokine profile in the moDCs stimulated with α2-3sia dendrimers are indicative of a more tolerogenic state of the moDCs and hint toward a regulatory T cell differentiation program.

### α2-3sia dendrimer and LPS exposed moDCs display decreased skewing of T_H_1 cells and promote regulatory T cell differentiation

**Figure 6.**
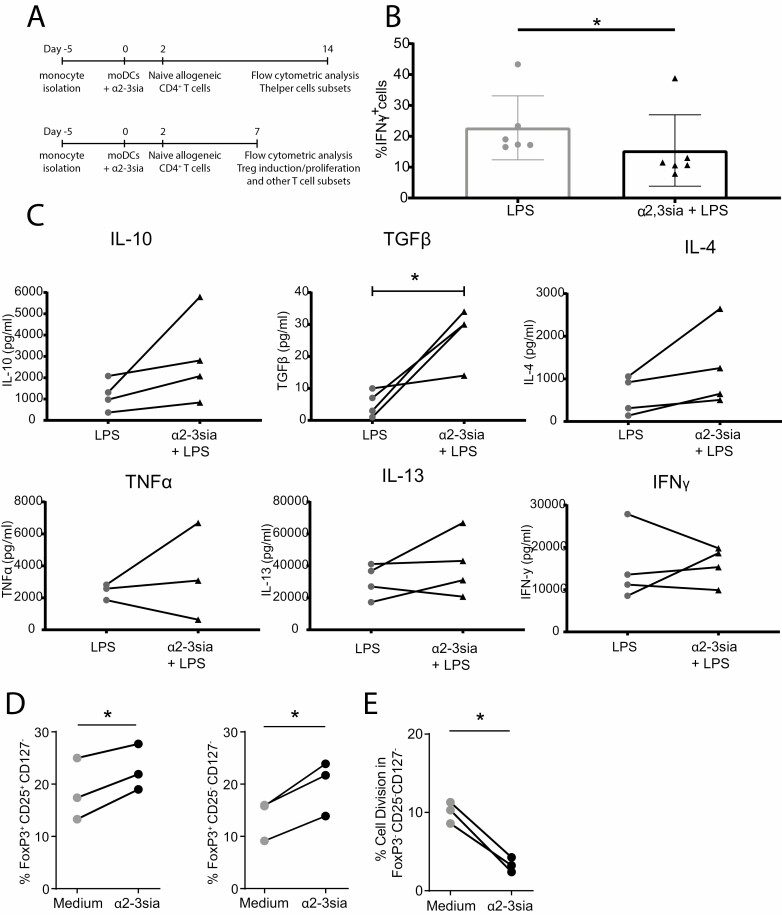
MoDCs stimulated with α2-3sia dendrimers induce tolerogenic T cell differentiation. (A) The workflow for the T helper differentiation assay is represented by the upper time line (Figure 6B, C). The workflow for the regulatory T cell induction and proliferation assay (Figure 6D, E) is represented by the lower time line. (B) Differentiation of naive CD4^+^ T cells was performed upon co-culture with LPS matured moDCs, and α2-3sia and LPS-stimulated moDCs. Flow cytometric analysis of the naive CD4^+^ T cells skewed toward T_H_1 (IFNγ ^+^ cells) demonstrate that stimulation with α2-3sia and LPS resulted in reduced T_H_1 skewing. (C) Cytokine secretion by CD4^+^ T cells was quantified in an ELISA assay after co-culture with LPS-matured moDCs stimulated with α2-3sia dendrimers. Stimulation with α2-3sia dendrimers resulted in a significant increase in TGFβ, and a trend was seen toward increased IL-10 production compared to the LPS control. (D) The induction of regulatory T cells by moDCs treated with α2-3sia dendrimers was measured by flow cytometry. A significant increase of regulatory T cell populations (FoxP3^+^CD25^+^CD127^-^ and FoxP3^+^CD25^-^CD127^-^) was observed compared to the medium control. (E) The proliferation of CD4^+^ T cells by moDCs treated with α2-3sia was quantified by CSFE staining and measured by flow cytometry. A significant decrease in proliferation of the FoxP3^-^CD25^-^CD127^-^ effector T cell population was measured. *N* ≥ 3; Groups were compared with a paired *t*-test or a Wilcoxon matched-pairs signed rank test depending on normality of the data. * = *P* ≤ 0.05.

To explore whether binding of α2-3sia instructs moDCs for altered T helper differentiation, we analyzed the potential of moDCs treated with α2-3sia dendrimers and LPS to induce naive CD4^+^ T cell differentiation. The T_H_1 status was subsequently measured by flow cytometry for IFNγ production by CD4^+^ T cells (Fig. 6A, upper time line). MoDCs treated with α2-3sia dendrimers in presence of LPS showed a significant reduction in their capacity to differentiate naive CD4^+^ T cells into IFNγ ^+^ T_H_1 cells compared to the LPS-stimulated moDCs (Fig. 6B). We also restimulated the CD4^+^ T cells with anti-CD3 and anti-CD28 beads and quantified the secreted cytokines. The T cells from the co-cultures with α2-3sia dendrimer-stimulated moDC demonstrated an increased secretion of IL-10 and TGFβ in most of the donors, compared to the LPS control (Fig. 6C). Secretion of IL-4, IL-13, IFNγ, and TNFα remained unaffected (Fig. 6C). These findings indicate that recognition of α2-3sia dendrimers reprogram the LPS-treated moDCs to skew naive CD4^+^ T cell differentiation toward secretion of anti-inflammatory cytokines, such as IL-10 and TGFβ, indicative of a regulatory T cell phenotype [46].

To confirm naive T cell skewing toward a more regulatory phenotype, moDCs were co-cultured with naive T cells for 7 days (Fig. 6A lower time line), whereafter the expression of FoxP3 and the number of proliferating cells was measured (Fig. 6D). α2-3sia dendrimer-treated moDCs significantly induced naive T cell differentiation toward FoxP3^+^CD25^+^CD127^-^ and FoxP3^+^CD25^+^CD127^+^ regulatory T cell subsets, compared to stimulation with the medium and/or dendrimer control (Fig. 6D and Supplementary Fig. 1A). In combination with LPS, the skewing toward regulatory T cells showed remains similar between the groups (Supplementary Fig. 1B). Moreover, the proliferation of the FoxP3^-^CD25^-^CD127^-^ effector T cell population was significantly diminished after the addition of α2-3sia dendrimers (Fig. 6E and Supplementary Fig. 1C). The overall proliferation of FoxP3^-^CD25^-^CD127^-^ effector T cell population after α2-3sia dendrimer and LPS stimulation was very low (Supplementary Fig. 1D). These findings demonstrate that α2-3sia binding to moDCs, known to phosphorylate Siglec-9 on moDCs (Fig. 1), can convert an LPS-induced T_H_1 differentiation program toward an anti-inflammatory state, and α2-3sia binding to moDCs increases the induction of regulatory T cells, while reducing T_H_1 differentiation and effector T cell proliferation.

## Discussion

In healthy individuals, the induction of immunity and tolerance is strictly balanced to maintain homeostasis. Loss or a break in tolerance often occurs in individuals with allergies or autoimmune diseases, tipping the homeostatic balance toward inflammation [47]. This study shows that α2-3 sialic acid sensing by the Siglec-9 receptor on DCs is key for the induction of a tolerizing pathway in moDCs.

With a human phospho-immunoreceptor array we could only detect phosphorylation of the ITIM of Siglec-9 upon α2-3sia dendrimer binding by human moDCs and not of the other ITIM-bearing Siglec receptors. However, this does not exclude a role for Siglec-1 in binding the α2-3sia dendrimer. Nevertheless, Siglec-1 does not carry intracellular signaling motifs and is lowly expressed on moDCs [10]. We therefore do not anticipate a major role for this Siglec-1 receptor in the tolerizing reprogramming of the moDCs.

Transcriptomic data analysis of α2-3sia and LPS treated moDCs revealed a significant upregulation in genes involved in metabolic processes. The metabolic extracellular flux analysis confirmed this with an overall increase in OCR and ECAR of moDCs stimulated with α2-3sia and LPS. These data are in coherence with metabolic data for other tolerizing agents, like Vitamin D3, for which an increased OXPHOS and glycolysis was described [25, 48]. Further research is needed to compare the impact of different tolerizing agents and their combinations on the energy status and tolerizing capacity of moDCs.

The suppression of inflammatory cytokine secretion profiles imposed on moDCs by α2-3sia were mainly observed in the presence of an inflammatory signal, such as LPS triggering of TLR4. Transcriptomic data analysis of α2-3sia and LPS treated moDCs revealed a significant downregulation of *IFNL* and *IL27* genes. IFN-λ is involved in the induction of interferon response genes and promotes the induction of T_H_1 cells during naive T cell differentiation [43]. IL-27 signaling in DCs enhances production of pro-inflammatory cytokines, like IL-6, and promotes *in-vivo* DC-mediated T_H_1 differentiation [49, 50]. IL-27 is also critical for the function of T follicular helpers, which are localized in the B cell follicles, where they promote B cell immunoglobulin class switching and production [51]. Although, the *IL27* gene is significantly downregulated, the IL-27 cytokine secretion by moDCs is not changed upon binding of α2-3sia and LPS. This could be due to the fact that the IL-27 cytokine consists of two subunits IL-27p28 and EBI3, that are under steady state conditions produced at low levels. However, after LPS stimulation the IL-27p28 subunits show a 2000-fold increase, while the EBI3 remains constant [52]. A reduction of the IL-27p28 subunit encoded by the *IL27* gene, as we showed in Fig. 3D does not necessarily leads to IL-27 cytokine reduction, although it may influence the IL-12 cytokines. We could validate that α2-3sia stimulation of no LPS or LPS-triggered moDCs indeed resulted in decreased secretion of inflammatory cytokines like IL-12 and a decreased naive T cell skewing toward T_H_1 cells, increased FoxP3^+^ regulatory T cell subsets, an increased secretion of IL-10 and TGFβ by T cells and a decreased proliferation of effector T cell populations. This indicates that sialic acids act as SAMPs and may play a crucial role in the resolution phase of inflammation.

The increase in FoxP3^+^ regulatory T cell subsets after reprogramming moDCs with α2-3sia is coherent with murine studies involving vaccination with α2-3 sialylated OVA antigens. The induction of antigen-specific regulatory T cells was accompanied by a reduction of antigen-specific CD4^+^ and CD8^+^ effector T cells [11]. These data imply a role for α2-3sia modification of antigen in the DC-mediated regulatory T cell skewing, further investigations are recommended to confirm the regulatory T cells differentiation.

*Ex-vivo* DC-based vaccination therapies or *in-vivo* DC targeting strategies that restore tolerance against any given antigen would benefit from using the sialic acid-Siglec axis to achieve this goal. Sialylation of antigens (proteins or peptides) is feasible and has been demonstrated to result in the induction of Tregs when antigens, such as OVA or pollen allergen Phlph, were sialylated [11, 53]. In these studies, targeting Siglecs on DCs lead to the induction of antigen-specific T cell tolerance and inhibition of effector cells. Subcutaneous immunotherapy with sialylated Phlph showed reduced ear swelling and eosinophilic influx in the lung, in an allergic asthma mouse model [53], alleviating allergic asthma.

So far, dexamethasone, IL-10, and vitamin D3 have all been described to induce *in*-*vitro* tolerogenic DCs with a strong potency to control inflammation in disease [54]. These tolerogenic moDCs demonstrate decreased production of IL-6 and IL-12, induced IL-10 producing regulatory T cells, and inhibited effector T cell proliferation [55]. Furthermore, moDCs treated with dexamethasone and vitamin D3 have decreased expression of MHC-I, -II, and co-stimulatory molecules [56, 57]. Due to the decreased MHC expression, these tolerogenic DCs are weak antigen-presenting cells, and in need of a secondary boost by adjuvants (e.g. LPS) [58]. Although decreased IL-12 production by α2-3sia-stimulated moDCs was measured, decreased expression of the MHC-I and -II and co-stimulatory molecules was not (data not shown). The tolerogenic program imposed by the sialic acid on the DCs is therefore different from that of dexamethasone or vitamin D3-induced tolerance. Dexamethasone exerts its tolerogenic capacity only through regulation of the NF-κB signaling cascade [7, 59, 60], whereas the α2-3sia-induced tolerance works through the Siglec ITIM-mediated signaling, the association of SHP-phosphatases and JAK/STAT signaling, and has the strength to modulate TLR inflammatory signaling [61]. We here investigated the tolerance a2-3sia imposes on bacterial LPS induced TLR4 triggering and it will be interesting to address whether a2-3sia also antagonize other TLR triggers, for instance, those responding to viruses.

Nonetheless, compared to dexamethasone or vitamin D3 treated DCs, both known to induce tolerance independent of antigen specificity [54], tolerance induction through α2-3sia-treatment of moDCs shows more potential for exploitation in antigen-specific vaccination strategies. Sialylation of autoantigens or allergens, therefore, has great potential as an immunotherapeutic for the treatment of autoimmune diseases and allergies.

## Supplementary material

Supplementary data are available at *Immunotherapy Advances* online.

Supplementary Table 1. Differentially expressed genes (DEGS) of α2-3sia stimulated moDCs compared to control stimulation with or without presence of LPS. (A) DEGS of α2-3sia stimulated moDCs without LPS (B) DEGs of α2-3sia stimulated moDCs with LPS.

Supplementary Table 2. Gene ontology (GO) term enrichment analysis of DEGs from α2-3sia and LPS moDCs. Significant enriched GO terms (FDR ≤ 0.05, Benjamini-Hochberg corrected) were identified via the ClueGO plug-in in Cytoscape. 

Supplementary Figure 1. MoDCs stimulated with α2-3sia dendrimers and LPS induce tolerogenic T cell differentiation. (A) The induction of regulatory T cells by moDCs treated with control or α2-3sia dendrimers was measured by flow cytometry. An increase of regulatory T cell populations (FoxP3^+^CD25^+^CD127^-^ and FoxP3^+^CD25^-^CD127^-^) was observed compared to the medium control and/or control dendrimer. (B) The induction of regulatory T cells by moDCs treated with control or α2-3sia dendrimers in combination with LPS was measured by flow cytometry. (C) The proliferation of CD4^+^ T cells by moDCs treated with control or α2-3sia dendrimers was quantified by CSFE staining and measured by flow cytometry. A significant decrease in proliferation of the FoxP3^-^CD25^-^CD127^-^ effector T cell population was measured compared with the medium control. (D) The proliferation of CD4^+^ T cells by moDCs treated with control or α2-3sia dendrimers in combination with LPS was quantified by CSFE staining and measured by flow cytometry. N ≥ 3; Groups were compared with a paired one-way ANOVA followed by a Holm Sidak multiple comparison test. * = p ≤ 0.05

ltab012_suppl_Supplementary_Materials

## Data Availability

The RNA sequencing data are available at the Sequence Read Archive (SRA) Gene Expression Omnibus through GEO series accession number (GSE168887) The R script used to analyze the RNA sequencing data is available at https://github.com/MolecularCellBiologyImmunology/Sia_dendrimer_transcriptomics[62]. All other data underlying this article are available in the article and in its online supplementary material.

## Abbreviations

α2-3sia: α2-3 linked sialic acid
CSFE: carboxyfluorescein diacetate succinimidyl ester; DCs: Dendritic cells
DEGs: Differentially expressed genes
ECAR: Extracellular acidification rate
FCS: fetal calf serum; FDR: False discovery rate
GLM: Generalized linear model
GO: Gene ontology
IFN: Interferon
IL: Interleukin
ITIMs: Immunoreceptor tyrosine-based inhibitory motifs
LPS: Lipopolysaccharide MAL-I: Maackia Amurensis Lectin I, Map
moDCs: Monocyte-derived dendritic cells
MDS plots: Multidimensional scaling plots
OCR: Oxygen consumption rate
OXPHOS: Oxidative phosphorylation
PRRs: Pathogen recognition receptors
SAM: Sequence Alignment
SAMPs: self-associated molecular patterns
Siglecs: Sialic acid binding immunoglobulin type lectins
TCA: Tricarboxylic acid
TH1: T helper 1
TLRs: Toll-like receptors
Treg: Regulatory T cells
