# Supplementary material for: α2-3 Sialic acid binding and uptake by human monocyte-derived dendritic cells alters metabolism and cytokine release and initiates tolerizing T cell programming
Source: Immunother Adv. 2021 Jun 9;1(1):ltab012. doi: 10.1093/immadv/ltab012 (PMC9327115; doi:10.1093/immadv/ltab012)
Supplement: ltab012_suppl_Supplementary_Materials [file ltab012_suppl_supplementary_materials.zip › ltab012_suppl_Supplementary_Table_S2.docx]

**Supplementary Table 2 | Gene ontology (GO) term enrichment analysis of DEGs from α2-3sia and LPS moDCs.** Significant enriched GO terms (FDR ≤ 0.05, Benjamini-Hochberg corrected) were identified via the ClueGO plug-in in Cytoscape.

| GO Group | GO ID | GO Term | Term PValue Corrected with Benjamini-Hochberg | Group PValue Corrected with Benjamini-Hochberg | Nr. Genes | Associated Genes Found |
| --- | --- | --- | --- | --- | --- | --- |
| 0 | GO:0035577 | azurophil granule membrane | 0.0365 | 0.0305 | 4.00 | [ELK4, NDUFC2, RAP1B, TMEM30A] |
| 1 | GO:0046339 | diacylglycerol metabolic process | 0.0316 | 0.0347 | 3.00 | [DGAT1, DGKH, OSTC] |
| 2 | GO:0046825 | regulation of protein export from nucleus | 0.0485 | 0.0398 | 3.00 | [IFI27, MDM2, XPO1] |
| 3 | GO:0060590 | ATPase regulator activity | 0.0460 | 0.0392 | 3.00 | [ATP5IF1, DNAJB4, DNAJC10] |
| 4 | GO:0071007 | U2-type catalytic step 2 spliceosome | 0.0343 | 0.0280 | 3.00 | [BCAS2, INPP5K, SNRPD2] |
| 5 | GO:0072583 | clathrin-dependent endocytosis | 0.0485 | 0.0398 | 3.00 | [DNM1, SCYL2, WASL] |
| 6 | GO:0072666 | establishment of protein localization to vacuole | 0.0485 | 0.0398 | 3.00 | [SNF8, SNX16, VPS13A] |
| 7 | GO:1900087 | positive regulation of G1/S transition of mitotic cell cycle | 0.0453 | 0.0390 | 3.00 | [CDK10, EIF4E, MDM2] |
| 8 | GO:1902554 | serine/threonine protein kinase complex | 0.0385 | 0.0312 | 5.00 | [C9orf72, CCNG1, CDK6, INPP5K, MMS19] |
| 9 | GO:0000932 | P-body | 0.0385 | 0.0312 | 5.00 | [C9orf72, EIF4E, PNRC2, PSMA2, RC3H1] |
| 10 | GO:0016709 | oxidoreductase activity, acting on paired donors, with incorporation or reduction of molecular oxygen, NAD(P)H as one donor, and incorporation of one atom of oxygen | 0.0465 | 0.0383 | 5.00 | [LYPLA1, MICAL2, MSMO1, NUS1, WASL] |
| 11 | GO:0019212 | phosphatase inhibitor activity | 0.0364 | 0.0253 | 4.00 | [ANP32E, IDI1, PPP1R35, STYX] |
| 12 | GO:1903846 | positive regulation of cellular response to transforming growth factor beta stimulus | 0.0453 | 0.0390 | 3.00 | [EIF4E, GOLT1B, INPP5K] |
| 12 | GO:0030511 | positive regulation of transforming growth factor beta receptor signaling pathway | 0.0453 | 0.0390 | 3.00 | [EIF4E, GOLT1B, INPP5K] |
| 13 | GO:0098732 | macromolecule deacylation | 0.0343 | 0.0232 | 6.00 | [ABHD13, ELK4, LYPLA1, MIER1, MIER3, ZNHIT1] |
| 13 | GO:0035601 | protein deacylation | 0.0357 | 0.0232 | 6.00 | [ABHD13, ELK4, LYPLA1, MIER1, MIER3, ZNHIT1] |
| 14 | GO:0008287 | protein serine/threonine phosphatase complex | 0.0303 | 0.0269 | 4.00 | [CYCS, NCK1, PPP3R1, PPP4R3B] |
| 14 | GO:1903293 | phosphatase complex | 0.0303 | 0.0269 | 4.00 | [CYCS, NCK1, PPP3R1, PPP4R3B] |
| 15 | GO:0015605 | organophosphate ester transmembrane transporter activity | 0.0399 | 0.0472 | 3.00 | [SLC25A24, SLC25A33, TMEM30A] |
| 15 | GO:0015932 | nucleobase-containing compound transmembrane transporter activity | 0.0344 | 0.0472 | 4.00 | [HNRNPA3, SLC25A24, SLC25A33, SLC29A1] |
| 16 | GO:0070469 | respirasome | 0.0369 | 0.0246 | 5.00 | [CYCS, HIGD1A, NDUFA5, NDUFC2, SURF1] |
| 16 | GO:0033108 | mitochondrial respiratory chain complex assembly | 0.0343 | 0.0246 | 5.00 | [LYRM7, NDUFA5, NDUFC2, SLC25A33, SURF1] |
| 16 | GO:0017004 | cytochrome complex assembly | 0.0343 | 0.0246 | 3.00 | [LYRM7, SLC25A33, SURF1] |
| 17 | GO:0016667 | oxidoreductase activity, acting on a sulfur group of donors | 0.0370 | 0.0385 | 4.00 | [DNAJC10, SELENOT, TMX1, TXNL1] |
| 17 | GO:0045454 | cell redox homeostasis | 0.0330 | 0.0385 | 5.00 | [DNAJC10, SELENOT, TMX1, TXNDC9, TXNL1] |
| 17 | GO:0015036 | disulfide oxidoreductase activity | 0.0295 | 0.0385 | 4.00 | [DNAJC10, SELENOT, TMX1, TXNL1] |
| 18 | GO:0051881 | regulation of mitochondrial membrane potential | 0.0476 | 0.0272 | 4.00 | [ATP5IF1, GCLM, GOLT1B, SLC25A33] |
| 18 | GO:0051882 | mitochondrial depolarization | 0.0338 | 0.0272 | 3.00 | [ATP5IF1, GCLM, GOLT1B] |
| 18 | GO:0006536 | glutamate metabolic process | 0.0351 | 0.0272 | 4.00 | [DGLUCY, GCLM, GLS, GOLT1B] |
| 19 | GO:0061631 | ubiquitin conjugating enzyme activity | 0.0346 | 0.0105 | 4.00 | [UBE2A, UBE2D1, UBE2N, UBE2O] |
| 19 | GO:0006301 | postreplication repair | 0.0337 | 0.0105 | 4.00 | [RCHY1, UBE2A, UBE2N, ZBTB1] |
| 19 | GO:0016574 | histone ubiquitination | 0.0452 | 0.0105 | 5.00 | [RYBP, SUZ12, UBE2A, UBE2N, WDR70] |
| 20 | GO:1990928 | response to amino acid starvation | 0.0351 | 0.0207 | 4.00 | [BMT2, LYPLA1, RRAGC, SEH1L] |
| 20 | GO:0034198 | cellular response to amino acid starvation | 0.0364 | 0.0207 | 4.00 | [BMT2, LYPLA1, RRAGC, SEH1L] |
| 20 | GO:0038202 | TORC1 signaling | 0.0413 | 0.0207 | 4.00 | [BMT2, C9orf72, RRAGC, SEH1L] |
| 21 | GO:0005795 | Golgi stack | 0.0326 | 0.0403 | 8.00 | [CLN3, GALNT1, GOLGA8A, GOLT1B, MAN2A1, MOB4, NCBP2, YIPF6] |
| 21 | GO:0031985 | Golgi cisterna | 0.0347 | 0.0403 | 7.00 | [GALNT1, GOLGA8A, GOLT1B, MAN2A1, MOB4, NCBP2, YIPF6] |
| 21 | GO:0000137 | Golgi cis cisterna | 0.0342 | 0.0403 | 3.00 | [GOLGA8A, GOLT1B, NCBP2] |
| 22 | GO:1903322 | positive regulation of protein modification by small protein conjugation or removal | 0.0326 | 0.0261 | 8.00 | [ARRDC3, DCUN1D1, DCUN1D4, NDFIP2, RCHY1, UBE2D1, UBE2N, XIAP] |
| 22 | GO:0051438 | regulation of ubiquitin-protein transferase activity | 0.0453 | 0.0261 | 4.00 | [ARRDC3, DCUN1D1, DCUN1D4, UBE2N] |
| 22 | GO:0031398 | positive regulation of protein ubiquitination | 0.0368 | 0.0261 | 8.00 | [ARRDC3, DCUN1D1, DCUN1D4, NDFIP2, RCHY1, UBE2D1, UBE2N, XIAP] |
| 22 | GO:0051443 | positive regulation of ubiquitin-protein transferase activity | 0.0370 | 0.0261 | 4.00 | [ARRDC3, DCUN1D1, DCUN1D4, UBE2N] |
| 23 | GO:0006829 | zinc ion transport | 0.0346 | 0.0275 | 3.00 | [SLC30A5, SLC30A7, SLC39A10] |
| 23 | GO:0072509 | divalent inorganic cation transmembrane transporter activity | 0.0335 | 0.0275 | 3.00 | [SLC30A5, SLC39A10, SLC41A2] |
| 23 | GO:0006882 | cellular zinc ion homeostasis | 0.0387 | 0.0275 | 3.00 | [SLC30A5, SLC30A7, SLC39A10] |
| 23 | GO:0055069 | zinc ion homeostasis | 0.0417 | 0.0275 | 3.00 | [SLC30A5, SLC30A7, SLC39A10] |
| 24 | GO:0006446 | regulation of translational initiation | 0.0500 | 0.0289 | 4.00 | [EIF4E, INTS6, NCBP2, NCK1] |
| 24 | GO:0002183 | cytoplasmic translational initiation | 0.0371 | 0.0289 | 4.00 | [EIF4E, INTS6, MCTS1, NCK1] |
| 24 | GO:0008135 | translation factor activity, RNA binding | 0.0385 | 0.0289 | 5.00 | [EIF1AX, EIF4E, INTS6, MCTS1, TCEA1] |
| 24 | GO:0003743 | translation initiation factor activity | 0.0340 | 0.0289 | 4.00 | [EIF1AX, EIF4E, INTS6, MCTS1] |
| 25 | GO:0005844 | polysome | 0.0360 | 0.0322 | 5.00 | [EIF4E, NAA50, PSMA1, PSMA2, VBP1] |
| 25 | GO:0031365 | N-terminal protein amino acid modification | 0.0473 | 0.0322 | 3.00 | [EIF4E, NAA15, NAA50] |
| 25 | GO:0006474 | N-terminal protein amino acid acetylation | 0.0334 | 0.0322 | 3.00 | [EIF4E, NAA15, NAA50] |
| 25 | GO:0002220 | innate immune response activating cell surface receptor signaling pathway | 0.0353 | 0.0322 | 6.00 | [EIF4E, NRAS, PSMA1, PSMA2, PSMD12, PSMD5] |
| 25 | GO:0043620 | regulation of DNA-templated transcription in response to stress | 0.0362 | 0.0322 | 7.00 | [EIF4E, HIGD1A, NCK1, PSMA1, PSMA2, PSMD12, PSMD5] |
| 25 | GO:0002223 | stimulatory C-type lectin receptor signaling pathway | 0.0345 | 0.0322 | 6.00 | [EIF4E, NRAS, PSMA1, PSMA2, PSMD12, PSMD5] |
| 25 | GO:0043618 | regulation of transcription from RNA polymerase II promoter in response to stress | 0.0335 | 0.0322 | 7.00 | [EIF4E, HIGD1A, NCK1, PSMA1, PSMA2, PSMD12, PSMD5] |
| 25 | GO:0061418 | regulation of transcription from RNA polymerase II promoter in response to hypoxia | 0.0393 | 0.0322 | 6.00 | [EIF4E, HIGD1A, PSMA1, PSMA2, PSMD12, PSMD5] |
| 26 | GO:0005976 | polysaccharide metabolic process | 0.0342 | 0.0246 | 6.00 | [B3GNT5, EPM2AIP1, IDI1, INPP5K, PPP1R3E, PTGES3] |
| 26 | GO:0006112 | energy reserve metabolic process | 0.0387 | 0.0246 | 5.00 | [EPM2AIP1, IDI1, INPP5K, PPP1R3E, PTGES3] |
| 26 | GO:0044264 | cellular polysaccharide metabolic process | 0.0343 | 0.0246 | 6.00 | [B3GNT5, EPM2AIP1, IDI1, INPP5K, PPP1R3E, PTGES3] |
| 26 | GO:0000271 | polysaccharide biosynthetic process | 0.0340 | 0.0246 | 5.00 | [B3GNT5, EPM2AIP1, INPP5K, PPP1R3E, PTGES3] |
| 26 | GO:0044042 | glucan metabolic process | 0.0343 | 0.0246 | 5.00 | [EPM2AIP1, IDI1, INPP5K, PPP1R3E, PTGES3] |
| 26 | GO:0006073 | cellular glucan metabolic process | 0.0343 | 0.0246 | 5.00 | [EPM2AIP1, IDI1, INPP5K, PPP1R3E, PTGES3] |
| 26 | GO:0010676 | positive regulation of cellular carbohydrate metabolic process | 0.0381 | 0.0246 | 4.00 | [EPM2AIP1, MAP4K5, PPP1R3E, PPP4R3B] |
| 26 | GO:0033692 | cellular polysaccharide biosynthetic process | 0.0363 | 0.0246 | 5.00 | [B3GNT5, EPM2AIP1, INPP5K, PPP1R3E, PTGES3] |
| 26 | GO:0005977 | glycogen metabolic process | 0.0337 | 0.0246 | 5.00 | [EPM2AIP1, IDI1, INPP5K, PPP1R3E, PTGES3] |
| 26 | GO:0010907 | positive regulation of glucose metabolic process | 0.0453 | 0.0246 | 3.00 | [EPM2AIP1, PPP1R3E, PPP4R3B] |
| 26 | GO:0032885 | regulation of polysaccharide biosynthetic process | 0.0485 | 0.0246 | 3.00 | [EPM2AIP1, INPP5K, PPP1R3E] |
| 26 | GO:0009250 | glucan biosynthetic process | 0.0337 | 0.0246 | 4.00 | [EPM2AIP1, INPP5K, PPP1R3E, PTGES3] |
| 26 | GO:0010962 | regulation of glucan biosynthetic process | 0.0386 | 0.0246 | 3.00 | [EPM2AIP1, INPP5K, PPP1R3E] |
| 26 | GO:0070873 | regulation of glycogen metabolic process | 0.0453 | 0.0246 | 3.00 | [EPM2AIP1, INPP5K, PPP1R3E] |
| 26 | GO:0005978 | glycogen biosynthetic process | 0.0337 | 0.0246 | 4.00 | [EPM2AIP1, INPP5K, PPP1R3E, PTGES3] |
| 26 | GO:0005979 | regulation of glycogen biosynthetic process | 0.0386 | 0.0246 | 3.00 | [EPM2AIP1, INPP5K, PPP1R3E] |
| 27 | GO:0045622 | regulation of T-helper cell differentiation | 0.0368 | 0.0286 | 3.00 | [IL18, IL27, RC3H1] |
| 27 | GO:0002294 | CD4-positive, alpha-beta T cell differentiation involved in immune response | 0.0347 | 0.0286 | 4.00 | [IL18, IL27, PTGER2, RC3H1] |
| 27 | GO:0043370 | regulation of CD4-positive, alpha-beta T cell differentiation | 0.0328 | 0.0286 | 4.00 | [CBFB, IL18, IL27, RC3H1] |
| 27 | GO:0042093 | T-helper cell differentiation | 0.0340 | 0.0286 | 4.00 | [IL18, IL27, PTGER2, RC3H1] |
| 27 | GO:0002286 | T cell activation involved in immune response | 0.0462 | 0.0286 | 5.00 | [IFNL1, IL18, IL27, PTGER2, RC3H1] |
| 27 | GO:0002287 | alpha-beta T cell activation involved in immune response | 0.0345 | 0.0286 | 4.00 | [IL18, IL27, PTGER2, RC3H1] |
| 27 | GO:0035710 | CD4-positive, alpha-beta T cell activation | 0.0345 | 0.0286 | 5.00 | [CBFB, IL18, IL27, PTGER2, RC3H1] |
| 27 | GO:0050868 | negative regulation of T cell activation | 0.0363 | 0.0286 | 6.00 | [CBFB, EIF4E, IFNL1, MAD1L1, PDCD1LG2, RC3H1] |
| 27 | GO:1902106 | negative regulation of leukocyte differentiation | 0.0402 | 0.0286 | 5.00 | [CBFB, CDK6, ID2, IFNL1, RC3H1] |
| 27 | GO:0045619 | regulation of lymphocyte differentiation | 0.0395 | 0.0286 | 9.00 | [BRCA2, CBFB, ID2, IFNL1, IL18, IL27, PIK3R6, RC3H1, ZBTB1] |
| 27 | GO:0045620 | negative regulation of lymphocyte differentiation | 0.0351 | 0.0286 | 4.00 | [CBFB, ID2, IFNL1, RC3H1] |
| 27 | GO:2000514 | regulation of CD4-positive, alpha-beta T cell activation | 0.0360 | 0.0286 | 4.00 | [CBFB, IL18, IL27, RC3H1] |
| 27 | GO:0002292 | T cell differentiation involved in immune response | 0.0306 | 0.0286 | 5.00 | [IFNL1, IL18, IL27, PTGER2, RC3H1] |
| 27 | GO:0045580 | regulation of T cell differentiation | 0.0355 | 0.0286 | 8.00 | [BRCA2, CBFB, IFNL1, IL18, IL27, PIK3R6, RC3H1, ZBTB1] |
| 27 | GO:0046632 | alpha-beta T cell differentiation | 0.0452 | 0.0286 | 5.00 | [CBFB, IL18, IL27, PTGER2, RC3H1] |
| 27 | GO:0045581 | negative regulation of T cell differentiation | 0.0453 | 0.0286 | 3.00 | [CBFB, IFNL1, RC3H1] |
| 27 | GO:0002293 | alpha-beta T cell differentiation involved in immune response | 0.0345 | 0.0286 | 4.00 | [IL18, IL27, PTGER2, RC3H1] |
| 27 | GO:0043367 | CD4-positive, alpha-beta T cell differentiation | 0.0329 | 0.0286 | 5.00 | [CBFB, IL18, IL27, PTGER2, RC3H1] |
| 27 | GO:0046637 | regulation of alpha-beta T cell differentiation | 0.0381 | 0.0286 | 4.00 | [CBFB, IL18, IL27, RC3H1] |
| 28 | GO:0005844 | polysome | 0.0360 | 0.0232 | 5.00 | [EIF4E, NAA50, PSMA1, PSMA2, VBP1] |
| 28 | GO:1905368 | peptidase complex | 0.0385 | 0.0232 | 5.00 | [PSMA1, PSMA2, PSMD12, PSMD5, TXNL1] |
| 28 | GO:0000502 | proteasome complex | 0.0321 | 0.0232 | 5.00 | [PSMA1, PSMA2, PSMD12, PSMD5, TXNL1] |
| 28 | GO:0002474 | antigen processing and presentation of peptide antigen via MHC class I | 0.0387 | 0.0232 | 5.00 | [PSMA1, PSMA2, PSMD12, PSMD5, SNAP23] |
| 28 | GO:1905369 | endopeptidase complex | 0.0305 | 0.0232 | 5.00 | [PSMA1, PSMA2, PSMD12, PSMD5, TXNL1] |
| 28 | GO:2000736 | regulation of stem cell differentiation | 0.0353 | 0.0232 | 6.00 | [CBFB, CDK6, PSMA1, PSMA2, PSMD12, PSMD5] |
| 28 | GO:0033238 | regulation of cellular amine metabolic process | 0.0345 | 0.0232 | 5.00 | [AZIN1, PSMA1, PSMA2, PSMD12, PSMD5] |
| 28 | GO:0042590 | antigen processing and presentation of exogenous peptide antigen via MHC class I | 0.0339 | 0.0232 | 5.00 | [PSMA1, PSMA2, PSMD12, PSMD5, SNAP23] |
| 28 | GO:0006521 | regulation of cellular amino acid metabolic process | 0.0305 | 0.0232 | 5.00 | [AZIN1, PSMA1, PSMA2, PSMD12, PSMD5] |
| 28 | GO:0002479 | antigen processing and presentation of exogenous peptide antigen via MHC class I, TAP-dependent | 0.0350 | 0.0232 | 5.00 | [PSMA1, PSMA2, PSMD12, PSMD5, SNAP23] |
| 28 | GO:0070498 | interleukin-1-mediated signaling pathway | 0.0334 | 0.0232 | 6.00 | [IRAK3, PSMA1, PSMA2, PSMD12, PSMD5, UBE2N] |
| 28 | GO:0060218 | hematopoietic stem cell differentiation | 0.0335 | 0.0232 | 6.00 | [CBFB, CDK6, PSMA1, PSMA2, PSMD12, PSMD5] |
| 28 | GO:0002220 | innate immune response activating cell surface receptor signaling pathway | 0.0353 | 0.0232 | 6.00 | [EIF4E, NRAS, PSMA1, PSMA2, PSMD12, PSMD5] |
| 28 | GO:1902036 | regulation of hematopoietic stem cell differentiation | 0.0433 | 0.0232 | 6.00 | [CBFB, CDK6, PSMA1, PSMA2, PSMD12, PSMD5] |
| 28 | GO:0043620 | regulation of DNA-templated transcription in response to stress | 0.0362 | 0.0232 | 7.00 | [EIF4E, HIGD1A, NCK1, PSMA1, PSMA2, PSMD12, PSMD5] |
| 28 | GO:0002223 | stimulatory C-type lectin receptor signaling pathway | 0.0345 | 0.0232 | 6.00 | [EIF4E, NRAS, PSMA1, PSMA2, PSMD12, PSMD5] |
| 28 | GO:0043618 | regulation of transcription from RNA polymerase II promoter in response to stress | 0.0335 | 0.0232 | 7.00 | [EIF4E, HIGD1A, NCK1, PSMA1, PSMA2, PSMD12, PSMD5] |
| 28 | GO:0031145 | anaphase-promoting complex-dependent catabolic process | 0.0359 | 0.0232 | 5.00 | [PSMA1, PSMA2, PSMD12, PSMD5, UBE2D1] |
| 28 | GO:0031146 | SCF-dependent proteasomal ubiquitin-dependent protein catabolic process | 0.0335 | 0.0232 | 6.00 | [CUL5, PSMA1, PSMA2, PSMD12, PSMD5, STYX] |
| 28 | GO:0061418 | regulation of transcription from RNA polymerase II promoter in response to hypoxia | 0.0393 | 0.0232 | 6.00 | [EIF4E, HIGD1A, PSMA1, PSMA2, PSMD12, PSMD5] |
