# Supplementary material for: α2-3 Sialic acid binding and uptake by human monocyte-derived dendritic cells alters metabolism and cytokine release and initiates tolerizing T cell programming
Source: Immunother Adv. 2021 Jun 9;1(1):ltab012. doi: 10.1093/immadv/ltab012 (PMC9327115; doi:10.1093/immadv/ltab012)
Supplement: ltab012_suppl_Supplementary_Materials [file ltab012_suppl_supplementary_materials.zip › ltab012_suppl_Supplementary_Table_S1.docx]

**Supplementary Table 1 | Differentially expressed genes (DEGS) of α2-3sia stimulated moDCs compared to control stimulation** **with or without presence of LPS. (A) DEGS of α2-3sia stimulated moDCs without LPS (B) DEGs of α2-3sia stimulated moDCs with LPS.**

**A)**

| α2,3 genes | logFC | PValue | FDR |
| --- | --- | --- | --- |
| GABARAP | 1.91584469 | 1.15E-13 | 1.44E-09 |
| UBALD2 | 1.17901791 | 2.15E-12 | 1.34E-08 |
| RPS15 | 1.07068432 | 3.69E-11 | 1.54E-07 |
| SPOPL | -1.048215 | 4.27E-10 | 1.33E-06 |
| DNAJB4 | -1.1853503 | 5.32E-10 | 1.33E-06 |
| TWF1 | -1.1156357 | 6.81E-10 | 1.42E-06 |
| MIF | 2.0594717 | 8.14E-10 | 1.45E-06 |
| C19orf24 | 1.10405694 | 1.54E-09 | 2.40E-06 |
| SOCS1 | 0.95436343 | 4.36E-09 | 6.04E-06 |
| ASCL2 | 1.64496461 | 8.29E-09 | 1.03E-05 |
| LPAR6 | -0.9779772 | 1.33E-08 | 1.43E-05 |
| ATP5D | 0.85339515 | 1.37E-08 | 1.43E-05 |
| TMEM160 | 1.14145062 | 1.82E-08 | 1.75E-05 |
| CENPX | 0.95953772 | 4.32E-08 | 3.85E-05 |
| SRXN1 | 2.86870492 | 4.68E-08 | 3.90E-05 |
| C4orf48 | 1.86691594 | 5.57E-08 | 4.35E-05 |
| HMGA1 | 0.69116641 | 8.77E-08 | 6.21E-05 |
| KCTD17 | 0.75512809 | 8.95E-08 | 6.21E-05 |
| MZT2B | 0.84860221 | 1.07E-07 | 7.00E-05 |
| BRI3 | 0.72785659 | 1.16E-07 | 7.14E-05 |
| CHCHD10 | 0.97892167 | 1.20E-07 | 7.14E-05 |
| HLA-DRB9 | 2.82379313 | 1.75E-07 | 9.95E-05 |
| AKAP9 | -1.0468628 | 2.95E-07 | 0.00015612 |
| MFSD10 | 0.68927786 | 3.00E-07 | 0.00015612 |
| KBTBD8 | -0.8014343 | 4.35E-07 | 0.00021718 |
| AL022718.1 | 3.82382614 | 4.63E-07 | 0.00022253 |
| PHPT1 | 0.79204801 | 5.08E-07 | 0.00022551 |
| HES4 | 3.1535744 | 5.14E-07 | 0.00022551 |
| MSMO1 | -0.9714943 | 5.24E-07 | 0.00022551 |
| CLEC4G | 0.68953102 | 6.39E-07 | 0.00026606 |
| CLEC11A | 0.94358021 | 7.22E-07 | 0.00029058 |
| SELENOH | 0.93388965 | 7.61E-07 | 0.00029678 |
| PPP1R35 | 1.24432097 | 8.53E-07 | 0.00032264 |
| HPCAL1 | 0.6371072 | 9.89E-07 | 0.00036312 |
| LIMD2 | 0.67174839 | 1.08E-06 | 0.00038656 |
| SLC39A3 | 0.9105202 | 1.32E-06 | 0.00044922 |
| SSBP4 | 0.7760671 | 1.34E-06 | 0.00044922 |
| VIM-AS1 | 1.27033736 | 1.37E-06 | 0.00044922 |
| TAF10 | 0.73684355 | 1.89E-06 | 0.00060496 |
| FZD2 | 0.84717952 | 2.03E-06 | 0.00063307 |
| SSNA1 | 0.82340332 | 2.23E-06 | 0.00067267 |
| FBXO30 | -0.7669617 | 2.31E-06 | 0.00067267 |
| IDI1 | -0.7304572 | 2.32E-06 | 0.00067267 |
| C20orf24 | 0.77498611 | 2.43E-06 | 0.00069074 |
| SH2B2 | 0.89880613 | 2.52E-06 | 0.00070019 |
| RPS27AP16 | 3.42022889 | 2.65E-06 | 0.00070826 |
| MEX3D | 1.12422617 | 2.78E-06 | 0.00070826 |
| ETFRF1 | -1.5365321 | 2.80E-06 | 0.00070826 |
| SLC30A9 | -0.7178791 | 2.82E-06 | 0.00070826 |
| ACTR6 | -0.9820619 | 2.84E-06 | 0.00070826 |
| MOB2 | 0.80403104 | 2.92E-06 | 0.00070826 |
| AL138963.3 | 1.26164155 | 2.95E-06 | 0.00070826 |
| PDZD8 | -0.7226443 | 3.48E-06 | 0.00081747 |
| FAM173A | 1.10426899 | 3.57E-06 | 0.00081747 |
| SPHK1 | 0.70930125 | 3.60E-06 | 0.00081747 |
| SLC22A18 | 1.02895863 | 4.21E-06 | 0.00093886 |
| PTMS | 0.66632498 | 4.49E-06 | 0.00095895 |
| PSMA6 | 1.28418771 | 4.51E-06 | 0.00095895 |
| RNF181 | 0.62050922 | 4.53E-06 | 0.00095895 |
| SC5D | -0.9681909 | 4.72E-06 | 0.00098197 |
| SCRIB | 0.72203865 | 5.09E-06 | 0.00104151 |
| CFD | 0.97104874 | 5.93E-06 | 0.00119364 |
| VPS13A | -0.9686895 | 6.20E-06 | 0.00121066 |
| MDM2 | -0.6298803 | 6.24E-06 | 0.00121066 |
| AHR | -0.6502536 | 6.34E-06 | 0.00121066 |
| FASTK | 0.74332022 | 6.65E-06 | 0.00121066 |
| MFSD7 | 0.70229239 | 6.66E-06 | 0.00121066 |
| MOSPD1 | -0.8158703 | 6.67E-06 | 0.00121066 |
| ZNF787 | 0.74031919 | 6.73E-06 | 0.00121066 |
| LY6E | 0.65125511 | 6.83E-06 | 0.00121066 |
| LTBP3 | 1.36898943 | 6.94E-06 | 0.00121066 |
| C9orf16 | 0.75400508 | 6.99E-06 | 0.00121066 |
| ZNF570 | -1.3296997 | 7.08E-06 | 0.00121066 |
| C8orf82 | 1.03082793 | 7.85E-06 | 0.00132454 |
| EMC10 | 0.59171183 | 8.19E-06 | 0.00135217 |
| FOPNL | -0.8004655 | 8.23E-06 | 0.00135217 |
| PPP1R14A | 0.8184834 | 8.73E-06 | 0.00141579 |
| COPS2 | -0.7527566 | 9.44E-06 | 0.00151101 |
| ESPNL | 0.66364605 | 9.65E-06 | 0.00152539 |
| PGP | 0.82593121 | 1.04E-05 | 0.00163003 |
| MAD2L1 | -2.6105355 | 1.10E-05 | 0.00168207 |
| ERF | 0.69707487 | 1.10E-05 | 0.00168207 |
| OAF | 0.86108393 | 1.13E-05 | 0.00170036 |
| RBM12B | -0.8259523 | 1.19E-05 | 0.00176456 |
| NDUFA2 | 0.66307812 | 1.24E-05 | 0.00181417 |
| RAB3IL1 | 1.02238356 | 1.28E-05 | 0.00186483 |
| APOE | 0.67531595 | 1.41E-05 | 0.00202492 |
| ACAP3 | 0.63236529 | 1.55E-05 | 0.00219899 |
| ANKRD9 | 0.84724186 | 1.62E-05 | 0.00225653 |
| COPE | 0.60916665 | 1.63E-05 | 0.00225653 |
| FAM20C | 0.61686496 | 1.67E-05 | 0.00228304 |
| RPRD1A | -0.7357188 | 1.68E-05 | 0.00228304 |
| RPS21 | 0.70913609 | 1.71E-05 | 0.00229652 |
| ZNF267 | -0.8980097 | 1.80E-05 | 0.00239465 |
| VPS26A | -0.5931427 | 1.98E-05 | 0.00259164 |
| RNF11 | -0.6917707 | 1.99E-05 | 0.00259164 |
| PDLIM4 | 0.7070264 | 2.04E-05 | 0.00262302 |
| TSPAN4 | 0.66892416 | 2.10E-05 | 0.00262302 |
| AP001324.1 | 1.98118026 | 2.12E-05 | 0.00262302 |
| ELFN1 | 1.35946787 | 2.12E-05 | 0.00262302 |
| AP2S1 | 0.59161893 | 2.14E-05 | 0.00262302 |
| GET4 | 2.24720789 | 2.16E-05 | 0.00262302 |
| RPS29 | 0.65227421 | 2.16E-05 | 0.00262302 |
| JMJD8 | 0.71213595 | 2.21E-05 | 0.00262738 |
| MFAP3 | -0.8052119 | 2.21E-05 | 0.00262738 |
| MAZ | 0.57130989 | 2.28E-05 | 0.00268366 |
| PIGK | -0.78364 | 2.43E-05 | 0.00281088 |
| GPR153 | 0.69337574 | 2.43E-05 | 0.00281088 |
| TSPO | 0.59139648 | 2.46E-05 | 0.00281088 |
| CCDC85B | 0.85609815 | 2.48E-05 | 0.00281088 |
| DTWD1 | -1.594724 | 2.52E-05 | 0.00283322 |
| H1FX | 0.68001464 | 2.54E-05 | 0.00283353 |
| SCAND1 | 0.80478737 | 2.60E-05 | 0.00286828 |
| ELMOD2 | -0.9804137 | 2.64E-05 | 0.0028913 |
| CBX4 | 0.70252195 | 3.10E-05 | 0.00336737 |
| RPL28 | 0.74625596 | 3.20E-05 | 0.0034429 |
| C11orf68 | 0.66891604 | 3.36E-05 | 0.0035241 |
| NME4 | 0.70947298 | 3.36E-05 | 0.0035241 |
| FBXL15 | 0.79534942 | 3.38E-05 | 0.0035241 |
| TMEM170B | -0.9643058 | 3.42E-05 | 0.0035241 |
| GOLGA8A | -0.7965054 | 3.42E-05 | 0.0035241 |
| UMAD1 | -0.9350896 | 3.44E-05 | 0.0035241 |
| STUB1 | 0.60375287 | 3.50E-05 | 0.00354837 |
| THOC1 | -0.7453758 | 3.85E-05 | 0.0038739 |
| MRPS12 | 0.78392733 | 3.90E-05 | 0.00389158 |
| ATP5O | -0.5808201 | 3.97E-05 | 0.00390367 |
| DGKH | -0.806815 | 3.97E-05 | 0.00390367 |
| WFS1 | 0.61710271 | 4.06E-05 | 0.00396158 |
| ARHGAP1 | 0.57068697 | 4.24E-05 | 0.00410464 |
| GOLGB1 | -0.5661876 | 4.29E-05 | 0.00411913 |
| ROGDI | 0.59999636 | 4.36E-05 | 0.00415963 |
| RBM48 | -0.7922419 | 4.60E-05 | 0.00435254 |
| GOPC | -0.8741861 | 4.65E-05 | 0.00436263 |
| ORMDL1 | -0.5811369 | 4.70E-05 | 0.00437841 |
| DRAP1 | 0.54762115 | 4.77E-05 | 0.00440103 |
| TMBIM4 | -1.095671 | 4.81E-05 | 0.00440103 |
| AC026366.1 | 0.71529144 | 4.89E-05 | 0.00440103 |
| AIMP1 | -0.6075689 | 4.97E-05 | 0.00440103 |
| ZWILCH | -0.881332 | 5.01E-05 | 0.00440103 |
| TAF1D | -0.6870082 | 5.02E-05 | 0.00440103 |
| TIMM13 | 0.73373164 | 5.03E-05 | 0.00440103 |
| VAMP5 | 0.99551082 | 5.06E-05 | 0.00440103 |
| NDUFA5 | -0.8565495 | 5.13E-05 | 0.00440103 |
| RPL9P9 | -0.672521 | 5.13E-05 | 0.00440103 |
| CHORDC1 | -0.6649791 | 5.14E-05 | 0.00440103 |
| ENGASE | 0.64867577 | 5.15E-05 | 0.00440103 |
| C20orf27 | 0.65929594 | 5.28E-05 | 0.00446314 |
| MSRB1 | 0.58474712 | 5.31E-05 | 0.00446314 |
| ZBTB41 | -1.0864562 | 5.33E-05 | 0.00446314 |
| CCL1 | 3.09069648 | 5.60E-05 | 0.00463831 |
| LCORL | -0.925327 | 5.64E-05 | 0.00463831 |
| MT2A | 0.93681325 | 5.65E-05 | 0.00463831 |
| CEBPA | 0.58740509 | 5.70E-05 | 0.00464788 |
| TMUB1 | 0.77810775 | 5.74E-05 | 0.00465186 |
| PGLS | 0.55837915 | 5.91E-05 | 0.00476114 |
| DGAT1 | 0.6140202 | 6.10E-05 | 0.00486968 |
| DOK1 | 0.56571235 | 6.12E-05 | 0.00486968 |
| BROX | -0.699197 | 6.16E-05 | 0.00486968 |
| RAMP1 | 0.57291064 | 6.31E-05 | 0.00495367 |
| PEMT | 0.72546282 | 6.38E-05 | 0.00497652 |
| MIIP | 0.5878605 | 6.52E-05 | 0.00505264 |
| AP1AR | -0.6847719 | 6.63E-05 | 0.00510681 |
| HSPB1 | 0.64327 | 6.79E-05 | 0.00520007 |
| BATF3 | 0.5847092 | 6.85E-05 | 0.00521279 |
| POLD4 | 1.16770245 | 7.07E-05 | 0.00535209 |
| ANP32E | -0.7649245 | 7.28E-05 | 0.0054768 |
| TSNAX | -0.7389314 | 7.40E-05 | 0.00552915 |
| POLR2L | 0.69293599 | 7.63E-05 | 0.00565041 |
| OSTM1 | -0.6417233 | 7.67E-05 | 0.00565041 |
| CUTA | 0.57279871 | 7.69E-05 | 0.00565041 |
| RELL2 | 0.86223927 | 7.87E-05 | 0.00571857 |
| PTAR1 | -0.7923968 | 7.88E-05 | 0.00571857 |
| USF3 | -0.6395295 | 8.17E-05 | 0.00589425 |
| NANP | -1.003774 | 8.32E-05 | 0.00597099 |
| IER5L | 0.76798271 | 8.39E-05 | 0.00598281 |
| CENPB | 0.63786396 | 8.70E-05 | 0.00616816 |
| SIPA1 | 0.66479988 | 8.80E-05 | 0.00621001 |
| TMPO | -0.5748342 | 8.98E-05 | 0.00623666 |
| TMEM158 | 0.88366302 | 9.02E-05 | 0.00623666 |
| C5orf24 | -0.7984096 | 9.04E-05 | 0.00623666 |
| CYC1 | 0.52718551 | 9.17E-05 | 0.00623666 |
| AP001024.1 | 2.48381018 | 9.19E-05 | 0.00623666 |
| TDP2 | -0.5183945 | 9.21E-05 | 0.00623666 |
| NFAM1 | 0.54059706 | 9.22E-05 | 0.00623666 |
| LY96 | -0.7050256 | 9.24E-05 | 0.00623666 |
| TRMT112 | 0.66057698 | 9.48E-05 | 0.0063646 |
| EIF1AX | -0.6976301 | 9.63E-05 | 0.0064074 |
| ENHO | 0.7612905 | 9.65E-05 | 0.0064074 |
| UNC93B1 | 0.53622916 | 9.76E-05 | 0.00641235 |
| TXNDC9 | -0.6500968 | 9.76E-05 | 0.00641235 |
| CDK10 | 0.76668095 | 9.83E-05 | 0.00642706 |
| NFYB | -0.6792479 | 9.99E-05 | 0.00649558 |
| MROH1 | 0.51957856 | 0.00010119 | 0.00649558 |
| GTPBP8 | -0.935929 | 0.00010125 | 0.00649558 |
| IDE | -0.621006 | 0.00010146 | 0.00649558 |
| ZNF780A | -0.8934539 | 0.00010415 | 0.00663399 |
| ATP5EP2 | 2.4128811 | 0.00010594 | 0.00671379 |
| NACC1 | 0.51250383 | 0.00010656 | 0.00671882 |
| TRMT61A | 0.79546795 | 0.00010718 | 0.0067239 |
| SLC25A29 | 0.50268983 | 0.00011019 | 0.00687794 |
| SCML1 | -0.9080822 | 0.00011173 | 0.00691775 |
| C19orf60 | 0.66091023 | 0.00011275 | 0.00691775 |
| MAN1A2 | -0.6164811 | 0.00011303 | 0.00691775 |
| UNC13D | 0.51926403 | 0.00011324 | 0.00691775 |
| RFX1 | 0.87031244 | 0.0001136 | 0.00691775 |
| TMA7 | 0.83298571 | 0.00011457 | 0.00694289 |
| FAAP100 | 0.64892729 | 0.00011515 | 0.00694475 |
| TOPORS | -0.6014816 | 0.00011667 | 0.00700266 |
| GIT1 | 0.59863794 | 0.00012098 | 0.00722653 |
| SCAP | 0.60139096 | 0.00012277 | 0.00729865 |
| ZFYVE16 | -0.6206619 | 0.00013226 | 0.00781358 |
| SLC25A24 | -0.580111 | 0.00013269 | 0.00781358 |
| SMC5 | -0.749584 | 0.00013483 | 0.0078741 |
| MIGA1 | -1.0586483 | 0.00013498 | 0.0078741 |
| SLC19A1 | 0.81073093 | 0.00013578 | 0.00788389 |
| GXYLT1 | -0.8688529 | 0.00013841 | 0.00797174 |
| RPS25 | 0.57283773 | 0.00013857 | 0.00797174 |
| SPINT1 | 0.52978231 | 0.00014033 | 0.00803623 |
| ZNF254 | -0.9597814 | 0.00014357 | 0.00816842 |
| MIB1 | -0.6691716 | 0.00014395 | 0.00816842 |
| MYPOP | 0.95817879 | 0.00014467 | 0.00817218 |
| MCRIP1 | 0.60448405 | 0.00015008 | 0.0084398 |
| SNAI3 | 0.77335052 | 0.0001515 | 0.00848107 |
| RPE | -0.6260397 | 0.00015318 | 0.00853699 |
| LTBR | 0.49025522 | 0.00015817 | 0.00870531 |
| ZNF800 | -0.5657515 | 0.00015944 | 0.00870531 |
| RPLP1 | 0.64917414 | 0.00016006 | 0.00870531 |
| PSMA1 | -0.7561339 | 0.00016052 | 0.00870531 |
| RGS19 | 0.63264226 | 0.00016069 | 0.00870531 |
| C7orf43 | 0.64425811 | 0.00016083 | 0.00870531 |
| IKBKG | 1.07047505 | 0.00016108 | 0.00870531 |
| QPCTL | 1.04781988 | 0.00016211 | 0.00872328 |
| ZNF518A | -0.6971035 | 0.00016427 | 0.00876057 |
| ZBTB47 | 0.79009689 | 0.00016434 | 0.00876057 |
| ANKRD49 | -0.6844014 | 0.00016491 | 0.00876057 |
| CYBA | 0.60035201 | 0.00016627 | 0.00879517 |
| ZBTB7A | 0.54392619 | 0.00016748 | 0.00882205 |
| ZNF180 | -0.8670346 | 0.0001683 | 0.0088278 |
| C11orf24 | 0.6464567 | 0.00017079 | 0.00892095 |
| DCXR | 0.61197494 | 0.00017275 | 0.00898364 |
| BCR | 0.66876072 | 0.00017451 | 0.00898364 |
| CEP290 | -1.5923871 | 0.00017479 | 0.00898364 |
| MALAT1 | -0.5657928 | 0.00017487 | 0.00898364 |
| DNPH1 | 0.52059129 | 0.00017719 | 0.00906562 |
| DCAF15 | 0.5951833 | 0.00017851 | 0.00909586 |
| SLC25A40 | -0.6977838 | 0.00018154 | 0.0092127 |
| ARL2 | 0.72221044 | 0.00018495 | 0.00932338 |
| CLN5 | -0.8120086 | 0.00018574 | 0.00932338 |
| TRIM8 | 0.56571475 | 0.00018596 | 0.00932338 |
| CHST13 | 0.93354109 | 0.00018932 | 0.00945385 |
| TMEM250 | 0.62635216 | 0.00019031 | 0.00946542 |
| RCN1 | -0.701682 | 0.00019789 | 0.0097724 |
| DENND4A | -0.6568583 | 0.00019839 | 0.0097724 |
| RTN4R | 0.88704456 | 0.00019883 | 0.0097724 |
| IRAK3 | -0.6407139 | 0.00020712 | 0.01013994 |
| OSCAR | 0.50503067 | 0.0002082 | 0.01014535 |
| ZNF24 | -0.5885681 | 0.000209 | 0.01014535 |
| AKAP11 | -0.5669587 | 0.00021063 | 0.01014535 |
| NENF | 0.57882985 | 0.00021078 | 0.01014535 |
| MIS18BP1 | -0.8585053 | 0.00021129 | 0.01014535 |
| XIAP | -0.5820189 | 0.00021681 | 0.0102441 |
| TRIM11 | 0.54669243 | 0.00021825 | 0.0102441 |
| IGSF8 | 0.595546 | 0.00021856 | 0.0102441 |
| LRP3 | 0.77306984 | 0.00021915 | 0.0102441 |
| ACAP2 | -0.5568174 | 0.00021919 | 0.0102441 |
| PCBD1 | 0.5021926 | 0.00021975 | 0.0102441 |
| NAB2 | 0.54386673 | 0.00021988 | 0.0102441 |
| PAX8 | 0.8445049 | 0.00022082 | 0.0102441 |
| IMPA1 | -0.8788517 | 0.00022103 | 0.0102441 |
| ACD | 0.79855747 | 0.00022187 | 0.0102441 |
| COMT | 0.51581008 | 0.00022271 | 0.0102441 |
| SNX13 | -0.5527744 | 0.0002232 | 0.0102441 |
| BAX | 0.47863261 | 0.00022422 | 0.01025355 |
| HCST | 0.68290729 | 0.00022697 | 0.01034114 |
| U2SURP | -0.5324126 | 0.00023205 | 0.01051925 |
| USE1 | 0.85674644 | 0.00023333 | 0.01051925 |
| TFAM | -0.7314776 | 0.00023371 | 0.01051925 |
| COMMD4 | 0.67513546 | 0.00023438 | 0.01051925 |
| HSF1 | 0.50831688 | 0.0002352 | 0.01051925 |
| LRRC47 | 0.57578363 | 0.00023595 | 0.01051925 |
| PPP1R16A | 0.65860284 | 0.00023678 | 0.01051925 |
| RPL41 | 1.08139564 | 0.00024706 | 0.01093717 |
| CCDC117 | -0.6086946 | 0.00025093 | 0.01106909 |
| CEBPB | 0.85798805 | 0.00025197 | 0.0110762 |
| KIAA1586 | -1.04827 | 0.00025312 | 0.01108745 |
| LARP1B | -0.641238 | 0.00025409 | 0.01109104 |
| ARRDC1 | 0.69063422 | 0.00025556 | 0.01111635 |
| CIC | 0.4828256 | 0.00025823 | 0.01119363 |
| LRRC14 | 0.6308539 | 0.00026102 | 0.01127535 |
| PQLC1 | 0.48937966 | 0.00026342 | 0.01133995 |
| RSPRY1 | -0.5322461 | 0.00026681 | 0.01144638 |
| DCUN1D1 | -0.6734091 | 0.00026775 | 0.01144726 |
| ZBTB33 | -0.6658581 | 0.0002696 | 0.0114869 |
| TPRA1 | 0.55986761 | 0.00027416 | 0.01160851 |
| CD68 | 1.09445631 | 0.00027455 | 0.01160851 |
| SNX17 | 0.48017527 | 0.00027524 | 0.01160851 |
| GNPNAT1 | -0.8917368 | 0.00027634 | 0.01161564 |
| PSMB10 | 0.56737498 | 0.00027809 | 0.01164974 |
| GEN1 | -0.9447465 | 0.00028006 | 0.01169311 |
| CCDC90B | -0.589568 | 0.00028132 | 0.01170678 |
| STK32C | 0.58014632 | 0.00028368 | 0.0117121 |
| LZTS2 | 0.73350799 | 0.000286 | 0.0117121 |
| GPS1 | 0.581822 | 0.00028618 | 0.0117121 |
| CTU1 | 1.2953087 | 0.00028692 | 0.0117121 |
| UBA7 | 0.49461819 | 0.00028697 | 0.0117121 |
| MFSD8 | -0.7746032 | 0.00028708 | 0.0117121 |
| Mar/02 | 0.50695156 | 0.0002898 | 0.01178469 |
| MAPK6 | -0.581096 | 0.00029167 | 0.01181089 |
| TRABD | 0.50866945 | 0.00029234 | 0.01181089 |
| CALHM2 | 0.53144183 | 0.00029653 | 0.0118902 |
| LAMTOR4 | 0.50352731 | 0.00029657 | 0.0118902 |
| AC073621.1 | 1.26365804 | 0.00029763 | 0.0118902 |
| SH2B1 | 0.58397811 | 0.00029869 | 0.0118902 |
| CYP51A1 | 0.96360283 | 0.00029906 | 0.0118902 |
| PSIP1 | -0.6611546 | 0.0003025 | 0.0119887 |
| PPIP5K2 | -0.6521333 | 0.00030677 | 0.01208068 |
| C1orf122 | 0.5675649 | 0.00030753 | 0.01208068 |
| VPS18 | 0.50339775 | 0.00030785 | 0.01208068 |
| FAM114A2 | -0.7611488 | 0.00031193 | 0.01208068 |
| DR1 | -0.5545788 | 0.00031303 | 0.01208068 |
| TMEM150B | 0.87215325 | 0.0003136 | 0.01208068 |
| CD151 | 0.51792557 | 0.00031387 | 0.01208068 |
| MCRIP2 | 0.70903093 | 0.00031412 | 0.01208068 |
| ABCA7 | 0.59466584 | 0.00031484 | 0.01208068 |
| RHPN1 | 0.70533666 | 0.00031534 | 0.01208068 |
| MOB4 | -0.8764064 | 0.00031602 | 0.01208068 |
| AC019069.1 | 1.53654109 | 0.0003165 | 0.01208068 |
| PYCARD | 0.58089839 | 0.00031805 | 0.01208068 |
| NDC1 | -0.6616402 | 0.00031883 | 0.01208068 |
| PHLDA1 | -0.4773509 | 0.00031934 | 0.01208068 |
| YEATS4 | -0.8534298 | 0.00032215 | 0.01212741 |
| TWF2 | 0.53579116 | 0.00032252 | 0.01212741 |
| MAP1S | 0.4882498 | 0.00032735 | 0.01227226 |
| ASB6 | 0.5897988 | 0.00032928 | 0.01230768 |
| PPP1R14B | 0.56878367 | 0.00033055 | 0.01231812 |
| JUND | 0.55470552 | 0.00033327 | 0.01238263 |
| ZNF219 | 0.66446383 | 0.00033712 | 0.01248827 |
| REPIN1 | 0.48884942 | 0.0003413 | 0.01260573 |
| VPS9D1 | 0.51341558 | 0.0003428 | 0.01262376 |
| VBP1 | -0.6126949 | 0.00034456 | 0.01265154 |
| KAT2B | -0.5295114 | 0.00034703 | 0.01270489 |
| FPGT | -0.9005716 | 0.00035006 | 0.01277832 |
| JUP | 0.48590749 | 0.00035294 | 0.01282944 |
| KIFC2 | 0.5414492 | 0.00035352 | 0.01282944 |
| ZNF710 | 0.49185475 | 0.00035523 | 0.01285416 |
| GPAM | -1.0668816 | 0.00036031 | 0.01293284 |
| LMBRD2 | -0.7296561 | 0.00036123 | 0.01293284 |
| MAN2A1 | -0.5581384 | 0.00036148 | 0.01293284 |
| ROMO1 | 0.61153896 | 0.00036155 | 0.01293284 |
| ZNHIT1 | 0.52955785 | 0.00036406 | 0.01295447 |
| TRAPPC6B | -0.670543 | 0.0003643 | 0.01295447 |
| MKLN1 | -0.5005858 | 0.00036527 | 0.01295447 |
| FAM26F | 0.84056263 | 0.0003681 | 0.01301808 |
| OXLD1 | 0.74761263 | 0.00037023 | 0.01303234 |
| ZFAND1 | -0.8097412 | 0.00037059 | 0.01303234 |
| TMEM258 | 0.65136554 | 0.00037253 | 0.01306358 |
| PIEZO1 | 0.47807776 | 0.00038454 | 0.01338066 |
| HDHD2 | -1.3762014 | 0.00038467 | 0.01338066 |
| LSM5 | -0.6316249 | 0.00038522 | 0.01338066 |
| OST4 | 0.55579997 | 0.00038598 | 0.01338066 |
| MRC2 | 0.44153535 | 0.00038693 | 0.01338066 |
| ALG6 | -1.3591272 | 0.00038854 | 0.01339917 |
| N4BP2 | -0.809605 | 0.00039006 | 0.01341457 |
| BCKDK | 0.50548147 | 0.00039121 | 0.01341716 |
| BAK1 | 0.53037796 | 0.00039282 | 0.01343552 |
| MAD1L1 | 0.5995039 | 0.00039489 | 0.01346668 |
| BAHCC1 | 0.78836036 | 0.00039589 | 0.01346668 |
| SELENOI | -0.6170453 | 0.00040274 | 0.01360379 |
| USP37 | -0.6584112 | 0.00040288 | 0.01360379 |
| SEC62 | -0.5765156 | 0.00040319 | 0.01360379 |
| TRMT10C | -0.6521943 | 0.00040574 | 0.01365291 |
| ADRM1 | 0.46500939 | 0.00040692 | 0.01365604 |
| PANK3 | -0.7718084 | 0.00040845 | 0.01367047 |
| GOLT1B | -1.0984623 | 0.00040984 | 0.01368024 |
| TMEM87B | -0.6116567 | 0.00041303 | 0.01372995 |
| RYK | -0.6522857 | 0.00041514 | 0.01372995 |
| TSC22D4 | 0.47211867 | 0.00041529 | 0.01372995 |
| ARNTL2 | -0.5479519 | 0.00041573 | 0.01372995 |
| DIABLO | 1.61909944 | 0.00041853 | 0.01377875 |
| SLC30A5 | -0.6537097 | 0.00041941 | 0.01377875 |
| ABCD3 | -0.6025677 | 0.00042365 | 0.01388161 |
| ZMPSTE24 | -0.7887262 | 0.00042612 | 0.01390535 |
| RCN2 | -0.7381691 | 0.00042661 | 0.01390535 |
| DPM1 | -0.5198288 | 0.00043218 | 0.01405051 |
| MEF2D | 0.53598611 | 0.00043342 | 0.01405413 |
| RPS13P2 | 1.43072752 | 0.00043545 | 0.01408319 |
| FAIM2 | 0.62403718 | 0.00044183 | 0.01425266 |
| PPDPF | 0.54882093 | 0.00044977 | 0.01444625 |
| GEMIN8 | 1.01124049 | 0.00045014 | 0.01444625 |
| ESRRA | 0.57975451 | 0.00045238 | 0.0144807 |
| CLIP2 | 0.46617642 | 0.00045625 | 0.01456734 |
| ATP6V1C1 | -0.5359178 | 0.00045923 | 0.01458897 |
| PSMA2 | -0.7506528 | 0.00045982 | 0.01458897 |
| UQCRQ | 0.52818257 | 0.00046217 | 0.01458897 |
| CPSF2 | -0.4614634 | 0.00046272 | 0.01458897 |
| GALNT18 | 0.44658397 | 0.00046277 | 0.01458897 |
| SPSB1 | 0.53407672 | 0.00046684 | 0.01468032 |
| CDC73 | -0.5622458 | 0.00047367 | 0.01483973 |
| PCNX4 | -0.5397098 | 0.00047511 | 0.01483973 |
| PHLDA3 | 0.74206582 | 0.00047706 | 0.01483973 |
| ECI1 | 0.61926938 | 0.00047729 | 0.01483973 |
| REEP4 | 0.59899009 | 0.00047885 | 0.01483973 |
| LINC00116 | 0.97793287 | 0.00048002 | 0.01483973 |
| RC3H1 | -0.5585829 | 0.00048023 | 0.01483973 |
| PXN | 0.48220834 | 0.00048558 | 0.01496799 |
| IFI27L2 | 0.84133584 | 0.00048899 | 0.01503577 |
| STX10 | 0.50420969 | 0.00049325 | 0.01512943 |
| ZDHHC2 | -0.6283672 | 0.00049625 | 0.01514118 |
| CLTB | 0.52442296 | 0.00049656 | 0.01514118 |
| EIF5A | 0.49831912 | 0.00049727 | 0.01514118 |
| FAM102B | -0.5218985 | 0.00050043 | 0.01520034 |
| ZNF761 | -0.7133985 | 0.00050253 | 0.01522609 |
| GCC2 | -0.6763324 | 0.00050383 | 0.01522609 |
| POC1B | -0.7149707 | 0.00050493 | 0.01522609 |
| AC067931.2 | 2.37514261 | 0.00050759 | 0.01523067 |
| THAP5 | -0.8512911 | 0.00050832 | 0.01523067 |
| MED25 | 0.51205588 | 0.00051017 | 0.01523067 |
| PUS7L | -0.880128 | 0.0005104 | 0.01523067 |
| ACTR1B | 0.49912933 | 0.00051149 | 0.01523067 |
| MT-ATP8 | 1.22683209 | 0.00051241 | 0.01523067 |
| TTC7A | 0.53969346 | 0.0005163 | 0.01531006 |
| LBR | -0.5868692 | 0.0005217 | 0.01543341 |
| TROVE2 | -0.6162347 | 0.00052428 | 0.0154584 |
| NDUFB10 | 0.56726853 | 0.00052502 | 0.0154584 |
| ALG10B | -1.6583231 | 0.0005292 | 0.01554473 |
| NAGLU | 0.58564263 | 0.00053198 | 0.01556801 |
| RPS19P3 | 2.66095607 | 0.00053355 | 0.01556801 |
| SLC26A11 | 0.48676556 | 0.00053373 | 0.01556801 |
| SLC11A1 | 0.79332411 | 0.00053727 | 0.01563479 |
| Z83843.1 | -1.2850172 | 0.00053857 | 0.01563617 |
| TMEM11 | 0.57666048 | 0.00054648 | 0.01582893 |
| SBDS | -0.4896716 | 0.00055156 | 0.01593901 |
| ZBTB7B | 0.4622322 | 0.00055797 | 0.01605208 |
| CYHR1 | 0.50157235 | 0.00055804 | 0.01605208 |
| ZNF345 | -1.8322069 | 0.00056413 | 0.01619002 |
| TESK1 | 0.47189185 | 0.00056649 | 0.01621488 |
| RRM2B | -0.642752 | 0.0005676 | 0.01621488 |
| GOLGA4 | -0.6074073 | 0.00057133 | 0.01622557 |
| SCYL1 | 0.47174514 | 0.000573 | 0.01622557 |
| DNAJC10 | -0.4635748 | 0.00057328 | 0.01622557 |
| DDAH2 | 0.52799299 | 0.00057431 | 0.01622557 |
| RNPC3 | -0.7403675 | 0.00057447 | 0.01622557 |
| TRAPPC1 | 0.51498649 | 0.00057801 | 0.01628852 |
| DDX3Y | -0.5168605 | 0.0005824 | 0.01637536 |
| UBE2S | 1.3130872 | 0.0005858 | 0.01637787 |
| AK6 | -0.7267914 | 0.00058643 | 0.01637787 |
| ZNF37A | -0.5555379 | 0.00058754 | 0.01637787 |
| NR1H2 | 0.49171207 | 0.00058857 | 0.01637787 |
| CDC34 | 0.52105133 | 0.00058905 | 0.01637787 |
| TMEM8A | 0.51950015 | 0.00059423 | 0.01645998 |
| SLIRP | 0.59120083 | 0.00059464 | 0.01645998 |
| FAM172A | -0.805924 | 0.00059676 | 0.01648211 |
| RABGGTB | -0.4727958 | 0.00060113 | 0.01656624 |
| NUMBL | 0.75774926 | 0.00060504 | 0.0166236 |
| RRN3 | -0.5417817 | 0.00060587 | 0.0166236 |
| HIGD2A | 0.43963697 | 0.00060824 | 0.01665192 |
| NUDT1 | 0.71054779 | 0.00061096 | 0.01668979 |
| TOX2 | 1.25408161 | 0.00061469 | 0.01675498 |
| IFNL1 | 3.20053764 | 0.00061607 | 0.01675596 |
| MIER1 | -0.5594833 | 0.00061935 | 0.01680513 |
| RAP2C | -0.7925411 | 0.00062057 | 0.01680513 |
| BRCA2 | -0.8476854 | 0.00062199 | 0.01680731 |
| IFI27 | 2.32941627 | 0.00062431 | 0.01683349 |
| GALNT1 | -0.4950478 | 0.00062804 | 0.01686664 |
| EXOSC4 | 0.73398125 | 0.00062824 | 0.01686664 |
| AC006064.4 | 2.5152766 | 0.00063091 | 0.01690177 |
| LRRC8B | -0.5327633 | 0.00063234 | 0.01690389 |
| TEX264 | 0.52747561 | 0.000634 | 0.01691198 |
| C3orf58 | -1.0059959 | 0.00063782 | 0.01697784 |
| YIF1B | 0.54473721 | 0.00063925 | 0.01697955 |
| CMTM7 | 0.52137966 | 0.0006429 | 0.01704025 |
| ZNF430 | -0.5923106 | 0.00064689 | 0.01708471 |
| GPX1 | 0.55079632 | 0.00064731 | 0.01708471 |
| SLC12A9 | 0.48882714 | 0.00065257 | 0.017138 |
| CASD1 | -1.2395887 | 0.00065324 | 0.017138 |
| VPS54 | -0.526655 | 0.00065345 | 0.017138 |
| RIN3 | 0.49152356 | 0.00065534 | 0.01715142 |
| IDUA | 0.83797059 | 0.00066144 | 0.01727019 |
| FAM131A | 0.46811278 | 0.00066324 | 0.01727019 |
| Mar/07 | -0.495126 | 0.00066403 | 0.01727019 |
| RHOT2 | 0.49472042 | 0.00066656 | 0.01730006 |
| NDUFS7 | 0.53230259 | 0.00066863 | 0.01731783 |
| G3BP1 | -0.5434946 | 0.00067024 | 0.01732362 |
| ZDHHC20 | -0.5903901 | 0.00067357 | 0.01737355 |
| SH3GL1 | 0.46292554 | 0.00067669 | 0.01741813 |
| KLF16 | 0.60097014 | 0.0006813 | 0.01750081 |
| SMIM29 | 0.73678514 | 0.00068777 | 0.01761249 |
| PPP3R1 | -0.6019087 | 0.00068847 | 0.01761249 |
| MAP7D1 | 0.49508754 | 0.00069092 | 0.01762785 |
| AGTRAP | 0.67275615 | 0.00069411 | 0.01762785 |
| SLC25A1 | 0.54441681 | 0.00069485 | 0.01762785 |
| RPL18 | 0.48759322 | 0.00069571 | 0.01762785 |
| FP565260.7 | -2.0743955 | 0.00069613 | 0.01762785 |
| DTWD2 | -1.0371185 | 0.00069791 | 0.01763706 |
| GON7 | -0.763155 | 0.0007046 | 0.01775156 |
| OXR1 | -0.6293782 | 0.00070671 | 0.01775156 |
| DCK | -0.6400893 | 0.000709 | 0.01775156 |
| ERGIC2 | -0.6703474 | 0.00071 | 0.01775156 |
| NCK1 | -0.4599602 | 0.0007106 | 0.01775156 |
| FBXO28 | -0.7410404 | 0.00071289 | 0.01775156 |
| PHTF2 | -0.5866051 | 0.00071363 | 0.01775156 |
| PIGT | 0.4738468 | 0.00071483 | 0.01775156 |
| NIPSNAP2 | -0.4613574 | 0.00071648 | 0.01775156 |
| EID3 | -2.7351327 | 0.00071666 | 0.01775156 |
| ZRANB2 | -0.7406217 | 0.00072012 | 0.01780187 |
| ZNF316 | 0.50484915 | 0.00072558 | 0.0179015 |
| ABI3 | 0.9304468 | 0.00072828 | 0.01793256 |
| PTGER2 | -0.585014 | 0.0007328 | 0.0180085 |
| IGHD | 1.82669517 | 0.00073533 | 0.01803513 |
| ACOT8 | 0.6354489 | 0.00073798 | 0.01806458 |
| NOTCH3 | 0.82113485 | 0.00074112 | 0.01807222 |
| GPAA1 | 0.4939884 | 0.00074553 | 0.01807222 |
| FBXL6 | 0.79721508 | 0.00074607 | 0.01807222 |
| DENND6A | -0.696315 | 0.00074651 | 0.01807222 |
| SDHAF1 | 0.75461271 | 0.00074724 | 0.01807222 |
| CHMP2B | -0.7233029 | 0.00074805 | 0.01807222 |
| CDK2AP2 | 0.54096343 | 0.00074885 | 0.01807222 |
| ZBTB10 | -0.8863321 | 0.00075167 | 0.01807222 |
| AGFG2 | 0.61680524 | 0.00075231 | 0.01807222 |
| SNAI1 | 2.40650925 | 0.00075277 | 0.01807222 |
| PRSS23 | 1.3963324 | 0.00075525 | 0.01807734 |
| TMEM259 | 0.46747543 | 0.00075588 | 0.01807734 |
| SLC26A6 | 0.49566137 | 0.00075946 | 0.0181126 |
| AQP3 | 0.43684333 | 0.00076025 | 0.0181126 |
| HSD3B7 | 0.45830178 | 0.00076697 | 0.01821145 |
| RBM41 | -0.8068246 | 0.00076732 | 0.01821145 |
| UBALD1 | 0.67944596 | 0.0007707 | 0.0182512 |
| FBXW5 | 0.43447253 | 0.00077229 | 0.0182512 |
| C19orf53 | 0.54055999 | 0.00077482 | 0.0182512 |
| HCFC1R1 | 0.65481592 | 0.00077484 | 0.0182512 |
| SLC30A7 | -0.812084 | 0.00077955 | 0.01832744 |
| ZBTB26 | -0.8791045 | 0.00078447 | 0.01840819 |
| ALKBH7 | 0.64791765 | 0.00078806 | 0.01840819 |
| LYL1 | 0.64222418 | 0.00078953 | 0.01840819 |
| CD200R1 | -0.5788903 | 0.00079095 | 0.01840819 |
| FHL3 | 0.57140681 | 0.0007916 | 0.01840819 |
| RABAC1 | 0.62269217 | 0.00079183 | 0.01840819 |
| SUCLA2 | -0.6057257 | 0.00079725 | 0.01848823 |
| AL671883.3 | 2.22312846 | 0.00079823 | 0.01848823 |
| FGFRL1 | 0.72038412 | 0.00080397 | 0.01856891 |
| BDP1 | -0.4828747 | 0.00080469 | 0.01856891 |
| NKIRAS1 | -0.7489471 | 0.00080807 | 0.01861238 |
| SYNGR1 | 1.08339963 | 0.00081521 | 0.01871082 |
| NTHL1 | 0.96829572 | 0.00081535 | 0.01871082 |
| TPD52 | -0.9693526 | 0.00081684 | 0.01871082 |
| PPP1CC | -0.5357251 | 0.00081859 | 0.01871661 |
| MBD3 | 0.45294459 | 0.00082421 | 0.01881062 |
| TNFRSF12A | 0.743445 | 0.00082587 | 0.01881417 |
| ZDHHC21 | -1.0239685 | 0.00082798 | 0.01882789 |
| KRCC1 | -0.580788 | 0.00083344 | 0.01884192 |
| IFFO2 | 0.51049443 | 0.00083356 | 0.01884192 |
| C3orf18 | 0.73385898 | 0.00083359 | 0.01884192 |
| RIT1 | -0.5680476 | 0.00083464 | 0.01884192 |
| MORC3 | -0.5412978 | 0.00083679 | 0.01885643 |
| NAT14 | 2.06638526 | 0.00084049 | 0.01889391 |
| SHKBP1 | 0.43850385 | 0.00084395 | 0.01889391 |
| CNTLN | -0.6614743 | 0.00084416 | 0.01889391 |
| SNX14 | -0.5568958 | 0.00084494 | 0.01889391 |
| ZNF354A | -0.9694291 | 0.00084602 | 0.01889391 |
| PAXBP1 | -0.5675087 | 0.00084918 | 0.0189307 |
| SNAPC2 | 0.6095092 | 0.00085111 | 0.01893977 |
| CCDC12 | 0.55172388 | 0.00085612 | 0.01899561 |
| MGRN1 | 0.4468453 | 0.00085689 | 0.01899561 |
| ANKRD37 | 0.6034773 | 0.00085818 | 0.01899561 |
| PTPA | 0.43496018 | 0.00086351 | 0.01907972 |
| RPS6KA4 | 0.46815128 | 0.00086802 | 0.01913014 |
| ANO8 | 0.7511018 | 0.00086899 | 0.01913014 |
| FAM171B | -2.1389909 | 0.00087039 | 0.01913014 |
| REEP3 | -0.6456126 | 0.00087394 | 0.01914716 |
| OIP5-AS1 | -0.553199 | 0.00087423 | 0.01914716 |
| FEZ2 | -0.5134324 | 0.00087603 | 0.019153 |
| KMT5C | 1.10167716 | 0.00088248 | 0.01926033 |
| ZNF292 | -0.5679783 | 0.00089465 | 0.01945235 |
| DUSP7 | 0.69296235 | 0.00089484 | 0.01945235 |
| TSTA3 | 0.53572755 | 0.00089596 | 0.01945235 |
| ARRDC3 | -0.9538927 | 0.00090125 | 0.01953329 |
| UBLCP1 | -0.5489769 | 0.00090634 | 0.01960961 |
| CCPG1 | -0.8768915 | 0.00091254 | 0.01970964 |
| NOL11 | -0.5310356 | 0.00091445 | 0.01971671 |
| FUOM | 0.56722195 | 0.00091695 | 0.01973666 |
| RNF187 | 0.48700351 | 0.0009331 | 0.02004952 |
| ATAD1 | -0.5149871 | 0.00094035 | 0.02017062 |
| TMEM161B | -0.8803758 | 0.00095556 | 0.02046167 |
| ADAP1 | 0.53754894 | 0.00096004 | 0.0204695 |
| TIPRL | -0.5159168 | 0.00096012 | 0.0204695 |
| ERI2 | -0.7872572 | 0.00096084 | 0.0204695 |
| ATG4B | 0.4777945 | 0.00096816 | 0.0205409 |
| TSR3 | 0.62636676 | 0.00096905 | 0.0205409 |
| DGKQ | 0.69697737 | 0.00096913 | 0.0205409 |
| CD14 | 0.57378817 | 0.00097297 | 0.02058736 |
| DUSP23 | 0.54439881 | 0.00097525 | 0.02059604 |
| NR2F6 | 0.87974793 | 0.00097694 | 0.02059604 |
| ITPK1 | 0.45843945 | 0.00097904 | 0.02059604 |
| PSMD12 | -0.6103492 | 0.00098097 | 0.02059604 |
| TMEM203 | 0.62891308 | 0.00098212 | 0.02059604 |
| BMT2 | -0.6872419 | 0.00098328 | 0.02059604 |
| VPS28 | 0.52856632 | 0.00098644 | 0.02062758 |
| ERBIN | -0.5336191 | 0.00098975 | 0.02066234 |
| TGFBR1 | -0.5859084 | 0.0009916 | 0.02066639 |
| MAPK13 | 0.52893923 | 0.00099607 | 0.02070961 |
| NKG7 | 0.73186741 | 0.0009977 | 0.02070961 |
| INTS8 | -0.5329915 | 0.00099865 | 0.02070961 |
| BORCS7 | -0.9747316 | 0.00100385 | 0.02078276 |
| LACC1 | -1.0770592 | 0.00100595 | 0.02079182 |
| TIMM17B | 0.66959442 | 0.00100905 | 0.0208215 |
| STUM | 1.02260495 | 0.00101316 | 0.02087179 |
| GABPA | -0.7515783 | 0.00101768 | 0.02090605 |
| SSB | -0.4649139 | 0.00101817 | 0.02090605 |
| TSEN15 | -0.5718965 | 0.00102041 | 0.02091762 |
| NFXL1 | -0.5556471 | 0.00102342 | 0.02094491 |
| NAB1 | -0.5751354 | 0.00102513 | 0.02094557 |
| RASA2 | -0.6019536 | 0.00102999 | 0.02100831 |
| UBE3A | -0.506908 | 0.00103157 | 0.02100831 |
| STARD4 | -0.7996896 | 0.00103495 | 0.02104276 |
| NDUFAF8 | 0.92428412 | 0.00103756 | 0.0210617 |
| ZNF28 | -0.6248545 | 0.00104396 | 0.02115707 |
| NCBP2 | -0.4474035 | 0.00104863 | 0.02121725 |
| PPP4R3B | -0.5036857 | 0.00105047 | 0.02122019 |
| RNASE1 | 0.54685422 | 0.00105249 | 0.02122663 |
| INO80E | 0.62886832 | 0.00105802 | 0.02130373 |
| SHARPIN | 0.5860296 | 0.00106958 | 0.02150179 |
| HNRNPH2 | -0.4289163 | 0.00107472 | 0.02157041 |
| EIF4A1 | 0.79654362 | 0.00107962 | 0.02157851 |
| SLC6A8 | 0.77228379 | 0.00108133 | 0.02157851 |
| SLC26A2 | -0.5544114 | 0.00108214 | 0.02157851 |
| RPS2 | 0.49569104 | 0.00108438 | 0.02157851 |
| DAD1 | 0.44719415 | 0.00108449 | 0.02157851 |
| MAF1 | 0.43515071 | 0.00108549 | 0.02157851 |
| KYAT3 | -0.5560258 | 0.00108836 | 0.02160103 |
| EVI2A | -0.5689135 | 0.00110494 | 0.02187294 |
| TIGD5 | 0.74985872 | 0.00110556 | 0.02187294 |
| ZNF529 | -0.8148628 | 0.00111235 | 0.02197248 |
| FADD | 0.49052751 | 0.00112171 | 0.02212241 |
| CLN3 | 0.72183239 | 0.00114386 | 0.0224653 |
| ABCB9 | 0.91793142 | 0.00114477 | 0.0224653 |
| MED24 | 0.53388497 | 0.00114607 | 0.0224653 |
| PRKAR2B | -0.7670242 | 0.00114704 | 0.0224653 |
| HR | 0.75717736 | 0.0011481 | 0.0224653 |
| SNRPA | 0.52019231 | 0.001151 | 0.02246873 |
| NQO1 | -0.48574 | 0.00115187 | 0.02246873 |
| SLC29A1 | 0.68564636 | 0.00115764 | 0.02254597 |
| PLXND1 | 0.4781365 | 0.00116183 | 0.02259227 |
| MLLT1 | 0.59622855 | 0.00117347 | 0.02276196 |
| COX5B | 0.55235267 | 0.0011742 | 0.02276196 |
| CARHSP1 | 0.65616408 | 0.00118513 | 0.02293494 |
| RNF31 | 0.56299297 | 0.0011901 | 0.02293494 |
| KNTC1 | -0.7411685 | 0.00119047 | 0.02293494 |
| COMTD1 | 0.75295161 | 0.00119088 | 0.02293494 |
| THUMPD3-AS1 | -0.6902424 | 0.00119231 | 0.02293494 |
| NLRP1 | 0.47910102 | 0.00119859 | 0.02298913 |
| BCAP29 | -0.7697935 | 0.00119881 | 0.02298913 |
| TSN | -0.5168004 | 0.00120518 | 0.02304341 |
| MBNL3 | -0.6743233 | 0.00120673 | 0.02304341 |
| BRAT1 | 0.52825141 | 0.00120718 | 0.02304341 |
| ATPIF1 | 0.48774911 | 0.00120917 | 0.02304613 |
| ZMYND11 | -0.5264238 | 0.00121101 | 0.02304613 |
| RIPOR1 | 0.48875445 | 0.00121399 | 0.02306767 |
| CBLL1 | -0.5302625 | 0.00122481 | 0.02323782 |
| URI1 | -0.5013476 | 0.00122737 | 0.02325114 |
| SLC1A5 | 0.46477496 | 0.00123508 | 0.02336172 |
| COASY | 0.43691425 | 0.00123829 | 0.02338692 |
| PWWP2A | -0.5382612 | 0.00124939 | 0.02356095 |
| PLEKHA1 | -0.7650292 | 0.00125248 | 0.0235836 |
| CPNE3 | -0.5429401 | 0.00125718 | 0.02363249 |
| GCLM | -0.5752158 | 0.00125886 | 0.02363249 |
| TP53I13 | 0.49083825 | 0.00127079 | 0.02382063 |
| C5orf15 | -0.5259741 | 0.00127876 | 0.02393401 |
| ZNF354B | -1.1178865 | 0.00128632 | 0.02403957 |
| HINT3 | -0.5987051 | 0.00129248 | 0.02411847 |
| UBA3 | -0.5007501 | 0.00129715 | 0.0241695 |
| SLC15A3 | 0.54801573 | 0.00130659 | 0.02430928 |
| CKB | 0.51185589 | 0.00131106 | 0.02435615 |
| DIS3 | -0.4425383 | 0.00131575 | 0.02436804 |
| CD82 | 0.50282189 | 0.00131944 | 0.02436804 |
| EEF1B2 | 0.52345265 | 0.00132055 | 0.02436804 |
| LTN1 | -0.5477318 | 0.00132093 | 0.02436804 |
| DEF8 | 0.50854913 | 0.00132146 | 0.02436804 |
| KRIT1 | -0.5397303 | 0.00132997 | 0.0244489 |
| TIMM50 | 0.49623132 | 0.00133312 | 0.0244489 |
| BIRC2 | -0.5368058 | 0.00133354 | 0.0244489 |
| HLA-L | 0.65880152 | 0.00133469 | 0.0244489 |
| ERLEC1 | -0.4304477 | 0.00133564 | 0.0244489 |
| SPINT1-AS1 | 1.45331646 | 0.00135033 | 0.02468157 |
| IL27 | 3.77515747 | 0.00135505 | 0.02470081 |
| EHD3 | -3.9537724 | 0.00135534 | 0.02470081 |
| ESCO1 | -0.5488403 | 0.0013575 | 0.02470416 |
| ZFC3H1 | -0.4498644 | 0.0013762 | 0.02500798 |
| AC092747.4 | -1.4228645 | 0.00139001 | 0.0251722 |
| AMDHD2 | 0.56033151 | 0.00139096 | 0.0251722 |
| BAP1 | 0.45861889 | 0.00139268 | 0.0251722 |
| CCDC107 | 0.67583465 | 0.00139488 | 0.0251722 |
| C17orf62 | 0.45278146 | 0.00139532 | 0.0251722 |
| LINC01480 | -1.5197346 | 0.00139825 | 0.02518859 |
| UBTD2 | -0.5070192 | 0.00140273 | 0.02520034 |
| C1orf61 | -2.7835038 | 0.00140487 | 0.02520034 |
| FAM107B | -0.5380421 | 0.00140495 | 0.02520034 |
| INTS1 | 0.46172435 | 0.00140716 | 0.02520366 |
| DGCR2 | 0.52169055 | 0.0014241 | 0.02547056 |
| G6PC3 | 0.55107475 | 0.00142835 | 0.02547987 |
| TPK1 | -0.4681436 | 0.00142877 | 0.02547987 |
| TYROBP | 0.5706435 | 0.00143132 | 0.02547987 |
| IRF2BPL | 0.55197722 | 0.0014342 | 0.02547987 |
| FIGNL1 | -0.8472033 | 0.00143703 | 0.02547987 |
| IRF2BP1 | 0.67530612 | 0.00143716 | 0.02547987 |
| MCOLN1 | 0.44094435 | 0.00144038 | 0.02547987 |
| R3HCC1L | -0.4653257 | 0.00144095 | 0.02547987 |
| LAMTOR1 | 0.46320295 | 0.00145292 | 0.0256553 |
| NORAD | -0.5075133 | 0.00145838 | 0.02571526 |
| NT5C | 0.69514942 | 0.00146121 | 0.02572886 |
| TMTC2 | -1.0581672 | 0.00147173 | 0.02583117 |
| ZNF654 | -0.6623319 | 0.00147684 | 0.02583117 |
| TPMT | -0.488325 | 0.00147805 | 0.02583117 |
| HS2ST1 | -0.5995074 | 0.00147825 | 0.02583117 |
| SUB1 | -0.4587644 | 0.0014805 | 0.02583117 |
| DCTPP1 | 0.50715783 | 0.00148143 | 0.02583117 |
| LRFN4 | 0.56492101 | 0.0014822 | 0.02583117 |
| CLPB | 0.63785077 | 0.00148358 | 0.02583117 |
| KCTD15 | 0.62782502 | 0.00148571 | 0.02583231 |
| CGRRF1 | -0.6732472 | 0.00150333 | 0.02610238 |
| HNRNPA3 | -0.5724157 | 0.00150823 | 0.02615107 |
| C19orf25 | 0.54537298 | 0.00151307 | 0.02618514 |
| XRCC4 | -0.7730729 | 0.00151439 | 0.02618514 |
| ZBTB1 | -0.5309413 | 0.00151968 | 0.02620071 |
| PAPD5 | -0.5732082 | 0.00151993 | 0.02620071 |
| FKBP8 | 0.44321267 | 0.00152159 | 0.02620071 |
| BMS1P1 | 2.53471613 | 0.00153415 | 0.02638054 |
| MTX1 | 0.75463113 | 0.00154955 | 0.02660881 |
| TSPAN33 | 0.46986867 | 0.00155261 | 0.02662467 |
| ABRAXAS1 | -0.9854595 | 0.00156464 | 0.02679412 |
| MYCBP | -0.679114 | 0.00156793 | 0.02680906 |
| GPT | 0.69246329 | 0.00156983 | 0.02680906 |
| STYX | -0.6862006 | 0.00157195 | 0.02680906 |
| CARM1 | 0.45143147 | 0.00159106 | 0.02709698 |
| ATP6V1D | -0.4001987 | 0.00159317 | 0.02709698 |
| RPUSD1 | 0.69421554 | 0.00159937 | 0.02716539 |
| LARP4 | -0.4679668 | 0.00160435 | 0.02716594 |
| ZBTB45 | 0.71478001 | 0.00160444 | 0.02716594 |
| GADD45G | 0.66605966 | 0.0016062 | 0.02716594 |
| LFNG | 0.52836549 | 0.00161025 | 0.02716594 |
| SAMD8 | -0.5592046 | 0.00161028 | 0.02716594 |
| SH3GLB2 | 0.52226364 | 0.00161666 | 0.02723665 |
| SRP19 | -0.7700738 | 0.00162102 | 0.02724811 |
| KIF20B | -1.2158467 | 0.0016217 | 0.02724811 |
| SREBF1 | 0.4374535 | 0.0016374 | 0.0274749 |
| TBC1D15 | -0.5079928 | 0.00164352 | 0.02753191 |
| PITPNM1 | 0.4308927 | 0.00164738 | 0.02753191 |
| ZNF502 | -1.9339499 | 0.00164742 | 0.02753191 |
| CDKN2A | 3.16147263 | 0.00166669 | 0.02781671 |
| DNAJB9 | -0.70647 | 0.00166986 | 0.02783248 |
| LIMS2 | 0.53942371 | 0.00167343 | 0.02785485 |
| ZNF513 | 0.5395253 | 0.00168292 | 0.02797554 |
| NUDT21 | -0.5101441 | 0.00169413 | 0.02801898 |
| AKR7A2 | 0.46106768 | 0.00169669 | 0.02801898 |
| RPS16 | 0.54494828 | 0.00169725 | 0.02801898 |
| CCDC88A | -0.5097345 | 0.00169861 | 0.02801898 |
| DEF6 | 0.54322062 | 0.00169919 | 0.02801898 |
| TMEM201 | 0.80363273 | 0.00169943 | 0.02801898 |
| SFI1 | 0.6165117 | 0.00170125 | 0.02801898 |
| SCRN3 | -0.9539609 | 0.0017132 | 0.02817863 |
| IL18 | -0.6932616 | 0.00172414 | 0.02828293 |
| PPP4R2 | -0.7150742 | 0.0017245 | 0.02828293 |
| GFPT1 | -0.4819093 | 0.00172761 | 0.02828293 |
| UBTD1 | 0.58191482 | 0.00172904 | 0.02828293 |
| DLAT | -0.5095207 | 0.00173238 | 0.02828293 |
| SCAF11 | -0.4348502 | 0.00173499 | 0.02828293 |
| AL031775.1 | -1.6501933 | 0.0017354 | 0.02828293 |
| RPS28 | 0.53652166 | 0.00174286 | 0.02836749 |
| C1orf35 | 0.69169641 | 0.00175436 | 0.02851751 |
| ICK | -0.6114158 | 0.00177711 | 0.02884436 |
| AC011497.2 | 1.30228275 | 0.00177909 | 0.02884436 |
| ZNF467 | 1.00837133 | 0.00178675 | 0.02886257 |
| CEBPD | 0.67249633 | 0.0017893 | 0.02886257 |
| CABP4 | 1.25594267 | 0.00179028 | 0.02886257 |
| TPGS1 | 0.79243413 | 0.00179067 | 0.02886257 |
| SURF1 | 0.47614433 | 0.00179177 | 0.02886257 |
| EVA1B | 0.82084428 | 0.00180695 | 0.02899298 |
| MAFA | 1.13264776 | 0.00180726 | 0.02899298 |
| DNAJC1 | -0.4898501 | 0.00181033 | 0.02899298 |
| C6orf120 | -0.6595394 | 0.00181132 | 0.02899298 |
| C11orf54 | -0.7242708 | 0.00181148 | 0.02899298 |
| HGH1 | 0.63743253 | 0.00182829 | 0.02915222 |
| C11orf58 | -0.4556656 | 0.0018285 | 0.02915222 |
| ZNF468 | -0.556209 | 0.0018299 | 0.02915222 |
| CXCR3 | 2.36486884 | 0.00183156 | 0.02915222 |
| ZKSCAN8 | -0.6553182 | 0.00183311 | 0.02915222 |
| CNEP1R1 | -0.5733574 | 0.00183989 | 0.02919153 |
| PIK3C2A | -0.5100523 | 0.00184265 | 0.02919153 |
| DPP7 | 0.43965802 | 0.00184269 | 0.02919153 |
| ZNF720 | -0.6250676 | 0.0018464 | 0.02919153 |
| PITPNM3 | 1.39104078 | 0.00184727 | 0.02919153 |
| SLC48A1 | 0.51994298 | 0.00185144 | 0.02922046 |
| FAM96B | 0.52382138 | 0.00186492 | 0.02935741 |
| SCOC | -1.3787629 | 0.00186497 | 0.02935741 |
| PNISR | -0.4215305 | 0.00187092 | 0.02935741 |
| NDRG2 | 0.43096854 | 0.00187172 | 0.02935741 |
| TNRC18 | 0.42850057 | 0.00187188 | 0.02935741 |
| ARHGAP4 | 0.44664725 | 0.00188378 | 0.0294569 |
| PNPLA8 | -0.4741441 | 0.00188506 | 0.0294569 |
| YIPF3 | 0.45919291 | 0.0018853 | 0.0294569 |
| TMEM109 | 0.46057147 | 0.00189234 | 0.02952996 |
| DVL1 | 0.54835997 | 0.00189786 | 0.02957911 |
| MT-TT | -1.2646209 | 0.00190169 | 0.02960189 |
| E2F4 | 0.45521374 | 0.00191503 | 0.02977242 |
| AC008124.1 | -0.8756191 | 0.0019227 | 0.02984118 |
| CCAR2 | 0.40774474 | 0.00192513 | 0.02984118 |
| BAG4 | -0.6370353 | 0.00192986 | 0.02984118 |
| TRIP6 | 0.46685328 | 0.00193002 | 0.02984118 |
| SIRT1 | -0.55219 | 0.00193141 | 0.02984118 |
| C7orf49 | 0.52454388 | 0.00193652 | 0.02988326 |
| IGHV2-5 | 2.46661266 | 0.00194309 | 0.02992787 |
| NDUFAF3 | 0.53770189 | 0.00194421 | 0.02992787 |
| ABHD13 | -0.7384394 | 0.00195073 | 0.02999125 |
| TMEM181 | -0.4648758 | 0.00195808 | 0.03004003 |
| RFNG | 0.57215951 | 0.00195871 | 0.03004003 |
| ETNK1 | -0.7412189 | 0.00197871 | 0.03030949 |
| TNFSF12 | 0.52280291 | 0.00198318 | 0.03031683 |
| KIFC3 | 0.43219447 | 0.00198538 | 0.03031683 |
| BLOC1S6 | -0.509961 | 0.00198648 | 0.03031683 |
| AP5Z1 | 0.43035724 | 0.00199846 | 0.03046241 |
| DMTF1 | -0.436633 | 0.00200185 | 0.030477 |
| ATM | -0.4440964 | 0.00200543 | 0.03049432 |
| CXorf40B | 0.65942354 | 0.00201066 | 0.03050639 |
| DYM | -0.4382597 | 0.00201261 | 0.03050639 |
| ARHGEF40 | 0.49863655 | 0.00201356 | 0.03050639 |
| ARHGAP45 | 0.47998548 | 0.00201861 | 0.03054579 |
| CDIPT | 0.57935364 | 0.002025 | 0.03060032 |
| RNPEPL1 | 0.41729131 | 0.00202711 | 0.03060032 |
| UTS2R | 2.40605045 | 0.00203378 | 0.03063751 |
| CORO1A | 0.47811727 | 0.00203448 | 0.03063751 |
| PREB | 0.40909749 | 0.00203902 | 0.03066887 |
| EIF2S1 | -0.4432536 | 0.00206988 | 0.03108209 |
| CUL5 | -0.5916155 | 0.00207148 | 0.03108209 |
| C14orf2 | 0.50084323 | 0.00208864 | 0.03127915 |
| ERCC4 | -0.7339276 | 0.00208981 | 0.03127915 |
| WDR18 | 0.68759114 | 0.00209276 | 0.03127915 |
| MUS81 | 0.4331549 | 0.00209463 | 0.03127915 |
| METTL4 | -0.8892773 | 0.00209945 | 0.0313136 |
| XRCC1 | 0.6328444 | 0.00210318 | 0.03133191 |
| OSGIN2 | -0.5654424 | 0.00211909 | 0.03153123 |
| IER2 | 0.44704836 | 0.00212272 | 0.03154766 |
| KBTBD7 | -0.9858666 | 0.00213522 | 0.03166002 |
| DLD | -0.4295665 | 0.00213568 | 0.03166002 |
| LINC01503 | 1.58247213 | 0.00214001 | 0.03166002 |
| PAPOLA | -0.4756706 | 0.00214207 | 0.03166002 |
| FCHO1 | 0.55942218 | 0.00214296 | 0.03166002 |
| ALDH3B1 | 0.44798123 | 0.00217648 | 0.03211727 |
| TRAF4 | 0.63583884 | 0.00219952 | 0.03241886 |
| RPP25L | 0.85579731 | 0.00220468 | 0.03245665 |
| DCUN1D4 | -0.6543099 | 0.0022093 | 0.03247342 |
| CCNG1 | -0.6977872 | 0.0022155 | 0.03247342 |
| MIER3 | -0.5504518 | 0.00221971 | 0.03247342 |
| GAS2L1 | 0.6090163 | 0.00222098 | 0.03247342 |
| RNASET2 | 0.43617456 | 0.00222158 | 0.03247342 |
| PPIL4 | -0.4597174 | 0.00222266 | 0.03247342 |
| GUK1 | 0.41602285 | 0.00222698 | 0.03247342 |
| AURKAIP1 | 0.52060196 | 0.00223108 | 0.03247342 |
| PIGS | 0.46346481 | 0.00223382 | 0.03247342 |
| MED22 | 0.47861449 | 0.00223729 | 0.03247342 |
| MZT1 | -0.9948175 | 0.00223756 | 0.03247342 |
| NME3 | 0.6551838 | 0.00223931 | 0.03247342 |
| CCNC | -0.7419974 | 0.00224127 | 0.03247342 |
| MAN2C1 | 0.45168756 | 0.00224224 | 0.03247342 |
| EHBP1 | -1.0442776 | 0.00225454 | 0.03247979 |
| ATAD2B | -0.4937344 | 0.00225546 | 0.03247979 |
| STK11 | 0.43244679 | 0.00225724 | 0.03247979 |
| TMEM9 | 0.62168255 | 0.0022632 | 0.03247979 |
| GTF2IRD1 | 0.64214492 | 0.0022649 | 0.03247979 |
| IRF5 | 0.42616235 | 0.00226509 | 0.03247979 |
| TOM1 | 0.42549739 | 0.00226621 | 0.03247979 |
| SIGLEC15 | 0.80393031 | 0.00226721 | 0.03247979 |
| FGFR1OP2 | -0.4571776 | 0.00226748 | 0.03247979 |
| SLC4A7 | -0.5366832 | 0.00226979 | 0.03247979 |
| ZNF703 | 0.94220734 | 0.00227236 | 0.03247979 |
| TOR4A | 0.4554119 | 0.00227703 | 0.03247979 |
| C21orf2 | 0.77495098 | 0.00227925 | 0.03247979 |
| ZYG11B | -0.5982684 | 0.00228025 | 0.03247979 |
| GNAI3 | -0.4514843 | 0.0022817 | 0.03247979 |
| SLC30A4 | -0.5006452 | 0.00229289 | 0.03259775 |
| WASHC4 | -0.5927589 | 0.00229521 | 0.03259775 |
| RNF123 | 0.56585018 | 0.00230616 | 0.03271597 |
| HK3 | 0.41466293 | 0.00231346 | 0.03275482 |
| ATF6B | 0.41944136 | 0.00231414 | 0.03275482 |
| SLC35A2 | 0.53367143 | 0.00231822 | 0.03277536 |
| FAM126A | -0.4298871 | 0.00232708 | 0.0328567 |
| ZNF83 | -0.5766962 | 0.00233048 | 0.0328567 |
| COX6B1 | 0.51809154 | 0.00233187 | 0.0328567 |
| LRIF1 | -0.5440604 | 0.00233646 | 0.03288434 |
| GRK6 | 0.46168402 | 0.00234059 | 0.0329053 |
| EIF3C | -1.2401481 | 0.00234671 | 0.0329543 |
| OSTC | -0.4429663 | 0.00234972 | 0.03295939 |
| TYW5 | -0.7967221 | 0.00235553 | 0.03300391 |
| TMSB4X | 0.53798263 | 0.00236352 | 0.03304285 |
| POP5 | 0.71527241 | 0.00236361 | 0.03304285 |
| FAAP20 | 0.67046882 | 0.00237657 | 0.03305107 |
| TMX1 | -0.4810046 | 0.00237792 | 0.03305107 |
| LLPH | -0.7159318 | 0.00237815 | 0.03305107 |
| GNAQ | -0.6090941 | 0.00238063 | 0.03305107 |
| MPV17L2 | 0.72632464 | 0.00238229 | 0.03305107 |
| CKAP2 | -0.6820523 | 0.00238278 | 0.03305107 |
| ANKS1A | 0.43546057 | 0.00238441 | 0.03305107 |
| TCEA1 | -0.8430936 | 0.00238537 | 0.03305107 |
| UBE2Q2 | -0.616815 | 0.00239825 | 0.03319257 |
| RNF219 | -0.508372 | 0.00240095 | 0.03319318 |
| PTCD1 | 1.20619613 | 0.00241431 | 0.03334096 |
| AL157392.3 | -0.9410056 | 0.00241756 | 0.03334894 |
| RETREG3 | 0.450124 | 0.00242171 | 0.0333693 |
| RB1CC1 | -0.5788654 | 0.0024562 | 0.03380724 |
| SHB | 0.64336387 | 0.00246261 | 0.03385816 |
| SPRED1 | -0.5064033 | 0.00246676 | 0.03385843 |
| GAS2L3 | -0.4697407 | 0.00246805 | 0.03385843 |
| SLC25A35 | 0.8474304 | 0.00248485 | 0.03404296 |
| ELP6 | 0.72142906 | 0.00248696 | 0.03404296 |
| TMEM135 | -0.4962369 | 0.00249436 | 0.03410695 |
| SOWAHC | -0.7860566 | 0.00249782 | 0.03410831 |
| PAK4 | 0.80940337 | 0.00249993 | 0.03410831 |
| RAD18 | -0.8283958 | 0.00251781 | 0.03431481 |
| GBA | 0.39159495 | 0.0025336 | 0.03449239 |
| CST3 | 0.66751836 | 0.00255024 | 0.03468098 |
| FZR1 | 0.44765124 | 0.00256824 | 0.03488788 |
| UGGT2 | -0.6662832 | 0.00257963 | 0.03500449 |
| URM1 | 0.45967553 | 0.00258827 | 0.03508359 |
| TMCO1 | -0.4184636 | 0.00260054 | 0.03521161 |
| RLIM | -0.4730431 | 0.0026123 | 0.03533253 |
| ABCA2 | 0.47370526 | 0.00261674 | 0.03535436 |
| CLDND1 | -0.4839298 | 0.00262502 | 0.03541415 |
| RHOG | 0.49273025 | 0.00263167 | 0.03541415 |
| TBCD | 0.44122096 | 0.00263303 | 0.03541415 |
| SNRPD2 | 0.4501427 | 0.0026375 | 0.03541415 |
| DNASE1L1 | 0.52979037 | 0.00263789 | 0.03541415 |
| RPL22L1 | -0.5769737 | 0.00263834 | 0.03541415 |
| NOC4L | 0.72967793 | 0.00264103 | 0.03541415 |
| KIAA0513 | 0.48295394 | 0.0026468 | 0.03545344 |
| PLIN3 | 0.39775533 | 0.00266062 | 0.03560036 |
| MBOAT7 | 0.46042211 | 0.00267444 | 0.03574702 |
| PNRC2 | -0.4650529 | 0.00269543 | 0.03585728 |
| CIB1 | 0.42589978 | 0.00269584 | 0.03585728 |
| UFM1 | -0.4938958 | 0.00269699 | 0.03585728 |
| PDCD1 | 0.6472343 | 0.00269811 | 0.03585728 |
| RBM27 | -0.6816422 | 0.00269965 | 0.03585728 |
| TCIRG1 | 0.44437506 | 0.00269992 | 0.03585728 |
| ZNF358 | 0.85689023 | 0.00270919 | 0.03591576 |
| PCNP | -0.5202238 | 0.00271008 | 0.03591576 |
| PREPL | -0.4987976 | 0.00271871 | 0.03599197 |
| CD109 | -0.4328504 | 0.00272811 | 0.03607327 |
| ACOT2 | 0.73141413 | 0.00273063 | 0.03607327 |
| SMARCE1 | -0.4623476 | 0.00273463 | 0.03608781 |
| PRPF39 | -0.5963102 | 0.00274056 | 0.03612793 |
| FSTL3 | 2.00628098 | 0.00274371 | 0.03613133 |
| TMEM161A | 0.76289064 | 0.00275021 | 0.03617876 |
| C11orf57 | -0.5020027 | 0.00276425 | 0.03630381 |
| ZNF738 | -1.1688814 | 0.00276553 | 0.03630381 |
| TIE1 | 0.85404065 | 0.00277263 | 0.03635871 |
| LIMK1 | 0.4342733 | 0.00278057 | 0.03642458 |
| LMNTD2 | 1.84176446 | 0.00279538 | 0.03655853 |
| PHOSPHO1 | 0.75787218 | 0.00279665 | 0.03655853 |
| TMEM147 | 0.44644399 | 0.00280579 | 0.03663967 |
| ZNF614 | -0.657341 | 0.0028374 | 0.03701365 |
| NOL3 | 0.87481916 | 0.00285824 | 0.03720713 |
| C12orf4 | -0.4818549 | 0.00285842 | 0.03720713 |
| SH2D1A | -1.8984779 | 0.00286265 | 0.03720713 |
| IFFO1 | 0.47868349 | 0.00286415 | 0.03720713 |
| CCNT2 | -0.4417004 | 0.00287939 | 0.03736627 |
| C2orf49 | -0.5618159 | 0.00289082 | 0.03747565 |
| C8orf37 | -1.4189113 | 0.00290753 | 0.0376531 |
| WDR6 | 0.44152757 | 0.00291403 | 0.03769824 |
| TNPO1 | -0.5828361 | 0.00292958 | 0.03778684 |
| AC021078.1 | -0.6065787 | 0.00293324 | 0.03778684 |
| ZNF140 | -0.4928565 | 0.00293391 | 0.03778684 |
| COMMD10 | -0.6091335 | 0.00293393 | 0.03778684 |
| ICAM3 | 0.46300523 | 0.00293839 | 0.03778684 |
| HERC3 | -0.4232439 | 0.00293904 | 0.03778684 |
| SGO2 | -1.0013004 | 0.00294523 | 0.03782747 |
| B3GLCT | -0.5966096 | 0.00295037 | 0.03785453 |
| KBTBD2 | -0.4560779 | 0.00296482 | 0.03800084 |
| ANKRD36C | -1.58213 | 0.00296937 | 0.03802012 |
| HDHD5 | 0.49283604 | 0.0029764 | 0.03807109 |
| DHX34 | 0.48887845 | 0.00300968 | 0.03840826 |
| DEAF1 | 0.59043739 | 0.00301285 | 0.03840826 |
| ATRX | -0.5531001 | 0.00301312 | 0.03840826 |
| USP45 | -0.8665633 | 0.00301507 | 0.03840826 |
| TTC31 | 0.50731629 | 0.00302331 | 0.03842284 |
| NDRG1 | 0.49221973 | 0.00302366 | 0.03842284 |
| KCNN4 | 0.49360332 | 0.00302544 | 0.03842284 |
| ARMC1 | -0.7578761 | 0.00303227 | 0.03847038 |
| XIST | 5.68490247 | 0.00304309 | 0.03856841 |
| PTRHD1 | 0.503472 | 0.00304675 | 0.03857563 |
| PHAX | -0.4449802 | 0.00305426 | 0.03863156 |
| SIVA1 | 0.57857779 | 0.0030603 | 0.03866886 |
| SMYD5 | 0.65875193 | 0.00306382 | 0.03867411 |
| RPL36 | 0.45239098 | 0.00306992 | 0.03871205 |
| ZSCAN16-AS1 | 1.25266653 | 0.00308669 | 0.03888414 |
| PMVK | 0.48610446 | 0.00310557 | 0.03908255 |
| MBNL1-AS1 | 1.34823844 | 0.00311571 | 0.03914613 |
| NFKBIL1 | 0.64392048 | 0.00311689 | 0.03914613 |
| RNF44 | 0.49496632 | 0.00312286 | 0.03918165 |
| CTDSPL2 | -0.5230063 | 0.00313546 | 0.0393003 |
| SLC37A2 | 0.40697221 | 0.00314938 | 0.03940964 |
| C1S | -2.0804385 | 0.00315276 | 0.03940964 |
| WDR3 | -0.5234144 | 0.00315365 | 0.03940964 |
| MPP5 | -0.5455824 | 0.00316826 | 0.03955261 |
| CDCP1 | 0.46308384 | 0.00317915 | 0.03963463 |
| SLC16A5 | 0.92376114 | 0.00318251 | 0.03963463 |
| MTPAP | -0.5128027 | 0.00318436 | 0.03963463 |
| CHURC1 | -0.4858759 | 0.00319837 | 0.03973456 |
| TRIOBP | 0.41249833 | 0.00319875 | 0.03973456 |
| MDGA1 | 2.67253044 | 0.00321012 | 0.03980072 |
| EML3 | 0.42601634 | 0.00321614 | 0.03980072 |
| GPN3 | -0.8826346 | 0.00321617 | 0.03980072 |
| LMBR1L | 0.46601126 | 0.00321683 | 0.03980072 |
| RBM7 | -0.6840395 | 0.00322979 | 0.03989312 |
| NFE2 | 0.78758527 | 0.00323069 | 0.03989312 |
| PNPLA6 | 0.43263436 | 0.0032385 | 0.03995003 |
| TRMT2A | 0.42492519 | 0.00324919 | 0.04004233 |
| RRP12 | 0.57929265 | 0.00328322 | 0.04042183 |
| ZNF836 | -0.8862275 | 0.00329221 | 0.0404925 |
| PER1 | 0.67886594 | 0.00330069 | 0.04055693 |
| RPL38 | 0.5228279 | 0.00331153 | 0.0406501 |
| HMG20A | -0.449456 | 0.0033289 | 0.04075575 |
| EIF6 | 0.4585522 | 0.00332897 | 0.04075575 |
| PRR14 | 0.42614173 | 0.00333102 | 0.04075575 |
| RSBN1 | -0.7207836 | 0.00333449 | 0.04075575 |
| BRMS1L | -1.0659675 | 0.00333646 | 0.04075575 |
| MLXIP | 0.45352421 | 0.00334584 | 0.04083041 |
| AP1M1 | 0.38673001 | 0.0033584 | 0.04094216 |
| RHEB | -0.4401602 | 0.00336156 | 0.04094216 |
| DDX52 | -0.4495722 | 0.00338062 | 0.04113415 |
| GPATCH2L | -0.4093493 | 0.00338728 | 0.04114036 |
| NAP1L1 | -0.4302043 | 0.00339498 | 0.04114036 |
| THOC6 | 0.47031927 | 0.00339573 | 0.04114036 |
| AGPAT2 | 0.42703835 | 0.00339876 | 0.04114036 |
| TLK1 | -0.4849242 | 0.00339958 | 0.04114036 |
| HMGN2 | 0.58528018 | 0.0034009 | 0.04114036 |
| PELI3 | 0.75390128 | 0.00341051 | 0.04119798 |
| CLUH | 0.39399272 | 0.00341294 | 0.04119798 |
| B3GALT4 | 0.64406738 | 0.00341557 | 0.04119798 |
| MEPCE | 0.43863868 | 0.00342218 | 0.0412161 |
| CWC22 | -0.5082139 | 0.00342367 | 0.0412161 |
| CLCN3 | -0.4267045 | 0.00344069 | 0.04133611 |
| CAPN15 | 0.46946059 | 0.003442 | 0.04133611 |
| FAM69A | -0.4781742 | 0.0034454 | 0.04133611 |
| BAIAP2-AS1 | 0.50619533 | 0.00344688 | 0.04133611 |
| SCAMP3 | 0.51858732 | 0.00345076 | 0.04134294 |
| CISD3 | 0.59081468 | 0.00345481 | 0.04135172 |
| LYPLA1 | -0.8589708 | 0.00346172 | 0.0413948 |
| HRH2 | 0.43711297 | 0.00346774 | 0.04142708 |
| YIPF6 | -0.4443383 | 0.00348622 | 0.04156214 |
| UBA6 | -0.5442897 | 0.00348737 | 0.04156214 |
| TFEB | 0.50334688 | 0.00348904 | 0.04156214 |
| BTBD2 | 0.55810513 | 0.00350168 | 0.04167304 |
| MAFB | 0.43113772 | 0.00351029 | 0.04172709 |
| RFC3 | -0.8743879 | 0.00351291 | 0.04172709 |
| IMPAD1 | -0.496775 | 0.00352542 | 0.0418359 |
| EEF1AKMT2 | -1.0913683 | 0.00352926 | 0.04184162 |
| LYPLA2 | 0.49768621 | 0.00353558 | 0.04185058 |
| CDC37L1 | -0.6243271 | 0.00353672 | 0.04185058 |
| TSKU | 0.61429883 | 0.00356051 | 0.04209219 |
| BOP1 | 0.57879902 | 0.00358268 | 0.04231421 |
| LTBP4 | 0.73257465 | 0.00358873 | 0.0423457 |
| NEDD8 | 0.42784098 | 0.00359688 | 0.04240179 |
| SLC25A33 | -0.7042833 | 0.00360244 | 0.04242724 |
| EPB41L1 | 0.62172522 | 0.00361561 | 0.04254178 |
| ERCC8 | -0.7001276 | 0.00362453 | 0.04254178 |
| ZNHIT6 | -0.6601235 | 0.00362479 | 0.04254178 |
| NAPRT | 0.53977727 | 0.00362852 | 0.04254178 |
| MBIP | -0.6538046 | 0.00363178 | 0.04254178 |
| AL138899.1 | -0.8010461 | 0.00363261 | 0.04254178 |
| PDAP1 | 0.44442198 | 0.00364284 | 0.04262159 |
| FARSB | -0.4825476 | 0.00365962 | 0.04276777 |
| CETN3 | -0.8124229 | 0.00366574 | 0.04276777 |
| HTRA2 | 0.65208641 | 0.00366614 | 0.04276777 |
| VSTM2L | 2.43955511 | 0.00366904 | 0.04276777 |
| DDX28 | 0.55897368 | 0.00368007 | 0.04285629 |
| SPCS3 | -0.8097264 | 0.00369148 | 0.0429492 |
| NAA16 | -0.7423525 | 0.00370352 | 0.04304916 |
| TBC1D23 | -0.5739114 | 0.00371339 | 0.04310064 |
| FBXO41 | 0.56651943 | 0.00371486 | 0.04310064 |
| C2orf76 | -0.7786662 | 0.00372943 | 0.0431693 |
| NAA25 | -0.4150399 | 0.00373002 | 0.0431693 |
| RPS5 | 0.43704865 | 0.00373725 | 0.0431693 |
| SLC25A11 | 0.43944211 | 0.00373876 | 0.0431693 |
| TMEM102 | 0.54608203 | 0.00374136 | 0.0431693 |
| ARHGEF12 | -0.4543294 | 0.0037464 | 0.0431693 |
| ACTR10 | -0.4008272 | 0.0037479 | 0.0431693 |
| EBP | 0.43475965 | 0.00374844 | 0.0431693 |
| BANP | 0.51961806 | 0.00375513 | 0.04320654 |
| ELK4 | -0.5346643 | 0.00376343 | 0.04326213 |
| R3HDM4 | 0.41274458 | 0.00377171 | 0.04331736 |
| NOP14-AS1 | 0.82243918 | 0.00377929 | 0.04336453 |
| RRP15 | -0.636366 | 0.00379957 | 0.04355726 |
| TSPAN14 | 0.38312177 | 0.00380991 | 0.04361896 |
| AP1S1 | 0.48105348 | 0.00381406 | 0.04361896 |
| FPGS | 0.47644236 | 0.00381614 | 0.04361896 |
| ZC3H15 | -0.4121333 | 0.00381893 | 0.04361896 |
| ELP5 | 0.51963808 | 0.00382824 | 0.04366471 |
| ENG | 0.40935214 | 0.00383111 | 0.04366471 |
| CDR2L | 0.49203125 | 0.00383343 | 0.04366471 |
| ZNF600 | -0.7652447 | 0.00384081 | 0.04370894 |
| AC011603.2 | 1.65727948 | 0.00385385 | 0.0438174 |
| GNG5 | 0.39700888 | 0.00386209 | 0.04387109 |
| AC125257.1 | -1.112865 | 0.00386846 | 0.04390354 |
| UHRF1BP1L | -0.4663253 | 0.00389472 | 0.04416139 |
| SIGIRR | 0.718676 | 0.00389867 | 0.04416605 |
| EPM2AIP1 | -0.4955004 | 0.00391381 | 0.04426523 |
| BRWD1 | -0.543254 | 0.00391654 | 0.04426523 |
| NDUFS6 | 0.50518599 | 0.00391806 | 0.04426523 |
| EPHB6 | 0.62126125 | 0.003938 | 0.04445028 |
| ZNF420 | -0.7545946 | 0.00394576 | 0.04449766 |
| SRSF10 | -0.5544514 | 0.00397282 | 0.04472439 |
| AC083799.1 | -0.9901727 | 0.00397303 | 0.04472439 |
| SLC35A4 | 0.41374992 | 0.00398278 | 0.04478275 |
| FBXL17 | -0.5822431 | 0.00398539 | 0.04478275 |
| HLA-DQB2 | 0.4518984 | 0.00399416 | 0.04481557 |
| SLC25A26 | 0.60554044 | 0.00399549 | 0.04481557 |
| CYCS | -0.6309676 | 0.00400874 | 0.04492374 |
| EIF3M | -0.37802 | 0.00401262 | 0.04492697 |
| CCDC126 | -0.8376455 | 0.00404134 | 0.04516888 |
| TGFB1 | 0.43915299 | 0.00404146 | 0.04516888 |
| ARHGAP9 | 0.44497739 | 0.00404822 | 0.0452039 |
| MSRB2 | 1.1492994 | 0.00406362 | 0.0453353 |
| FAM58A | 0.52726382 | 0.00408451 | 0.04552766 |
| RPS23 | 0.42936135 | 0.00409372 | 0.04555066 |
| SNX12 | 0.384977 | 0.00409622 | 0.04555066 |
| FAM110B | 0.61052772 | 0.00409752 | 0.04555066 |
| NIPAL1 | -1.1022404 | 0.00410379 | 0.04557978 |
| SRP54 | -0.39391 | 0.00411039 | 0.04561257 |
| ALKBH5 | 0.3696686 | 0.00412092 | 0.04567711 |
| IDH3A | -0.4574099 | 0.00412422 | 0.04567711 |
| SPAST | -0.6053109 | 0.00412719 | 0.04567711 |
| ZNF865 | 0.66580075 | 0.00413639 | 0.04573838 |
| LYRM7 | -0.8326154 | 0.0041452 | 0.0457474 |
| GPALPP1 | -0.6071159 | 0.0041484 | 0.0457474 |
| ROR2 | 1.41251897 | 0.00414862 | 0.0457474 |
| LY75 | -0.6592826 | 0.00415186 | 0.0457474 |
| FAM208B | -0.4374062 | 0.00416575 | 0.04582562 |
| TMC6 | 0.40573832 | 0.00417023 | 0.04582562 |
| MED21 | -0.5703706 | 0.00417045 | 0.04582562 |
| CHMP5 | -0.4070248 | 0.00417364 | 0.04582562 |
| ENTPD6 | 0.3830347 | 0.00418073 | 0.04583459 |
| C19orf47 | 0.66933123 | 0.0041818 | 0.04583459 |
| DUSP4 | 0.92783724 | 0.0041875 | 0.0458513 |
| VPS51 | 0.51614447 | 0.00419067 | 0.0458513 |
| PPP4R3A | -0.414049 | 0.00419956 | 0.0459083 |
| COX5A | 0.382995 | 0.00421752 | 0.04606435 |
| EMILIN1 | 1.06024574 | 0.00422497 | 0.04609058 |
| RDX | -0.4350093 | 0.00423165 | 0.04609058 |
| SEC23IP | -0.3792829 | 0.00423593 | 0.04609058 |
| BRIX1 | -0.5821905 | 0.00423742 | 0.04609058 |
| ATP6V1F | 0.42044608 | 0.00423959 | 0.04609058 |
| CD55 | -0.4299912 | 0.00424237 | 0.04609058 |
| FAM13B | -0.4055216 | 0.00424708 | 0.04609058 |
| PSMA3-AS1 | -0.4433872 | 0.00425001 | 0.04609058 |
| FXYD6 | 0.92556151 | 0.00425315 | 0.04609058 |
| PARVG | 0.4156822 | 0.0042593 | 0.04610256 |
| AC000123.3 | -1.1137655 | 0.00426164 | 0.04610256 |
| UPRT | -0.5450709 | 0.00427108 | 0.04616467 |
| SASS6 | -1.3579117 | 0.00429932 | 0.04642967 |
| ZFYVE19 | 0.61209356 | 0.00430654 | 0.04644461 |
| WBP2 | 0.41707506 | 0.00431414 | 0.04644461 |
| PIK3CA | -0.47908 | 0.00432218 | 0.04644461 |
| GFER | 0.64075952 | 0.00432251 | 0.04644461 |
| UBA52 | 0.46977629 | 0.0043238 | 0.04644461 |
| CCT6A | -0.365947 | 0.00432624 | 0.04644461 |
| NT5DC2 | 0.44679952 | 0.00432863 | 0.04644461 |
| PRPF38B | -0.3931673 | 0.00433047 | 0.04644461 |
| PAQR4 | 0.51205364 | 0.00433851 | 0.04645351 |
| TMEM170A | -0.5326373 | 0.00433874 | 0.04645351 |
| LIN7C | -0.7303742 | 0.00434898 | 0.04652323 |
| CSNK1G2 | 0.40265104 | 0.00435388 | 0.04653587 |
| ANAPC4 | -0.5320294 | 0.00436462 | 0.04660061 |
| CORO1B | 0.37692399 | 0.00436741 | 0.04660061 |
| NDFIP2 | -0.6700291 | 0.00437506 | 0.04664237 |
| FLOT2 | 0.38585554 | 0.00439136 | 0.04674202 |
| GLMN | -1.017997 | 0.00439189 | 0.04674202 |
| OPRL1 | 0.77428786 | 0.00440546 | 0.04683286 |
| ARRDC2 | 0.42883931 | 0.00440793 | 0.04683286 |
| RBBP5 | -0.4419609 | 0.00442567 | 0.04694267 |
| EIF3F | 0.46472492 | 0.00442579 | 0.04694267 |
| TCF3 | 0.42067758 | 0.00446593 | 0.04731275 |
| G2E3 | -0.6958051 | 0.00446826 | 0.04731275 |
| GUF1 | -0.5628465 | 0.00447207 | 0.04731296 |
| DOK3 | 0.55508201 | 0.00448143 | 0.04736595 |
| OSBPL8 | -0.5554837 | 0.00448466 | 0.04736595 |
| CCDC124 | 0.47061436 | 0.00449455 | 0.04740957 |
| NDUFS8 | 0.50531151 | 0.00449639 | 0.04740957 |
| C14orf159 | 0.45015706 | 0.00452168 | 0.04763595 |
| TMEM167B | -0.399923 | 0.00454204 | 0.04781017 |
| SUV39H2 | -1.0412155 | 0.00455258 | 0.04788072 |
| EIF2S3 | -0.4096094 | 0.00456492 | 0.04797012 |
| SNHG8 | 0.58330766 | 0.00458157 | 0.04810461 |
| NDUFV1 | 0.39300395 | 0.00459357 | 0.04819005 |
| SRSF11 | -0.4486564 | 0.00462103 | 0.04843739 |
| C3orf38 | -0.454617 | 0.00463187 | 0.04845408 |
| KDM8 | 0.82371051 | 0.00463509 | 0.04845408 |
| ZNF765 | -0.588342 | 0.0046385 | 0.04845408 |
| MAP3K2 | -0.436883 | 0.00464401 | 0.04845408 |
| EFHD2 | 0.45772065 | 0.00464583 | 0.04845408 |
| USO1 | -0.4322356 | 0.00464591 | 0.04845408 |
| GORASP1 | 0.37954879 | 0.00465329 | 0.04849055 |
| ITFG1 | -0.4377422 | 0.00466001 | 0.04849675 |
| CYB561D2 | 0.54886217 | 0.00466166 | 0.04849675 |
| SNRPB | 0.37497688 | 0.00467937 | 0.04864056 |
| GGCT | -0.4577698 | 0.00468422 | 0.04865041 |
| MITD1 | -0.5602611 | 0.00469501 | 0.04872194 |
| FBXO11 | -0.4232106 | 0.00470597 | 0.04879515 |
| H2AFX | 0.51878547 | 0.0047343 | 0.04904816 |
| RAB18 | -0.5754381 | 0.00474293 | 0.04909679 |
| DYNLRB1 | 0.45000392 | 0.00477372 | 0.04937458 |
| ENDOG | 0.77922813 | 0.00478087 | 0.04940763 |
| ZNF35 | -0.5737628 | 0.0048143 | 0.04971195 |
| APAF1 | -0.4763382 | 0.00483343 | 0.04986826 |

B)

| α2,3sia + LPS genes | logFC | PValue | FDR |
| --- | --- | --- | --- |
| PHF19 | -1.2210177 | 2.19E-13 | 2.73E-09 |
| MSMO1 | 1.53637468 | 5.42E-13 | 3.38E-09 |
| IDI1 | 0.95719067 | 3.70E-09 | 1.54E-05 |
| HIGD1A | 1.16309559 | 9.93E-09 | 3.10E-05 |
| RNF11 | 0.95115921 | 1.41E-08 | 3.52E-05 |
| ZWILCH | 1.20262586 | 2.57E-08 | 5.24E-05 |
| TWF1 | 1.10702487 | 2.94E-08 | 5.24E-05 |
| DNAJB4 | 1.05071484 | 7.39E-08 | 0.00010214 |
| PSMA1 | 1.10858728 | 7.86E-08 | 0.00010214 |
| SC5D | 1.2058093 | 8.18E-08 | 0.00010214 |
| BMT2 | 1.19796226 | 1.09E-07 | 0.00012399 |
| PPP3R1 | 0.99330602 | 1.70E-07 | 0.00017722 |
| LY96 | 1.01971432 | 2.22E-07 | 0.00021292 |
| GOLT1B | 1.59688993 | 4.69E-07 | 0.0004182 |
| ZBTB41 | 1.47380137 | 7.18E-07 | 0.00059764 |
| FOPNL | 0.95323413 | 1.21E-06 | 0.00094181 |
| MTND2P28 | 0.81029309 | 1.28E-06 | 0.0009428 |
| COPS2 | 0.84925601 | 1.41E-06 | 0.00097446 |
| EIF1AX | 0.91562912 | 1.62E-06 | 0.00106442 |
| DCUN1D1 | 0.92888635 | 2.28E-06 | 0.00137969 |
| STARD4 | 1.18280222 | 2.32E-06 | 0.00137969 |
| MT-TT | 1.61545421 | 2.97E-06 | 0.00168556 |
| ACTR6 | 1.14833697 | 3.13E-06 | 0.00169725 |
| TXNDC9 | 0.8741167 | 3.87E-06 | 0.00201516 |
| CUL5 | 0.91436878 | 5.48E-06 | 0.00273661 |
| LY75 | 1.12322229 | 5.82E-06 | 0.00274274 |
| SLC25A24 | 0.71245925 | 5.93E-06 | 0.00274274 |
| DCUN1D4 | 1.22316835 | 6.30E-06 | 0.00280756 |
| PSMD5 | -0.6964515 | 6.95E-06 | 0.0029911 |
| FBXO30 | 0.75363678 | 9.51E-06 | 0.0036761 |
| ORMDL1 | 0.66609302 | 9.72E-06 | 0.0036761 |
| MDM2 | 0.64403984 | 9.96E-06 | 0.0036761 |
| PNRC2 | 0.70093662 | 1.00E-05 | 0.0036761 |
| AL671883.3 | -2.8169475 | 1.03E-05 | 0.0036761 |
| BRCA2 | 1.08491331 | 1.04E-05 | 0.0036761 |
| BORCS7 | 1.41282701 | 1.07E-05 | 0.0036761 |
| SLC25A40 | 0.99286494 | 1.09E-05 | 0.0036761 |
| TXNL1 | 0.62838395 | 1.12E-05 | 0.0036761 |
| ZNF292 | 0.81563206 | 1.16E-05 | 0.00371012 |
| HNRNPA3 | 0.80827123 | 1.19E-05 | 0.00371216 |
| SEC61G | 0.80788067 | 1.25E-05 | 0.00381231 |
| YIPF6 | 0.70024886 | 1.39E-05 | 0.00414289 |
| AMZ1 | 3.45918046 | 1.49E-05 | 0.00433895 |
| TPD52 | 1.64450172 | 1.55E-05 | 0.00439381 |
| MOB4 | 1.13091214 | 1.73E-05 | 0.00480645 |
| ZBTB1 | 0.75040227 | 1.89E-05 | 0.00512325 |
| COQ10B | 0.89181252 | 2.00E-05 | 0.00531672 |
| ARL4A | 1.30540046 | 2.34E-05 | 0.00609639 |
| VPS26A | 0.61586485 | 2.47E-05 | 0.00620614 |
| STYX | 1.02314767 | 2.49E-05 | 0.00620614 |
| NAA50 | 1.00541238 | 2.54E-05 | 0.00621854 |
| TMEM41B | 0.8959914 | 2.70E-05 | 0.00648112 |
| P4HTM | -0.9881597 | 2.78E-05 | 0.00652821 |
| DNAJB9 | 0.95478764 | 2.82E-05 | 0.00652821 |
| C5orf24 | 0.93118803 | 3.11E-05 | 0.00692818 |
| ENGASE | -0.7448363 | 3.16E-05 | 0.00692818 |
| CLDND1 | 0.70398184 | 3.16E-05 | 0.00692818 |
| CDK6 | 0.68608422 | 3.23E-05 | 0.0069469 |
| ZNF770 | 1.13242321 | 3.53E-05 | 0.0074666 |
| CAMSAP2 | 0.78955251 | 3.80E-05 | 0.0079003 |
| ABHD13 | 1.03378658 | 3.98E-05 | 0.00814173 |
| C14orf159 | -0.7327151 | 4.13E-05 | 0.00831497 |
| FP565260.7 | 3.23522655 | 4.30E-05 | 0.00852537 |
| IRAK3 | 0.70298647 | 4.53E-05 | 0.00884356 |
| CBX3 | 0.63011304 | 4.64E-05 | 0.00886708 |
| AHR | 0.70982705 | 4.69E-05 | 0.00886708 |
| RBM7 | 0.97778357 | 5.15E-05 | 0.00959814 |
| XPO1 | 0.7214203 | 5.35E-05 | 0.00982381 |
| VBP1 | 0.74492812 | 5.68E-05 | 0.0102728 |
| UMAD1 | 1.16196771 | 5.91E-05 | 0.01043349 |
| SYPL1 | 0.90289306 | 5.93E-05 | 0.01043349 |
| KBTBD8 | 0.81366516 | 6.18E-05 | 0.01053566 |
| RCN1 | 0.74784325 | 6.19E-05 | 0.01053566 |
| MCTS1 | 0.83662092 | 6.55E-05 | 0.01053566 |
| MAD1L1 | -0.743511 | 6.59E-05 | 0.01053566 |
| CCPG1 | 1.29779113 | 6.63E-05 | 0.01053566 |
| PCNP | 0.72839785 | 6.63E-05 | 0.01053566 |
| CDC42SE2 | 0.63038023 | 6.66E-05 | 0.01053566 |
| ZBTB10 | 1.07048241 | 6.67E-05 | 0.01053566 |
| FAM102B | 0.66193463 | 7.32E-05 | 0.01142248 |
| RRAGC | 0.6909103 | 7.70E-05 | 0.01187163 |
| CLN3 | -0.9392329 | 8.08E-05 | 0.01229561 |
| PSMA2 | 0.86424612 | 8.41E-05 | 0.01264381 |
| ITGA11 | -1.488114 | 8.82E-05 | 0.01311109 |
| TM9SF3 | 0.90443485 | 8.94E-05 | 0.01312648 |
| PTGES3 | 0.82801213 | 9.23E-05 | 0.01339219 |
| MFAP3 | 0.79645669 | 9.39E-05 | 0.01347322 |
| MB21D2 | 0.7956774 | 9.72E-05 | 0.01378654 |
| TDP2 | 0.54195702 | 0.00010228 | 0.01434646 |
| DGKH | 0.79757757 | 0.00010367 | 0.01438011 |
| RAP2C | 0.90064957 | 0.00010651 | 0.01461124 |
| CCL1 | -2.9153535 | 0.00011085 | 0.01501956 |
| STN1 | -1.5146889 | 0.0001124 | 0.01501956 |
| C9orf72 | 0.70618593 | 0.00011309 | 0.01501956 |
| C2orf69 | 1.18351513 | 0.00011891 | 0.01546361 |
| PTGER2 | 0.67634838 | 0.00011891 | 0.01546361 |
| POC1B | 0.83964346 | 0.00012046 | 0.01550348 |
| SRP19 | 0.98758738 | 0.0001252 | 0.0159495 |
| HES4 | -2.3862469 | 0.0001273 | 0.01604815 |
| Z83843.1 | 1.7226498 | 0.00012917 | 0.01604815 |
| IL27 | -3.9180558 | 0.00012984 | 0.01604815 |
| GALNT18 | -0.5393568 | 0.00013439 | 0.01644883 |
| RCHY1 | 0.88418765 | 0.00013658 | 0.01655384 |
| TMEM170A | 0.76241754 | 0.00014019 | 0.01671066 |
| TAF13 | 0.92383111 | 0.0001428 | 0.01671066 |
| SEH1L | 0.58114151 | 0.00014363 | 0.01671066 |
| MIER1 | 0.64077825 | 0.00014374 | 0.01671066 |
| VPS13A | 0.84106043 | 0.00014457 | 0.01671066 |
| NAA15 | 0.67149152 | 0.00014795 | 0.01694528 |
| LYSMD2 | 0.77831229 | 0.00015186 | 0.01723436 |
| SLC29A1 | -0.9866761 | 0.00015598 | 0.01752236 |
| IFNL1 | -3.4445822 | 0.00015918 | 0.01752236 |
| TRMT10C | 0.77061757 | 0.00015982 | 0.01752236 |
| UBALD2 | -0.6605236 | 0.00016058 | 0.01752236 |
| REEP3 | 0.80240864 | 0.00016141 | 0.01752236 |
| ANP32E | 0.75690827 | 0.00016286 | 0.01752727 |
| HINT3 | 0.73784759 | 0.00016442 | 0.01754348 |
| PDCD1LG2 | 0.59668028 | 0.00016773 | 0.01770573 |
| MAN2A1 | 0.62285534 | 0.00016877 | 0.01770573 |
| EPM2AIP1 | 0.67577785 | 0.0001721 | 0.01790417 |
| MBNL1-AS1 | -1.814299 | 0.00017517 | 0.01807311 |
| PTP4A1 | 0.66164845 | 0.00017769 | 0.01818252 |
| RAP1B | 0.63793364 | 0.00017996 | 0.01823542 |
| TIPRL | 0.60354897 | 0.00018113 | 0.01823542 |
| C16orf72 | 0.64765355 | 0.00018302 | 0.01827862 |
| SMARCE1 | 0.64117227 | 0.00020162 | 0.01997663 |
| SNRPD2 | -0.5759859 | 0.00020502 | 0.02015373 |
| OSTM1 | 0.64208775 | 0.00020687 | 0.02017608 |
| ZBTB26 | 1.19914945 | 0.00021042 | 0.02036371 |
| ZNF430 | 0.74582514 | 0.00021295 | 0.02044985 |
| LIG4 | 0.75996207 | 0.00022101 | 0.02106211 |
| CGRRF1 | 0.94327637 | 0.0002247 | 0.02125152 |
| UBE2O | -0.7910797 | 0.00023048 | 0.02155008 |
| HRH2 | -0.5744689 | 0.00023134 | 0.02155008 |
| GBP5 | -1.0090317 | 0.00023304 | 0.02155008 |
| PSMD12 | 0.69674755 | 0.00023774 | 0.02170159 |
| RAB22A | 0.6306643 | 0.00024062 | 0.02170159 |
| NDFIP2 | 1.02359173 | 0.0002412 | 0.02170159 |
| GOLGA8A | 0.78656278 | 0.00024163 | 0.02170159 |
| BROX | 0.69189895 | 0.00024472 | 0.02182197 |
| ETFRF1 | 1.46913755 | 0.0002469 | 0.02186063 |
| RC3H1 | 0.61273297 | 0.00025498 | 0.02241659 |
| GAS2L3 | 0.71360104 | 0.0002622 | 0.0228901 |
| SNAP23 | 0.59194826 | 0.00027095 | 0.02348985 |
| CBLL1 | 0.62823605 | 0.00028713 | 0.02455498 |
| SLC36A4 | 0.79552562 | 0.0002881 | 0.02455498 |
| RAB18 | 0.75398819 | 0.00028914 | 0.02455498 |
| TSNAX | 0.80973698 | 0.00030906 | 0.02607003 |
| PAXBP1 | 0.6877647 | 0.00031367 | 0.02611515 |
| AC006064.4 | -2.9352607 | 0.00031378 | 0.02611515 |
| TANGO2 | -0.6894208 | 0.00031684 | 0.02619501 |
| RAP2C-AS1 | 1.65732346 | 0.00031972 | 0.02622724 |
| SNAI1 | -2.4909556 | 0.00032143 | 0.02622724 |
| XIAP | 0.58444618 | 0.00032677 | 0.02648955 |
| MICAL2 | -0.5875693 | 0.000339 | 0.02716148 |
| ID2 | 0.5678119 | 0.00033941 | 0.02716148 |
| CYCS | 0.79711202 | 0.00034579 | 0.02735833 |
| RAPGEF6 | 1.29141615 | 0.00034625 | 0.02735833 |
| VIM-AS1 | -0.9767961 | 0.00035147 | 0.02742559 |
| TMEM242 | -1.1392314 | 0.0003515 | 0.02742559 |
| UBE2A | 0.53062471 | 0.00035698 | 0.02755135 |
| CEP135 | 0.72181969 | 0.00035913 | 0.02755135 |
| ZNF518A | 0.74562122 | 0.00035973 | 0.02755135 |
| SRSF3 | 0.58232541 | 0.0003667 | 0.02766903 |
| HPCAL1 | -0.5397768 | 0.00036728 | 0.02766903 |
| SLC41A2 | 0.60907655 | 0.00036792 | 0.02766903 |
| ENO2 | -1.448024 | 0.00037334 | 0.0277916 |
| INTS6 | 0.57051062 | 0.00037522 | 0.0277916 |
| DCTN4 | 0.53048841 | 0.00037855 | 0.0277916 |
| B3GNT5 | 0.76774139 | 0.00038093 | 0.0277916 |
| PPP1R35 | -1.0600566 | 0.0003824 | 0.0277916 |
| GCLM | 0.65018367 | 0.0003829 | 0.0277916 |
| SLC26A2 | 0.65946258 | 0.00039202 | 0.0282892 |
| CREM | 0.60021102 | 0.00039563 | 0.02838516 |
| POGLUT1 | 0.64416317 | 0.00040003 | 0.0285372 |
| TMEM165 | 0.57846483 | 0.00040998 | 0.0287437 |
| SCML1 | 0.91340667 | 0.00041129 | 0.0287437 |
| NUS1 | 0.62097287 | 0.00041398 | 0.0287437 |
| GABARAP | -0.9581159 | 0.00041425 | 0.0287437 |
| OSTC | 0.54986162 | 0.00041444 | 0.0287437 |
| WDR70 | -0.759559 | 0.00041966 | 0.02894525 |
| DPH3 | 0.54050091 | 0.00042608 | 0.02911553 |
| LACC1 | 1.15872942 | 0.0004268 | 0.02911553 |
| GPN3 | 1.19657651 | 0.00043028 | 0.02919338 |
| NOP9 | -0.6080792 | 0.00043344 | 0.02924884 |
| AZIN1 | 0.55311192 | 0.00043994 | 0.02952831 |
| ATPIF1 | -0.612864 | 0.00044646 | 0.02962698 |
| SGTB | 1.15937937 | 0.00045023 | 0.02962698 |
| DR1 | 0.5818552 | 0.00045069 | 0.02962698 |
| DNAJC10 | 0.5282874 | 0.00045091 | 0.02962698 |
| WDR18 | -0.9600813 | 0.00046025 | 0.03003888 |
| NCBP2 | 0.52172842 | 0.00046199 | 0.03003888 |
| NCK1 | 0.53500915 | 0.00048154 | 0.03099583 |
| NDUFC2 | 1.18170201 | 0.00048344 | 0.03099583 |
| ASB7 | 0.88451794 | 0.00048415 | 0.03099583 |
| ARMCX3 | 0.58309793 | 0.00048948 | 0.03117677 |
| SLC25A16 | 0.90690578 | 0.00049341 | 0.03126773 |
| RNF216P1 | -0.7272781 | 0.0004962 | 0.03128582 |
| SLC30A7 | 0.85440675 | 0.00050274 | 0.03153872 |
| MAP4K5 | 0.65781841 | 0.00051026 | 0.03185066 |
| UFM1 | 0.58162395 | 0.00051458 | 0.03185889 |
| RBM27 | 0.85713621 | 0.0005155 | 0.03185889 |
| SLC30A5 | 0.6745718 | 0.000523 | 0.03203775 |
| AC067931.2 | -2.2975926 | 0.00052353 | 0.03203775 |
| ARRDC3 | 0.99824148 | 0.00052795 | 0.03205051 |
| GLS | 0.67759101 | 0.00052887 | 0.03205051 |
| C2orf76 | 0.97353815 | 0.00054141 | 0.03250736 |
| HDHD2 | 1.5695082 | 0.00054162 | 0.03250736 |
| GPR180 | 0.89252847 | 0.00057693 | 0.0344611 |
| LCORL | 0.98424066 | 0.00058183 | 0.03449617 |
| ZNF267 | 0.7331927 | 0.00058304 | 0.03449617 |
| CCNG1 | 0.81584613 | 0.00059488 | 0.03501728 |
| SCOC | 1.59222306 | 0.00059746 | 0.03501728 |
| ADRA2B | -1.7629051 | 0.00060947 | 0.03552875 |
| CCNC | 0.84329979 | 0.00061242 | 0.03552875 |
| DGAT1 | -0.5874744 | 0.00061472 | 0.03552875 |
| MMS19 | -0.5997445 | 0.00065076 | 0.03722507 |
| PNRC1 | 0.84224595 | 0.00065339 | 0.03722507 |
| TAPT1 | 0.63060485 | 0.00065584 | 0.03722507 |
| SNX16 | 0.82249411 | 0.00065799 | 0.03722507 |
| LYPLA1 | 1.04328648 | 0.00066277 | 0.03722507 |
| SUZ12 | 0.77215732 | 0.00066644 | 0.03722507 |
| CDK10 | -0.7626849 | 0.0006665 | 0.03722507 |
| BEST1 | -0.9042983 | 0.000672 | 0.03722507 |
| INPP5K | -0.6451346 | 0.0006728 | 0.03722507 |
| PGLS | -0.5133464 | 0.00067651 | 0.03722507 |
| ZNHIT1 | -0.5617422 | 0.00067687 | 0.03722507 |
| TMEM30A | 0.70860939 | 0.00068425 | 0.0373936 |
| TMX1 | 0.55642006 | 0.00068593 | 0.0373936 |
| GRK6 | -0.6410019 | 0.00068983 | 0.03744271 |
| ERGIC2 | 0.7502182 | 0.00069409 | 0.037455 |
| ARMCX6 | -0.8533811 | 0.00070052 | 0.037455 |
| TAF1D | 0.5976753 | 0.00070238 | 0.037455 |
| MOB1A | 0.66765784 | 0.00070248 | 0.037455 |
| TMEM87B | 0.73442477 | 0.00070506 | 0.037455 |
| WASHC4 | 0.66445166 | 0.00071501 | 0.03782279 |
| COMMD10 | 0.7642247 | 0.00071876 | 0.03786086 |
| AL390728.6 | 1.49811579 | 0.00072342 | 0.03794631 |
| RASA2 | 0.65374828 | 0.00072993 | 0.03812735 |
| CARNMT1 | 1.28648928 | 0.0007352 | 0.03824264 |
| SMIM13 | 0.90868764 | 0.0007401 | 0.03833802 |
| TNFSF12 | -0.7467077 | 0.00074817 | 0.03859555 |
| CBFB | 0.82741178 | 0.0007538 | 0.03872626 |
| Mar/07 | 0.50143427 | 0.00076559 | 0.03917035 |
| DNM1 | -0.7266142 | 0.00077822 | 0.03965433 |
| ELMOD3 | -0.8118786 | 0.000782 | 0.0396851 |
| SNF8 | -0.5191061 | 0.00079167 | 0.03994501 |
| IFI27 | -2.1909343 | 0.00079352 | 0.03994501 |
| MIB1 | 0.63432701 | 0.00081989 | 0.04110643 |
| C1orf35 | -1.123227 | 0.00082346 | 0.04112045 |
| UBE2D1 | 1.18172128 | 0.00083075 | 0.04117384 |
| SLC25A33 | 0.90579543 | 0.00083382 | 0.04117384 |
| SUMO2 | 0.56289247 | 0.0008356 | 0.04117384 |
| IL18 | 0.91643506 | 0.00083772 | 0.04117384 |
| TROVE2 | 0.62684304 | 0.00084202 | 0.04122244 |
| LIMS2 | -0.6062657 | 0.00085413 | 0.0415706 |
| YPEL4 | -1.3226453 | 0.00085579 | 0.0415706 |
| AC006978.1 | -0.9364303 | 0.00087379 | 0.04228061 |
| SELENOT | 0.55848557 | 0.00088583 | 0.04269755 |
| TCEA1 | 0.98547943 | 0.0008927 | 0.04286321 |
| PIK3R6 | -0.7741332 | 0.00089899 | 0.0429391 |
| PDZD8 | 0.57091753 | 0.00090116 | 0.0429391 |
| ZDHHC20 | 0.59792542 | 0.00090722 | 0.04306357 |
| ZNF107 | 0.74516811 | 0.0009126 | 0.04315492 |
| SLC39A10 | 0.82158356 | 0.00092168 | 0.04341963 |
| TMEM167B | 0.48533228 | 0.00092866 | 0.0435844 |
| SCYL2 | 0.67425883 | 0.00093552 | 0.04374147 |
| SAV1 | 0.62238173 | 0.00094308 | 0.04382446 |
| ERI1 | 0.5897437 | 0.00094431 | 0.04382446 |
| PCNX4 | 0.55950448 | 0.00096958 | 0.04483043 |
| PPP1R3E | 1.83582492 | 0.00097836 | 0.04497091 |
| AC125257.1 | 1.67966599 | 0.00097982 | 0.04497091 |
| KBTBD2 | 0.51444157 | 0.00100427 | 0.04592399 |
| ABCE1 | 0.78519445 | 0.00100993 | 0.04601431 |
| UBTD2 | 0.54665972 | 0.00101651 | 0.04609567 |
| AKAP5 | 0.62025316 | 0.0010191 | 0.04609567 |
| ELK4 | 0.62981957 | 0.0010236 | 0.04610522 |
| KYAT3 | 0.59431456 | 0.00102669 | 0.04610522 |
| PURB | 0.75829694 | 0.0010361 | 0.04636097 |
| FAM234B | -1.3136422 | 0.00104055 | 0.04639361 |
| AP001330.5 | 3.26885145 | 0.00105279 | 0.04677248 |
| TP53I3 | -0.6049389 | 0.00106642 | 0.04713951 |
| HECA | 0.63187128 | 0.00106861 | 0.04713951 |
| IPMK | 1.17664437 | 0.00108841 | 0.04736168 |
| LYRM7 | 1.15407352 | 0.00108906 | 0.04736168 |
| BCAS2 | 0.83170551 | 0.00108922 | 0.04736168 |
| FAM200A | 1.05121256 | 0.00109068 | 0.04736168 |
| BCAP29 | 0.80350551 | 0.00109755 | 0.04736168 |
| SLC9A6 | 0.56399328 | 0.0010986 | 0.04736168 |
| WASL | 0.76722161 | 0.00110297 | 0.04736168 |
| NRAS | 0.59700046 | 0.00110399 | 0.04736168 |
| SERP1 | 0.64680168 | 0.00111091 | 0.04749539 |
| RALA | 0.56284973 | 0.00111765 | 0.04762048 |
| SURF1 | -0.5561655 | 0.00113142 | 0.04769947 |
| BLOC1S2 | 0.50837023 | 0.00113155 | 0.04769947 |
| UBE2N | 0.50567786 | 0.00113208 | 0.04769947 |
| ASCL2 | -1.0166335 | 0.00113479 | 0.04769947 |
| ZFC3H1 | 0.49247484 | 0.00114313 | 0.04788852 |
| TGDS | 0.98641462 | 0.00114786 | 0.04792596 |
| FEZ2 | 0.52861677 | 0.00116348 | 0.04837009 |
| HAUS2 | 0.60501018 | 0.00116745 | 0.04837009 |
| GALNT1 | 0.48776137 | 0.00117012 | 0.04837009 |
| ETNK1 | 0.78831332 | 0.00117705 | 0.04847437 |
| NDUFA5 | 0.75394978 | 0.00118041 | 0.04847437 |
| RYBP | 0.66505694 | 0.00121454 | 0.04961649 |
| MIER3 | 0.61960073 | 0.00121617 | 0.04961649 |
| EIF4E | 0.83561022 | 0.00122057 | 0.049634 |
| PPP4R3B | 0.51197441 | 0.00122902 | 0.04972659 |
| LONRF1 | 0.63646542 | 0.00123082 | 0.04972659 |
